# Maternal milk microbiota and oligosaccharides contribute to the infant gut microbiota assembly

**DOI:** 10.1038/s43705-021-00021-3

**Published:** 2021-06-07

**Authors:** Martin Frederik Laursen, Ceyda T. Pekmez, Melanie Wange Larsson, Mads Vendelbo Lind, Chloe Yonemitsu, Anni Larnkjær, Christian Mølgaard, Lars Bode, Lars Ove Dragsted, Kim F. Michaelsen, Tine Rask Licht, Martin Iain Bahl

**Affiliations:** 1 National Food Institute, Technical University of Denmark, Kongens Lyngby, Denmark; 2 Department of Nutrition, Exercise, and Sports, University of Copenhagen, Frederiksberg, Denmark; 3 Department of Nursing and Nutrition, University College Copenhagen, Copenhagen, Denmark; 4 Department of Pediatrics and Larsson-Rosenquist Foundation Mother-Milk-Infant Center of Research Excellence, University of California San Diego, La Jolla, CA USA

**Keywords:** Bacteria, Microbial ecology

## Abstract

Breastfeeding protects against diseases, with potential mechanisms driving this being human milk oligosaccharides (HMOs) and the seeding of milk-associated bacteria in the infant gut. In a cohort of 34 mother–infant dyads we analyzed the microbiota and HMO profiles in breast milk samples and infant’s feces. The microbiota in foremilk and hindmilk samples of breast milk was compositionally similar, however hindmilk had higher bacterial load and absolute abundance of oral-associated bacteria, but a lower absolute abundance of skin-associated *Staphylococcus* spp. The microbial communities within both milk and infant’s feces changed significantly over the lactation period. On average 33% and 23% of the bacterial taxa detected in infant’s feces were shared with the corresponding mother’s milk at 5 and 9 months of age, respectively, with *Streptococcus*, *Veillonella* and *Bifidobacterium* spp. among the most frequently shared. The predominant HMOs in feces associated with the infant’s fecal microbiota, and the dominating infant species *B. longum* ssp. *infantis* and *B. bifidum* correlated inversely with HMOs. Our results show that breast milk microbiota changes over time and within a feeding session, likely due to transfer of infant oral bacteria during breastfeeding and suggest that milk-associated bacteria and HMOs direct the assembly of the infant gut microbiota.

## Introduction

Breastfeeding reduces the risk of infectious diseases and may additionally reduce the risk of obesity, metabolic, and immune-related diseases later in life.^1,2^ Breast milk contains all the macro- and micronutrients necessary for infant growth, as well as bioactive compounds such as antimicrobial compounds, immunoglobulins, cytokines, peptide hormones and growth factors.^3,4^ Additionally, research within the last decade has revealed that breast milk contains a diverse microbiota,^5–8^ which may act as a reservoir of microbes for colonization of the infant gut and subsequently impact current and long-term health.^9–11^ The origins of bacteria in human breast milk are not fully elucidated but thought to be maternal skin,^12^ infant oral cavity^13^ and environmental,^7^ as well as the maternal gut and oral cavity through the proposed entero-mammary^14^ and oro-mammary pathways,^15^ respectively. Breast milk from a complete feeding session varies in macronutrient composition and energy content during the feed.^16^ Practically, different fractions from a feeding session can be obtained with the terms *foremilk*, the very first milk when emptying the breast and *hindmilk*, the remainder or last milk when emptying the breast, commonly being used.^16–18^ By sampling and analyzing both fore- and hindmilk from the same feeding session prior to and after nursing the infant at the breast, it is possible to assess the extent to which retrograde inoculation of infant oral bacteria occurs. However, to our knowledge, no previous studies have assessed how the bacterial load and composition changes along a feeding session. It has been estimated that exclusively breastfed infants, by consuming ~800 ml breast milk per day, ingest somewhere between 10^5^ and 10^7^ bacteria daily through breast milk.^10^ However, knowledge on which milk-associated bacterial taxa that establish and colonize the infant gut is scarce, and only a limited number of studies have estimated the extent to which breast milk microbes are transferred to the infant gut longitudinally.^13,19,20^ Especially, information about potential continued milk-to-gut transmission of bacteria spanning the complementary feeding period is lacking. Breast milk also contains a range of human milk oligosaccharides (HMOs), which may impact infant health both directly and indirectly (through selective microbial utilization by infant gut microbes).^21^ The HMOs, which constitute the third largest fraction of breast milk and comprises 5–20 g/l in mature milk, largely reach the intestinal tract undigested and thus become available as substrates for growth of specific gut microbes.^22^ Some individuals harboring a non-functional fucosyltransferase-2 (FUT2) gene, termed non-secretors, have markedly reduced breast milk levels of α1-2-fucosylated HMOs (e.g., 2ʹFL) and previous studies suggest that this impacts the gut microbiota of the breastfed infant.^23–25^ While previous in vitro studies have shown that some groups of gut microbes are capable of degrading HMOs,^26,27^ there is a lack of human studies confirming this. *Bifidobacterium* spp. are archetypical HMO-degraders^28^ and given the dominance of *Bifidobacterium* in the gut of breastfed infants,^29–31^ is it of particular interest to investigate whether seeding of these from breast milk may occur, and whether HMOs contribute to their dominance. Therefore, across the lactation period, we characterized the microbiota in maternal foremilk and hindmilk samples and in infant’s fecal samples and compared these with the HMO profiles in both sample types, in order to assess the impact of maternal milk microbes and HMOs on infant gut microbiota composition.

## Materials and methods

### Cohort subjects

The present study includes maternal breast milk and infant fecal samples from mother–infant pairs enrolled in the SKOT III cohort.^32^ The study protocol was approved by the Regional Ethical Committee of the Capital Region of Denmark in accordance with the Helsinki declaration (H-15008948) and registered at the Danish Data Protection Agency (2015-57-0117 and 2015-57-0116). Written informed consent was obtained from the parents. This cohort consist of breastfed infants that exhibit either excessive weight gain (EWG; *n* = 13, defined as increment of ≥1 SDs in weight-for-age *Z*-score during the first five months postpartum and a weight-for-age *Z*-score > 2.00 at recruitment time) or normal weight gain (NWG; *n* = 17, defined as weight-for-age *Z*-score between −1.00 and +1.00 SDs at first examination) during exclusive breastfeeding. In the present study, we included four additional participants that were previously excluded due to growth patterns, which did not adhere to the definition of growth pattern for the two groups,^32^ as our aim was to assess influence of breast milk microbiome and HMOs on infant gut microbiome, independent of infant growth patterns (Table S1). Recruitment of participants and criteria of inclusion and exclusion have been described previously.^32^ All infants were required to be within 5–6.5 months of age at first visit and at that time have breastfeeding as the primary energy source, allowing maximum two meals per day of solid foods. Infants were seen for a second visit at age 9 months ± 2 weeks. Maternal breast milk samples and infant fecal samples were obtained at both visits (Table S2). Additionally, we obtained information on age, gender, birth mode, gestational age at birth, presence of siblings, breastfeeding practices and use of antibiotics from parental questionnaires as described previously.^32^

### Fecal and breast milk samples and DNA extraction

On the morning of the visits parents collected infant’s fecal samples from diapers and transported them on ice to the Department Nutrition, Exercise and Sports, University of Copenhagen, where they were stored at −80 ^o^C until DNA extraction. Before the first and second visit, the mothers were instructed to collect 10 ml breast milk from the start of the breastfeeding session (foremilk), then breastfeed the infant and subsequently collect 10 ml at the end of the same breastfeeding session (hindmilk). Foremilk and hindmilk samples were collected from the same breast, without cleaning the breast before or in between samplings. However, for three individuals at both visits, foremilk and hindmilk were collected on 2 separate days, 1–5 days apart (Table S2). Milk samples were collected into disposable tubes using a manual breast pump (Type Harmony^TM^, Medela AG, Baar, Switzerland) and stored at −20 °C in participants homes until transportation (on ice) to the University of Copenhagen, where the samples were stored at −80 °C until DNA extraction. In order to avoid fecal microbial contaminants in the breast milk samples, extraction of DNA from infant’s feces and maternal breast milk samples was performed separately on different occasions. Further, a total of 10 blank DNA extraction controls (one for each batch of DNA extraction from milk samples) were included through all the same steps as in the procedure for the milk samples. DNA was extracted from ~200 mg infant feces in random order using the DNeasy PowerLyzer PowerSoil kit (Qiagen, 12855-100) with a few modifications: Bead beating was performed at 30 cycles/s for 10 min (Retsch MM 300 mixer mill) and the initial centrifugation steps were performed at 10,000 × *g* for 3 min, as recommended for clay matter. Prior to DNA extraction of milk samples, ~2 ml was centrifuged at 20,000 × *g* for 2 min in micro-centrifuge tubes, after which the upper fat layer was removed using a pipette tip and the supernatant discarded by inverting the tube. The pellets were then re-suspended in 750 μl bead solution (from the DNeasy PowerLyzer PowerSoil kit) and transferred to a new tube. Total DNA was extracted in random order from these suspensions using the same protocol as for fecal samples. Concentrations of DNA in both feces and breast milk samples were measured by Qubit^®^ dsDNA HS assay (Invitrogen^TM^, Q32851).

### Analysis of human milk oligosaccharides in breast milk and infant feces

#### Human milk oligosaccharides in breast milk

Mothers collected the entire content of both breasts using a manual breast pump at infant age 5 and 9 months, as described above. The HMOs were analyzed in well-mixed samples of right and left breast at the University of California, San Diego. Human milk was spiked with raffinose (a non-HMO carbohydrate) as an internal standard at the beginning of sample preparation to correct for sample losses during sample processing and allow for absolute oligosaccharide quantification. Oligosaccharides were extracted by high-throughput solid phase extraction over C18 (Hypercarb-96, 25 mg bed weight, Thermo Scientific) and Carbograph microcolumns (Hypersep-96 C18, 25 mg bed weight, Thermo Scientific) using a controlled vacuum manifold. Use of high-throughput microcolumns was validated in multiple different ways: (i) establishing parallelism in serial dilutions, (ii) spiking milk with individual HMO standards to determine recovery, and (iii) comparison with direct in-sample derivatization as used by others.^33^ Oligosaccharides were fluorescently labeled with 2-aminobenzamide (2AB, Sigma) in a 96-well thermocycler at 65 °C for exactly 2 h. The reaction was stopped abruptly by reducing the thermocycler temperature to 4 °C. The amount of 2AB was titrated to be in excess to account for the high and variable amount of lactose and other glycans in milk samples. Unreacted 2AB was removed by high-throughput solid phase extraction over silica microcolumns (Hypersep silica, 25 mg bed weight, Thermo Scientific). Labeled oligosaccharides were analyzed by HPLC (Dionex Ultimate 3000, Thermo Scientific) on an amide-80 column (15 cm length, 2 mm inner diameter, 3 μm particle size; Tosoh Bioscience) with a 50-mmol/l ammonium formate–acetonitrile buffer system. Separation was performed at 25 °C and monitored with a fluorescence detector at 360 nm excitation and 425 nm emission. Peak annotation was based on standard retention times of commercially available HMO standards (Sigma, Dextra, Elicityl) and a synthetic HMO library^34^ and offline mass spectrometric analysis on a Thermo LCQ Duo Ion trap mass spectrometer equipped with a Nano-ESI-source. Absolute concentrations were calculated based on HMO standard response curves for each of the annotated HMO (Oligosaccharide detection limit: ~20 pmol, dynamic range between 20 and 5000 pmol; milk samples were diluted accordingly). The total concentration of HMOs was calculated as the sum of the annotated oligosaccharides. HMO-bound fucose was calculated on a molar basis. One mole HMO with one fucose residue counted as one mole HMO-bound fucose while one mole HMO with two or more fucose residues counted as two or more moles of HMO-bound fucose. The same was calculated for HMO-bound sialic acid. Maternal Secretor status was determined by the high abundance (Secretor) or near absence (Non-Secretor) of the HMO 2ʹ-fucosyllactose in the respective milk samples with a cutoff of 100 nmol 2ʹFL per ml. HMO diversity was calculated using the Simpson’s diversity index.

#### Human milk oligosaccharides in feces

A fecal slurry (1:1 v/v feces:water) from 50 ± 2 mg feces was mixed with 1362.5 µl of 96% ethanol and spiked with 60 µl of internal standard mixture of seven compounds^35^ for quality control. The samples were mixed at 60 °C for 2 min in a Thermo mixer (Eppendorf, East Lime, USA) at 1400 rpm, centrifuged for 2 min at 14,000 rpm in an eppendorf centrifuge and the supernatants were filtered through a 0.2 µm filter into the wells of a 2 ml 96-well plate. A blank sample (Solvent A) treated similar to the fecal supernatants, an external standard containing 44 biologically relevant metabolites (metabolomics standard)^35^ and a pooled sample containing equal amounts of each sample were added to unused wells and analyzed repeatedly (every fifteen samples) throughout the sequence for quality control. Quality control samples were used to evaluate possible contamination, and monitoring drifts in mass accuracy, retention time and instrumental sensitivity. The samples were analyzed by ultra performance liquid chromatography (UPLC) coupled with a quadrupole-time of flight mass spectrometer (q-TOF-MS) equipped with electrospray ionization (ESI) (SYNAPT, Waters, Manchester, UK) in positive ionization mode. A reverse phase HSS T3 C18 column (2.1 × 100 mm, 1.8 µm) coupled with a pre-column (VanGuard HSS T3 C18 column (2.1 × 5 mm, 1.8 µm)) was used for chromatographic separation. Experimental conditions are given in Supplementary File 1. The data was preprocessed using MZmine version 2.28.^36^ A subset of samples was used to optimize the preprocessing parameters by estimating, e.g., minimum and maximum peak widths and *m*/*z* precision across peak scans. Optimized preprocessing parameters are given in Supplementary File 2. The data matrix was imported into MATLAB R2015b (The Mathworks, Inc., MA, USA). Features that are present in the blanks, early and late eluting features (RT < 0.3 and RT > 6.4), potential isotope peaks as well as features with implausible masses based on elemental composition were removed from the dataset with an in-house algorithm similar to the one recently published.^37^ Features within 0.01 min RT tolerance for the sequence and having correlation coefficients >0.7 were grouped as belonging to the same compound. The compounds of interest were confirmed using the authentic standards of 2ʹFL, 3FL’, 3ʹSL, 6ʹSL, LNT and LN*n*T (Glycom A/S, Hørsholm, Denmark). The authentic standards were run in MS mode with the same experimental conditions but in a separate sequence, hence a 0.02 min tolerance between sequences. Further, MS/MS experiments were performed with the same experimental conditions as for MS scan, except for the collision-induced dissociation energies of 10, 20, and 30 eV. Parent ion and the major fragment masses were searched in the cleaned dataset with 0.02 Da *m*/*z* and 0.02 min retention time tolerance. Parent ions and the corresponding fragments were grouped together within 0.01 retention time tolerance and at least 0.7 correlation coefficient. Additionally, similarity of peak shapes was inspected for the corresponding fragments of each parent ion. Pooled samples were used for linear correction of signal drift throughout the analysis.^38^ The major fragment peak intensities observed for each compound 2ʹFL/3FL (major fragment *m*/*z*: 163.059 for [M + H]+ : 489.182 with retention time (rt): 0.56 min), LNT/LN*n*T (major fragment *m*/*z*: 366.135 for [M + H]+ : 708,256 with rt: 0.56 min) and 3ʹSL/6ʹSL (major fragment *m*/*z*: 292.103 for [M + H]+ : 634.220 with rt: 0.57 min), when running the authentic standards (Supplementary File 3) were used for infant feces samples to estimate the relative abundances of fucosyllactoses (2ʹFL + 3FL), lacto-N-(*neo*)tetraoses (LNT + LN*n*T) and sialyllactoses (3ʹSL + 6ʹSL) between individuals (Supplementary File 4 and 5).

### Quantitative PCR

Total bacterial load in breast milk samples was estimated by quantitative PCR (qPCR) on DNA extracted from these, using universal primers (341F: 5ʹ-CCTACGGGAGGCAGCAG-3ʹ, 518R: 5ʹ-ATTACCGCGGCTGCTGG-3ʹ, final concentration 0.5 µM each, annealing temperature 60 °C) targeting the V3 region of the 16S rRNA gene. Using species/subspecies-specific primers, absolute abundances of *B. longum* subsp. *longum*^39^ (lon_0274_F: 5ʹ-GAGGCGATGGTCTGGAAGTT-3ʹ, lon_0274_R: 5ʹ-CCACATCGCCGAGAAGATTC-3ʹ, final concentration 0.75 µM each, annealing temperature 50 °C), *B. longum* subsp. *infantis*^40^ (Blon0915F: 5ʹ- CGTATTGGCTTTGTACGCATTT-3ʹ, Blon0915R: 5ʹ-ATCGTGCCGGTGAGATTTAC-3ʹ, final concentration 0.75 µM each, annealing temperature 50 °C) and *B. bifidum*^41^ (BiBIF-1: 5ʹ-CCACATGATCGCATGTGATTG-3ʹ, BiBIF-2: 5ʹ-CCGAAGGCTTGCTCCCAAA-3ʹ, final concentration 0.5 µM each, annealing temperature 60 °C) were estimated by qPCR of DNA extracted from infant fecal samples obtained at age 5 months. Each reaction was performed (in triplicates) with 2 µl template DNA, the specified primer concentrations and 2x SYBR Green I Master Mix solution (LightCycler^®^ 480 SYBR Green I Master, Roche). A total of 10 blank DNA extractions controls, extracted in parallel with breast milk samples, were included as negative controls on plates containing DNA extracted from breast milk. Standard curves were generated from triplicate 10-fold serial dilutions of linearized plasmid (containing 10^0^–10^8^ gene copies/µl), constructed by cloning a PCR amplified 199 bp fragment of the 16S rRNA gene (V3-region) of *E. coli* ATCC 25922^42^ for estimation of total bacterial load in breast milk or by triplicate 10-fold serial dilutions of DNA extracted from pure cultures of *B. longum* subsp. *longum* DSM 20219, *B. longum* subsp. *infantis* DSM 20088 or *B. bifidum* DSM 20456 for quantification of these in infant’s feces. Plates were run on the LightCycler^®^ 480 Instrument II (Roche) with the program including 5 min pre-incubation at 95 °C, followed by 45 cycles with 15 s at 95 °C, 15 s annealing at 50–60 °C and 15 s at 72 °C and a subsequent melting curve analysis including 5 min at 95 °C, 1 min at 65 °C and continuous temperature increase (ramp rate 0.11 °C/s) until 98 °C. Data were analyzed with the LightCycler^®^ 480 Software (v1.5) (Roche).

### Library preparation and 16S rRNA gene amplicon sequencing

PCR amplification for library preparation and subsequent sequencing on the Ion Torrent platform was performed with DNA extracted from infant’s fecal material (*n* = 77), maternal breast milk (*n* = 87) and blank DNA extraction controls (*n* = 6) essentially as previously reported.^43^ Briefly, amplification of the V3-region of the 16S rRNA gene was performed as follows: For fecal samples the PCR mixture consisted of 0.2 µl Phusion High-Fidelity DNA polymerase (ThermoFisher Scientific, F-553L), 4 µl HF-buffer, 0.4 µl dNTP (10 mM of each base), 1 µM forward primer (PBU; 5ʹ-A-adapter-TCAG-barcode-CCTACGGGAGGCAGCAG-3ʹ) and 1 µM reverse primer (PBR; 5ʹ-trP1-adapter-ATTACCGCGGCTGCTGG-3ʹ) and 1–5 ng community fecal DNA in 20 µl total reaction volume. Both primers (TAG Copenhagen A/S) were linked to sequencing adaptors and the forward primer additionally contained a unique 10 bp barcode (Ion Xpress™ Barcode Adapters) for each sample. The PCR program consisted of initial denaturation for 30 s at 98 °C, followed by 24 cycles of 98 °C for 15 s and 72 °C for 30 s, and lastly 72 °C for 5 min to allow final extension before cooling to 4 °C. For milk and blank samples the PCR was performed with 0.08 µl Accuprime Taq polymerase (ThermoFisher Scientific, 12346086), 2 µl 10x Accuprime PCR buffer II, 0.5 µM of each of the abovementioned primers and 1 µl of DNA extracted from milk or blank controls in a total volume of 20 µl. The PCR program consisted of initial denaturation for 120 s at 94 °C, followed by 40 cycles of 94 °C for 20 s, 58 °C for 20s and 68 °C for 30 s, and lastly 68 °C for 5 min to allow final extension before cooling to 4 °C. No-template controls were included for each PCR run, all resulting in less 0.05 ng/µl. The PCR products were purified by use of HighPrep^TM^ PCR Magnetic Beads (MAGBIO^®^, AC-60005) with a 96-well magnet stand (MAGBIO^®^, MyMag 96), according to the manufacturers recommendations. DNA quantity was measured using Qubit^®^ dsDNA HS assay (Invitrogen^TM^, Q32851) and samples were pooled to obtain equimolar libraries and sequenced on three separate chips using the Ion OneTouch^TM^ and Ion PGM systems with a 318-Chip v2 incorporating the Hi-Q chemistry in 200 bp runs. Sequence data has been deposited at NCBI’s Sequence Read Archive under BioProject number PRJNA553872.

### Bioinformatics, data analysis, and presentation

Raw-sequencing reads were de-multiplexed according to barcode and trimmed to remove barcodes and primers, maintaining only reads containing both forward and reverse primers and discarding reads below 125 bp or above 180 bp using the CLC Genomic Workbench (v8.5) software (CLCbio, Qiagen, Aarhus, DK). An amplicon sequence variant (ASV) table including all samples was generated in R (R Core Team (2018). R: A language and environment for statistical computing. R Foundation for Statistical Computing, Vienna, Austria. URL https://www.R-project.org/) using the DADA2 pipeline v1.12.^44^ Briefly, the reads were quality filtered (maxEE = 2, maxN = 0, truncQ = 2), denoised using pooled data and increased homopolymer gap penalty and band size as recommended for Ion Torrent reads. Chimeric sequences were removed and the resulting ASVs were assigned taxonomy using the RDP 16S rRNA database.^45^ In addition, for selected ASVs, nBLAST^46^ analysis was performed in order to confirm taxonomy assigned by the RDP database, and in some cases to explore species level classifications not obtained with RDP. Species level classification/grouping was only adopted/confirmed if the top BLAST hit showed 100% homology to the ASV sequence, and no other species showed 100% homology. Potential reagent contaminating ASVs in the breast milk samples were identified based on the frequency method, using qPCR-estimated bacterial load, within the *decontam* R package software (v1.6.0).^47^ ASVs identified as contaminants (72 out of 575) were then removed from the complete ASV table (with the exception of two ASVs; ASV_7 and ASV_24, which were kept due to their abundance in infant feces, and thus non-likelihood to represent reagent contaminants) prior to downstream community analysis with the QIIME2 software.^48^ Within the QIIME2 environment ASVs assigned to Cyanobacteria/chloroplast or with a frequency <100 reads across all samples were removed and the *core diversity metrics* function was applied with a rarefaction depth of 18,000 reads per sample to generate phylogenetic (weighted and unweighted UniFrac) and non-phylogenetic (Bray–Curtis and Jaccard) distance matrices and principal coordinates, as well as alpha diversity measures (Shannon index, Observed ASVs and Evenness index). The Jaccard similarity index was calculated as (1-Jaccard distance). Using the *rarefy table* function, the ASV table was rarefied to 18,000 reads per sample and collapsed to higher taxonomical levels using the *taxa collapse* function. Relative abundances at all levels were calculated by total sum scaling. Absolute abundances in the milk microbiota dataset was calculated by multiplying qPCR-estimated total bacterial load with the relative abundances for each taxa. A heatmap was generated using the *heatmap.2* function within the R package *gplots* (v3.1.1), using euclidean distances and UPGMA clustering on genus level relative abundance data with a cutoff of 1% average relative abundance within infant feces or maternal hindmilk at either 5 or 9 months of age. Color gradient PCoA plots were generated using the *ggplot* function within the R package *ggplot2* (v3.3.3). All other figures were generated using the GraphPad Prism Software v 8.3 (GraphPad Softeware Inc., La Jolla, CA).

### Statistics

Statistics were performed with QIIME2, R or GraphPad Prism Software. For non-paired data Mann–Whitney *U* or unpaired *t*-tests were used (depending on normality distribution), whereas Wilcoxon-signed rank test or paired *t*-test were used for paired data. For comparisons of more than two groups one-way analysis of variance (ANOVA) with Fisher’s least significant difference (LSD) post hoc test were used. Correlation between variables was assessed by Spearman’s rank correlation analyses. When appropriate, *p*-values were corrected for multiple testing by the false discovery rate (FDR) method.^49^ ANOSIM test (*beta diversity group significance* function, within QIIME2) were used to assess differences between groups in beta diversity distance measures, whereas ADONIS tests (*adonis* function, within R package vegan v2.5-7) were used to evaluate the impact of continuous variables on beta diversity distance measures, in both cases using 999 permutations.

## Results

### Study participant characteristics

Mother–infant dyads included in this study were enrolled as participants in the SKOT III cohort, including two groups of infants experiencing either normal weight gain (NWG) or excessive weight gain (EWG) during exclusive breastfeeding and additionally four participants that were previously excluded due to non-conform growth patterns.^32^ The 34 breastfed infants were all born at term (79.3% vaginally), there was an equal gender distribution and 48.5% the infants had older siblings. Thirteen (38.2%) of the infants were included in the group with excessive weight gain. Antibiotics exposure at time of sampling was limited to two mother–infant dyads, of which one mother received oral antibiotics at the first sampling (infant age 5–6 months) and one infant received oral antibiotics at second sampling (age 9 months). In terms of maternal secretor status, 14.6% of the mothers had highly reduced levels of α1-2-fucosylated HMOs in the breast milk, and were thus classified as non-secretors. Infant feces and maternal breast milk samples (both foremilk and hindmilk samples, collected before and after nursing the infant, respectively) were obtained at infant age 5.8 ± 0.4 and 9.0 ± 0.3 months (Tables S1–2).

### Breast milk samples contain a microbiota distinguishable from negative controls

**Fig. 1:**
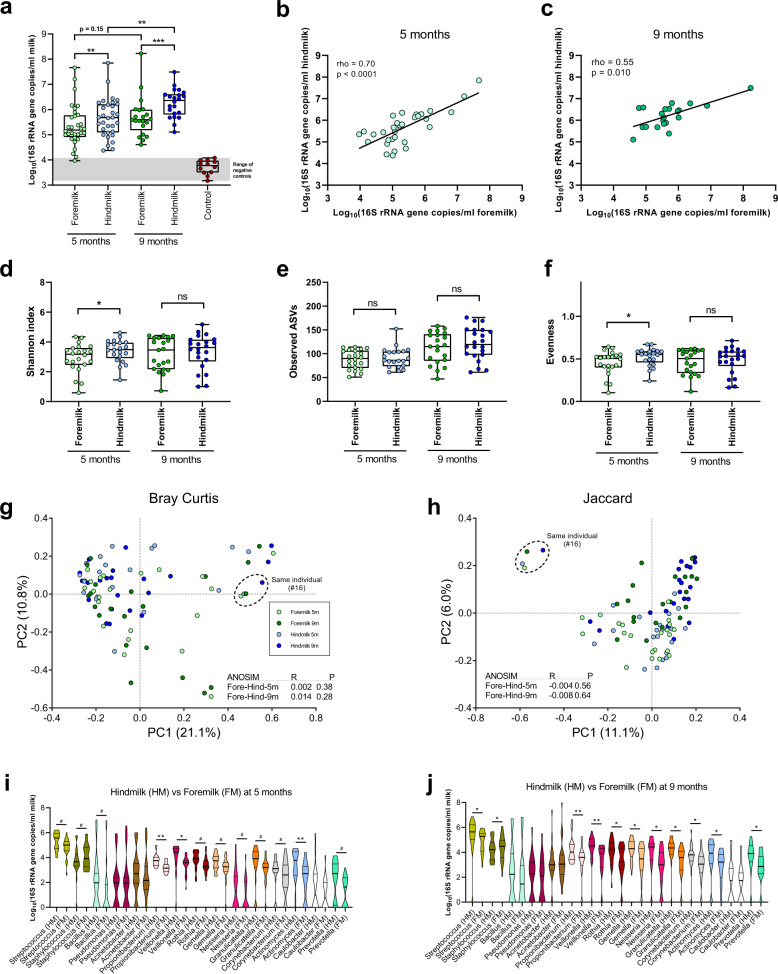
Bacterial load, diversity, and composition in foremilk versus hindmilk. **a** Boxplot (median; IQR; range) of bacterial load in foremilk and hindmilk at infant age 5 and 9 months, as well as DNA blank extraction controls. Statistical significance was evaluated by paired *t*-tests. Asterisks indicate **p < 0.01 and ***p < 0.001. **b**, **c** Scatter dot plots of bacterial load in foremilk versus hindmilk at infant age 5 (**a**) and 9 (**b**) months. A linear regression line was fitted to the log-transformed data and the relationship was evaluated by Spearman’s rank correlation analyses. **d**–**f** Boxplots (Median; IQR; Range) of bacterial alpha diversity measures, Shannon index (**d**), Observed ASVs (**e**) and Evenness index (**f**), comparing foremilk and hindmilk at both infant age 5 and 9 months. Statistical significance was evaluated by paired *t*-tests and asterisks indicate *p < 0.05. **g**, **h** PCoA plots based on Bray–Curtis (**g**) and Jaccard (**h**) dissimilarities, including foremilk and hindmilk samples at both infant age 5 and 9 months, with statistically differential clustering evaluated by ANOSIM tests. **i**, **j** Violin plots of the absolute abundances of the major genera (average relative abundance cutoff across all milk samples of 1.0%) comparing hindmilk to foremilk at infant age 5 months (**i**) and 9 months (**j**). Thick black lines indicate median and thin black lines 25 and 75 percentiles. Statistical difference was evaluated by Wilcoxon-signed rank tests and symbols indicate ^#^FDR*p* = 0.05–0.1, *FDR*p* < 0.05, and **FDR*p* < 0.01.

Since human breast milk is low in microbial biomass,^6,50^ care must be taken during DNA extraction and PCR-based amplification when profiling the bacterial communities in this sample type, due to the risk of reagent or cross-contamination.^51,52^ Therefore, we initially measured bacterial load in both foremilk and hindmilk collected at infant age 5 and 9 months, as well as blank DNA extraction controls (Fig. 1a). The median bacterial loads were at least one order of magnitude higher than those found in DNA extraction negative controls (Fig. 1a). Interestingly, hindmilk samples, taken after feeding the infant at the breast, had a significantly higher bacterial load compared to foremilk samples (taken before the infant was latched at the breast) at both infant age 5 and 9 months, suggesting that a significant transfer of infant oral bacteria occurs during breastfeeding. Bacterial load increased significantly over time in hindmilk, but not in foremilk. We observed clear correlations between the bacterial load in foremilk and hindmilk, obtained from the same individuals, at both 5 months (rho = 0.70, *p* < 0.0001) and 9 months (rho = 0.55, *p* = 0.010) of age (Fig. 1b, c), supporting that the data represents biological variation between individual milk samples, rather than stochastic reagent contamination. We also found significant correlations across time (Fig. S1a, b), within paired samples of hindmilk (rho = 0.54, *p* = 0.01) but not foremilk (rho = 0.18, *p* = 0.43). Next, we profiled the microbial communities residing in the milk samples using 16S rRNA amplicon sequencing. In order to remove any reads potentially originating from reagent contamination we used the *decontam* pipeline,^47^ which filters out amplicon sequence variants (ASVs) with abundances across samples inversely related to the measured bacterial load, since these are highly likely to represent contaminants, as also recently demonstrated for breast milk samples.^51^ This procedure removed 57.5% of the reads in the DNA extraction negative control samples, but only 2.8–11.2% of the reads in the milk samples and <0.5% of the reads in corresponding infant fecal samples (Fig. S2a). The community composition in the remaining reads of the negative control samples was different from those in both maternal milk and infant feces samples (Fig. S2b) and also clustered separately in principal coordinate analysis (PCoA) plots (Fig. S2c–f).

### Foremilk and hindmilk samples contain a similar compositional microbiota differing mainly in absolute abundances of skin-associated versus infant oral-associated bacteria

We found no significant differences in maternal foremilk or hindmilk between infants experiencing EWG and NWG in terms of bacterial load or composition (Fig. S3). We did, however, find a slight increase in alpha diversity measures at 5 months in the EWG group compared to NWG in foremilk, yet this was not consistent as the reverse was observed for hindmilk (Fig. S3). We then compared the microbial communities in the fore- and hindmilk samples irrespective of weight-gain group and found slightly, but significantly higher Shannon index and evenness in the hindmilk compared to foremilk at infant age 5 months but not 9 months (Fig. 1d–f). The hindmilk and foremilk microbial communities were not distinguishable based on Bray–Curtis and Jaccard distance matrices, neither at infant age 5 nor 9 months (Fig. 1g, h). Interestingly, both foremilk and hindmilk samples from the only mother receiving oral antibiotics (#16) at the time of sampling at infant age 5 months displayed a distinct microbiota compared to all other milk samples and this difference was sustained at infant age 9 months (Fig. 1h). Comparison of genus level relative abundances between the two milk sample types (Table S3) revealed that although generally similar, foremilk consistently (at both infant age 5 and 9 months) contained a pronounced and significantly higher relative abundance of *Staphylococcus* (FDR*p* < 0.00001), with the differentially abundant ASVs classified as *S. epidermidis* or *S. hominis* (Table S4), both species recognized as common skin-commensals.^53^ No other bacterial taxa were differentially relative abundant at infant age 5 and 9 months after FDR correction (Tables S3–4). However, when calculating absolute abundances (by multiplying relative abundances for each taxa by qPCR-estimated total bacterial load) we found that actually most bacterial genera, notably many of which are often found as abundant members of the oral microbiota, e.g., *Streptococcus*, *Veillonella*, *Rothia*, *Neisseria*, *Prevotella*, *Gemella, Actinomyces* and *Granulicatella,*^54^ were significantly or borderline significantly more abundant in hindmilk (Fig. 1i, j). In contrast, non-oral-associated bacteria such as *Acinetobacter*, *Pseudomonas* and *Caulobacter*^54^ did not differ significantly. The only genera with higher absolute abundance in foremilk was *Staphylococcus*. Collectively, these observations suggest that a significant amount of infant oral-associated bacteria are transferred to the breast milk when the infant is breastfed and that foremilk harbors a higher proportion of staphylococci likely originating from the breast skin environment. Since hindmilk samples contained a higher bacterial load (thus reduced risk of contaminating reads) and since it seems to be a better representative of the bulk milk-microbiota that the infants receive,^18^ we chose to focus the remainder of our analyses on the hindmilk samples.

### The microbiota of maternal breast milk and infant feces undergo temporal development and are distinct but overlapping in community composition

**Fig. 2:**
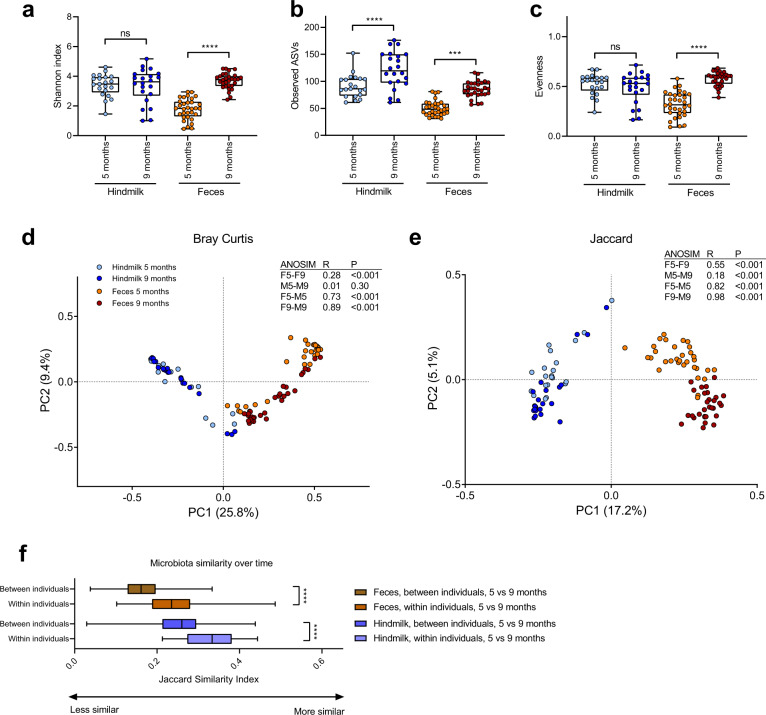
Temporal development of the microbiota in hindmilk and infant’s feces. **a**–**c** Boxplots (Median; IQR; Range) of bacterial alpha diversity measures, Shannon index (**a**), Observed ASVs (**b**), and Evenness index (**c**) in hindmilk and infant’s feces at infant age 5 and 9 months. Statistical significance was evaluated by paired *t*-tests, with asterisks indicating ***p < 0.001 and ****p < 0.0001. **d**, **e** PCoA plots based on Bray–Curtis (**d**) and Jaccard (**e**) dissimilarities, comparing across both sample type and age, with statistical differentially clustering evaluated by ANOSIM tests. **f** Boxplots (median; IQR; range) of microbiota similarity over time in hindmilk and infant’s feces, comparing between to within individuals. Statistical significance evaluated by Mann–Whitney test, with asterisks indicating ****p < 0.0001.

**Fig. 3:**
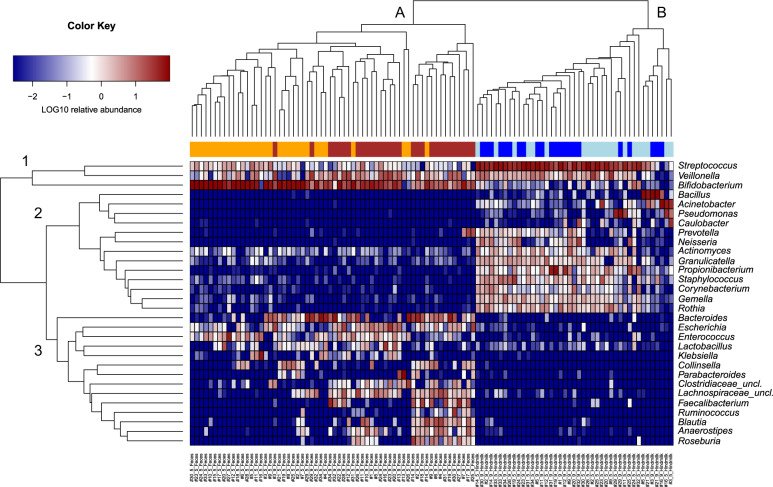
Clustering of bacterial composition in hindmilk and infant’s feces. Heatmap of relative abundances of major genera (>1% average relative abundance in either milk or feces) detected in hindmilk and infant’s feces. Samples cluster according to sample type, **A** infant’s feces and **B** hindmilk. Genera cluster into (1) bacterial genera common to both sample types, (2) bacterial genera most common to hindmilk samples, and (3) bacterial genera most common to infant’s fecal samples. Orange; infant’s feces at infant age 5 months, dark-red; infant’s feces at infant age 9 months, light-blue; maternal hindmilk at infant age 5 months, dark-blue; maternal hindmilk at infant age 9 months.

The bacterial alpha diversity in infant fecal samples increased over time in terms of Shannon Index, number of observed ASVs and evenness, whereas an increase in hindmilk bacterial alpha diversity was only found in terms of the number of observed ASVs (Fig. 2a–c). Temporal changes in the microbial composition of both infant feces and maternal hindmilk were also observed with a clear difference between these two compartments (Fig. 2d, e). We found a higher degree of microbial community similarity at 5 versus 9 months within individuals than between individuals in both compartments (Fig. 2f), highlighting the individuality of both these microbial ecosystems. Cluster analysis of all samples, based on relative abundances of dominant genera, supported both compartmental and temporal differences and further indicated three main clusters of bacterial genera within the samples (Fig. 3). Cluster 1 included genera often found in both sample types, e.g., *Bifidobacterium*, *Streptococcus* and *Veillonella*, suggesting that milk-to-gut transmission of bacteria occurs primarily within these genera. Cluster 2 contained genera found mostly or only in milk (e.g., *Staphylococcus*, *Rothia*, *Gemella*, *Corynebacterium*, *Granulicatella*, *Actinomyces*, *Propionibacterium, Prevotella, Neisseria, Caulobacter, Pseudomonas, Acinetobacter* and *Bacillus*), although both *Actinomyces* and *Granulicatella* were also prevalent, but not abundant, in infant feces. Of note, four genera (*Bacillus*, *Acinetobacter*, *Pseudomonas* and *Caulobacter*) showed highly varying abundance patterns in hindmilk and dominated in a few mothers, but were almost never detected in infant feces. In mother–infant pair #16, where maternal oral antibiotics were consumed at the time of sampling at 5 months, we observed a lack of milk streptococci and instead a strong dominance by *Acinetobacter* and *Bacillus* or *Pseudomonas* at both the 5 and 9 month samplings. The implicated infant had a pronounced depletion of fecal *Bifidobacterium* and was instead dominated by *Klebsiella* at the 5 month sampling. Cluster 3 comprises genera found mainly or only in infant feces (e.g., *Lachnospiraceae*, *Clostridiaceae*, *Roseburia*, *Fecalibacterium*, *Blautia*, *Anaerostipes*, *Collinsella*, *Klebsiella*, *Bacteroides* and *Parabacteroides*) although a few genera in this cluster (e.g., *Escherichia*, *Enterococcus* and *Lactobacillus*) were also occasionally found in hindmilk samples. In infant feces, the relative abundance of *Bifidobacterium* and *Streptococcus* decreased with age (Fig. S4), whereas genera/families from the *Clostridiales* order increased with age (*Ruminococcus*, *Roseburia*, *Faecalibacterium*, *Clostridiaceae*, *Anaerostipes*, *Blautia* and *Lachnospiraceae*). The temporal changes in maternal hindmilk were more subtle and mainly included significant increases in relative abundance of *Prevotella* and *Neisseria* (Fig. S4).

### Maternal breast milk taxa are shared with the infant gut

**Fig. 4:**
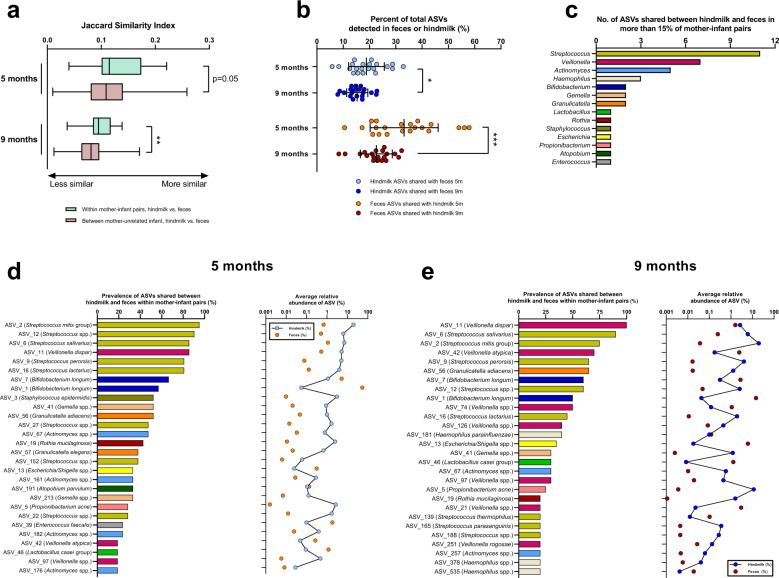
Sharing of bacterial taxa between hindmilk and infant’s feces. **a** Boxplots (median; IQR; range) of microbiota similarity between hindmilk and infant’s feces, comparing within mother–infant pairs to between mothers and unrelated infants at both 5 and 9 months of infant age. Statistical significance was evaluated by unpaired *t*-tests, with asterisks indicating **p < 0.01. **b** Scatter dot plots of percentage of ASVs shared between hindmilk and infant’s feces at 5 and 9 months of infant age. Line and error bars indicate average ± sd. Statistical significance was evaluated by paired *t*-tests with asterisks indicating *p < 0.05 and ***p < 0.001. **c** Barplots of bacterial genera with most ASVs shared between hindmilk and infant’s feces. Only ASVs shared in at least 15% of all mother–infant pairs at either 5 or 9 months of infant age were included. **d**, **e** Bar and dot plots of prevalence (within mother–infant pairs) and average relative abundance of ASVs shared between hindmilk and infant feces, in at least 15% of mother–infant pairs at **d** 5 months and **e** 9 months of infant age.

In order to investigate the potential transmission of bacterial taxa from maternal breast milk to the infant gut, we compared microbial community similarities between related and unrelated mother/infant pairs. At infant age 5 and 9 months, the microbiota similarity was higher within mother–infant pairs than between mothers and unrelated infants for both hindmilk (Fig. 4a) and foremilk (Fig. S5a), suggesting that maternal breast milk microbes are transmitted to the infant gut. On average, 18.8% of the ASVs detected in hindmilk were shared with paired infant feces at 5 months of age, which dropped to 15.3% at 9 months of age (Fig. 4b). Very similar results were obtained using the foremilk microbiota (Fig. S5b), suggesting that there is a slight decrease over time in the fraction of the milk microbiota that is transmitted to the infant gut. We then calculated the percentages of total ASVs detected in infant feces that were shared with hindmilk and found this to be 33.2% at 5 months, but this number significantly decreased to 22.6% at 9 months of age (Fig. 4b). Again, this was very consistent when using the foremilk samples (Fig. S5b). Thus, the contribution of milk microbes to the gut microbial community decreases when the infants start eating other types of food that may also contribute to the gut microbiota. In fact, the actual number of shared ASVs did not significantly change over time (Fig. S6), thus the temporal decrease in percent of shared ASVs reflect the increased richness observed in both milk and infant feces microbiota over time (Fig. 2b), rather than a decrease in actual number of shared ASVs. The ASVs that were most frequently shared (*n* = 40, Table S5) between breast milk and infant feces belonged to the genus *Streptococcus*, followed by *Veillonella*, *Actinomyces*, *Haemophilus, Bifidobacterium, Gemella* and *Granulicatella* (Fig. 4c and Fig. S5c). At infant age 5 months (Fig. 4d and Fig. S5d), we found that sharing of various *Streptococcus* ASVs (e.g., *S. mitis* group, *S. salivarius*, *S. perorsis* and *S. lactarius*) was highly prevalent (>80% of mother–infant pairs). Interestingly, we found two *Bifidobacterium longum* ASVs (ASV_1 and ASV_7) to be shared in >50% of mother–infant pairs. Both had a low relative abundance in hindmilk, but were highly abundant in infant feces (Fig. 4d). We also found evidence of sharing of typical skin-microbes such as *P. acne* (ASV_5) and *S. epidermidis* (ASV_3), although both were not abundant in infant feces. At infant age 9 months, ASVs within the genus *Veillonella* (classified as, e.g., *V. dispar*, *V. atypica* and *V. rogosae*) were more prevalently shared (Fig. 4e and Fig. S5e). Strikingly, while most of the shared ASVs were found in higher proportions in hindmilk compared to feces (e.g., ASVs within *Streptococcus*, *Staphylococcus*, *Gemella*, *Granulicatella* and *Actinomyces*), a few had a much higher relative abundance in the feces than in the hindmilk (e.g., *Bifidobacterium*, *Enterococcus*, *Lactobacillus*, *Escherichia*, and some *Veillonella* ASVs), indicating that the latter were selected for by the gut ecosystem. Given the risk of low abundant hindmilk ASVs to represent reagent contamination, we checked the presence and absolute abundance of the 40 most frequently shared ASVs in sequenced DNA extraction controls, and found that all, except two (ASV_7 and ASV_13), were either not detected in controls (*n* = 19/40) or markedly less abundant (*n* = 19/40) in controls compared to milk (Fig. S7). Owing to our special interest in the two shared *Bifidobacterium longum* ASVs we further investigated these and found that ASV_1, but not ASV_7, was significantly more abundant (*p* = 0.009) in milk samples compared to controls and correlated significantly between paired hindmilk and foremilk samples (rho = 0.31, *p* = 0.048), suggesting genuine presence of this ASV in breast milk. Even though ASVs belonging to the genera *Bacillus*, *Acinetobacter*, and *Pseudomonas* were frequently found in breast milk and were even highly dominating in some samples (Fig. 3), they were never shared with the infant gut, suggesting that they are not able to survive and establish in the infant gut ecosystem. Although typical gut colonizers such as *Bacteroides*, *Parabacteroides* and several genera within *Ruminococcaceae* and *Lachnospiraceae* were sporadically detected in maternal breast milk samples (Fig. 3), specific ASVs within these genera were never shared with infant feces, pointing towards other sources than breast milk for their establishment in the infant gut. Furthermore, we consistently noted a very low amount of shared ASVs in the mother–infant pair where the mother was in treatment with antibiotics at first sampling, even at infant age 9 months (Fig. S6). Altogether, these data suggest that some (incl. *Streptococcus*, *Veillonella*, and *Bifidobacterium* spp.), but not all, maternal breast milk microbes are transmitted to the infant gut and that breast milk is an unlikely source of fecal *Bacteroides*, *Lachnospiraceae*, and *Ruminococcaceae* species.

### Secretor status and human milk oligosaccharide composition in breast milk show limited correlation with gut microbiota composition

Maternal secretor status did not appear to affect the overall infant gut microbial community (Fig. S8a, b). Since in vitro studies have demonstrated that specific *Bifidobacterium* spp. (e.g., *B. longum* subsp. *infantis* and *B. bifidum*) are major consumers of HMOs,^11^ including 2ʹFL,^55^ and an impact of secretor status on the abundance of this genus has previously been reported,^23^ we expected that specific species within this genus would be affected by maternal secretor status. However, we found no significant differences in relative abundances of any of the *Bifidobacterium* ASVs or other ASVs in feces of infants with secretor mothers versus non-secretor mothers (Tables S6 and 7). Neither HMO diversity nor specific HMO concentrations (measured in mixtures of combined content of breast milk from both breasts), including cumulative fucosylated, sialylated and total HMOs, associated with the overall infant fecal microbiota at age 5 months (Table S8) nor specific genera or ASVs (Tables S9 and 10) after correction for multiple testing, with the exception of *Escherichia*/ASV_13 (*Escherichia* spp.) correlating positively with 6ʹSL (FDR*p* < 0.02) and a tendency for negative correlation with fucosylated HMOs (FDR*p* < 0.1). These results imply that secretor status and the specific HMO composition of breast milk may have limited influence on the gut microbiota of the infant, at least at this particular age of 5 months. This could possibly be due to excess HMO amounts in the infant gut and that specific individual HMOs may be redundant for broad-range HMO-utilizers such as *B. bifidum* and *B. longum* subsp. *infantis*.

### Fecal residuals of human milk oligosaccharides associate with the infant gut microbiota composition and explains the abundance of HMO-degrading *Bifidobacterium* species

**Fig. 5:**
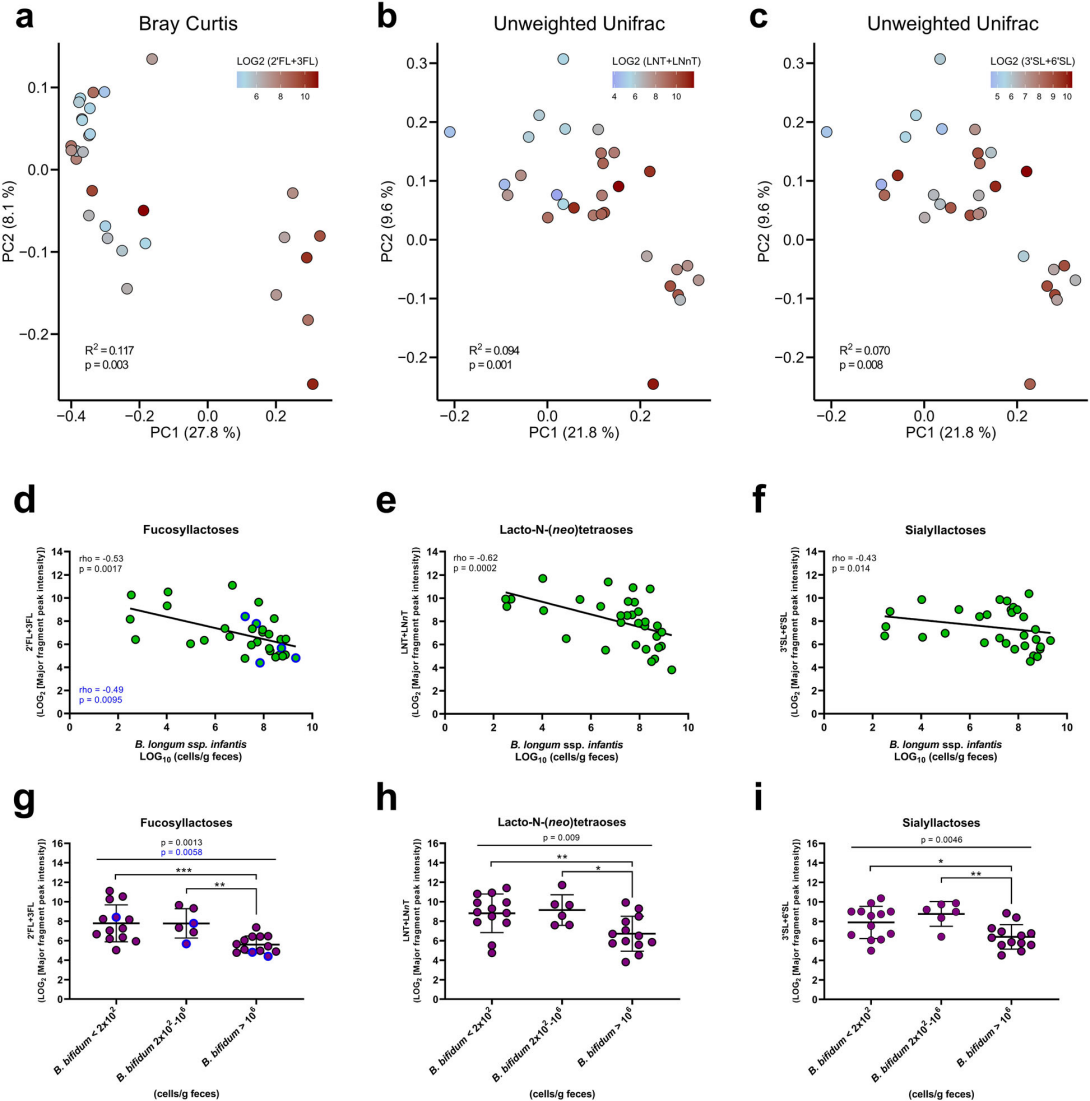
Human milk oligosaccharides associate with infant fecal microbiota composition and explains abundance of specific *Bifidobacterium* species. **a**–**c** PCoA plots based on **a** Bray–Curtis dissimilarities or **b**, **c** unweighted Unifrac distances, colored according to fecal relative abundance (LOG_2_ [major fragment peak intensity]) of **a** 2ʹFL+ 3FL, **b** LNT+ LN*n*T, and **c** 3ʹSL+ 6ʹSL at infant age 5 months. Statistical significance evaluated by ADONIS tests. **d**–**f** Scatter dot plots of qPCR-estimated absolute abundance of *B. longum* subsp. *infantis* versus relative abundance (LOG_2_ [major fragment peak intensity]) of the three HMO classes in feces at infant age 5 months. A linear regression line was fitted to the data points and associations between variables were assessed by Spearman’s rank correlation analyses. **g**–**i** Dot plots of relative abundance (LOG_2_ [major fragment peak intensity]) of the three HMO classes stratified according to qPCR-estimated absolute abundance of *B. bifidum* in feces at infant age 5 months (*B. bifidum* < 2 × 10^2^ cells/g feces indicates values below limit of detection). Statistical significance was evaluated by one-way ANOVA (exact *p*-value indicated) with Fisher’s LSD post hoc tests with asterisks indicating *p < 0.05, **p < 0.01, and ***p < 0.001. For 2ʹFL+ 3FL (fucosyllactoses) statistical analyses (marked in blue text) were repeated excluding infants from non-secretor mothers (dots marked with blue border).

In order to investigate in vivo microbial utilization of HMOs, we compared the fecal relative abundance of fucosyllactoses (2ʹFL+ 3FL), sialyllactoses (3ʹSL+ 6ʹSL) and lacto-N-(*neo*)tetraose (LNT+ LN*n*T) with the infant fecal microbiota at age 5 months. Relative abundances of all three categories of HMOs were significantly associated with microbiota composition (Fig. 5a–c and Table S11). Further analyses revealed that several of the most abundant/prevalent genera and ASVs detected at age 5 months correlated with the relative abundances of HMOs (Table S12 and 13), including *Bifidobacterium*, *Bacteroides, Lactobacillus*, and *Collinsella* (negative associations), as well as *Streptococcus* and *Klebsiella* (positive associations). We focused hereafter on *Bifidobacterium* ASVs, due to their dominance in this cohort, representing on average 69% of the fecal communities and being the dominant genera (>50% of the total community) in 78% of the infants at age 5 months. Among ASVs belonging to *Bifidobacterium*, only ASV_1 (*B. longum*, average relative abundance 54%) and ASV_18 (*B. bifidum*, average relative abundance 2%) correlated negatively with HMO abundances, while the others, including ASV_7 (also assigned to *B. longum*, average relative abundance 5%) did not (Table S13). To differentiate between *B. longum* subsp. *longum* and *B. longum* subsp. *infantis* we performed qPCR using subspecies-specific primers^39,40^ and compared this data to the ASV relative abundances. We found significant positive correlations between ASV_1 and *B. longum* subsp. *infantis* (rho = 0.57, *p* = 0.0006) and between ASV_7 and *B. longum* subsp. *longum* (rho = 0.45, *p* = 0.0095). We also performed qPCR using *B. bifidum*-specific primers and were able to validate the taxonomy of ASV_18, which correlated significantly with *B. bifidum* (rho = 0.82, *p* < 0.00001). Indeed, qPCR-estimated absolute abundances of *B. longum* subsp. *infantis* and *B. bifidum* show significant negative associations with all three HMO categories (Fig. 5d–i), also when excluding infant’s from non-secretor mothers in case of 2ʹFL+ 3FL. Thus, largely in agreement with the literature on genomic potential to degrade HMOs and in vitro evidence of HMOs utilization,^28^ we find that specific *Bifidobacterium* species associate with HMOs in feces, suggesting in vivo HMO degradation and providing a rationale for their dominance in the infant gut.

## Discussion

Our study overall revealed a high degree of similarity in microbial composition of foremilk and hindmilk samples obtained, respectively, before and after breastfeeding the infant. However, interestingly, the hindmilk contained a significantly higher bacterial load at both infant age 5 and 9 months, suggesting that transfer of infant oral bacteria to the milk occurs during breastfeeding. Indeed many oral-associated bacteria such as *Veillonella*, *Streptococcus*, and *Rothia*^54,56^ showed higher absolute abundance in hindmilk than in foremilk at both infant age 5 and 9 months. Conversely, we consistently found a higher relative and absolute abundance of *Staphylococcus* in the foremilk, likely originating from the areolar skin microbiota, which is dominated by this genus.^19^ These findings are supported by a recent study comparing the milk microbiota of breastfeeding mothers who either nursed their infant by latching or provided breast milk in a bottle (never-latched).^12^ Here, the authors found a strong dominance of *Staphylococcus* in breast milk when nursing did not include physical latching, whereas *Streptococcus* and *Veillonella* only appeared with latching.^12^ Thus, it is likely that *Staphylococcus* spp. spread to breast milk primarily from the areolar/nipple skin, whereas breast milk-associated *Streptococcus* and *Veillonella* originate from infant oral cavity.

We observed a clear overlap between hindmilk and infant feces with regards to ASVs classified as *Streptococcus*, *Veillonella*, and *Bifidobacterium*, and found that ~33 and 23 percent of the infant fecal ASVs were shared with the maternal milk at infant ages 5 and 9 months, respectively. Importantly, this does not seem to imply that fewer bacteria are transmitted from milk to gut over time, since we found no differences in the absolute number of ASVs shared within dyads between 5 and 9 months of infant age. However, it seems reasonable that the relative contribution of breast milk microbes to the developing infant gut microbiota decreases gradually over time as the infants is exposed to and colonized with more microbes from diet and the environment (as reflected by the increase in richness from 5 to 9 months of age) and as the diet-related-selective forces (nutrient availability) driving microbial establishment in the gut changes with progression of complementary feeding. Within the ASVs most frequently shared between breast milk and infant feces, *S. mitis* group (e.g., *S. mitis*, *S. oralis* and *S. infantis*), and *S. salivarius* are common oral cavity inhabitants,^57^ *S. perorsis* was first isolated from the infant pharynx^58^ and *S. lactarius* from human breast milk.^59^ *Veillonella* spp. shared such as *V. atypica*, *V. dispar*, and *V. rogosae* are common and abundant members of the oral cavity.^60^ Further, we found sharing of ASVs belonging to *Granulicatella* spp. (e.g., *G. elegans* and *G. adiacens*), *Rothia mucilaginosa*, *Actinomyces* spp., *Gemella* spp. and *Haemophulis* spp., which are also all common oral microbes in infants and toddlers.^54,61^ We cannot exclude the possibility that the presence of these typical oral species in the infant gut results mainly from direct oral-to-gut transmission in the infant,^62^ rather than indirect (through maternal milk). However, it seems plausible that the large amount of bacteria ingested daily contained in the milk, constitutes at least a contributing route for bacteria of suspected oral origin to seed the infant gut. Also, we did find sharing of ASVs belonging to typical skin microbes such as *Staphylococcus epidermidis* and *Propionibacterium acne*. Although typical strict anaerobic gut bacteria, such as *Bacteroides*, *Parabacteroides*, *Faecalibacterium*, *Blautia*, *Roseburia*, and *Anaerostipes* were sporadically detected in breast milk samples, they were never shared with corresponding infant feces, suggesting that breast milk is not a common source of these. However, typical infant gut colonizers *Escherichia*, *Lactobacillus*, *Enterococcus*, and especially *Bifidobacterium* were indeed shared (prevalence between 20 and 70%). These genera are not common inhabitants of the oral cavity^61^ nor human skin,^53^ thus suggesting that not all milk-to-gut transmitted bacteria originate from infant saliva or maternal skin.

We detected ASVs classified as *Bifidobacterium* in ~80% of the breast milk samples. Two *B. longum* ASVs (ASV_1 and ASV_7, indicated by specific qPCR to represent *B. longum* subsp. *longum* and *B. longum* subsp. *infantis*, respectively) were among the top 10 most frequently shared (50–70%), and were both prevalent and highly abundant in the infant fecal communities. Although both these ASVs were also detected in negative sequencing controls, the observed higher absolute abundance in milk samples of ASV_1 compared to sequencing controls and a positive correlation in paired foremilk versus hindmilk samples support the genuine presence of ASV_1 in the milk samples. In contrast, no difference in abundance was found in milk samples versus negative sequencing controls for ASV_7. This specific ASV was identified as a potential contaminant during decontamination filtering but was retained due to the very high abundance in fecal samples. Therefore, it is possible that the observation of ASV_7 in milk samples may be due to contamination. The *B. breve*, *B. bifidum*, and *B. catenulatum* group ASVs were only shared in one or two mother–infant pairs, but also only detected in one to three milk samples at each sampling time (and never in negative sequencing controls). Both *B. longum* and *B. breve* have previously been detected in breast milk in around 80% of individuals, whereas *B. bifidum* and *B. pseudocatenulatum* were previously found in 25% and 15% of individuals, respectively.^63^ Isolation frequency of *Bifidobacterium* species from breast milk typically range from 10 to 30%,^6,64–66^ with the same strains of *Bifidobacterium* species, notably often belonging to *B. longum* (including both subspecies) and *B. breve*, recovered from maternal breast milk and corresponding infant’s feces.^67–72^ Thus, although breast milk-seeding probably does not fully account for the diversity and prevalence of *Bifidobacterium* species in the infant gut, collectively, data support that it is a contributing factor.

Our results suggest that maternal secretor status does not have a major impact on the infant fecal microbiota, although the limited size of our cohort, and especially low a number of non-secretors, prevents us from making statistically solid conclusions. Our results are in line with recent studies showing limited effect of maternal secretor status on infant gut microbiota composition during the first 3–6 months of life,^25,73,74^ although other studies suggest a more marked impact.^23,24^ Further, in our study the concentrations of individual HMOs in breast milk did not correlate strongly with infant gut microbiota composition. Thus, it is possible that efficient HMO consumers will thrive in the intestinal environments of both infants from secretor and non-secretor mothers and somewhat independent of the specific HMO composition in breast milk, since some gut microbes, especially *B. longum* subsp. *infantis* and *B. bifidum*, harbor the gene sets required for degradation of a range of HMO structures from breast milk of non-secretors as well as secretors.^28^ Indeed, these taxa have been shown to be the major in vitro HMO-utilizing *Bifidobacterium* species,^55^ and represent two of the most abundant and prevalent gut species detected in this and other^75^ cohorts of breastfed infants. In accordance with this, we observe significant negative correlations between absolute abundances of *B. longum* subsp. *infantis* and *B. bifidum* and levels of all three major classes of HMOs (fucosylactoses, sialylactoses and lacto-N-(*neo*)tetraoses) in infant’s feces. This supports broad-range HMO utilization within these species in vivo and suggests that HMOs are contributing to the dominance of these species in the breastfed infant’s gut.

Despite the relatively low number of mother–infant dyads included in this study, which limits the power of analysis, we were thus able to uncover several interesting findings. Collectively, our results show that breast milk microbiota composition changes during a feeding session and over the lactation period and that it probably contributes to infant gut seeding of milk-associated bacteria, especially *Streptococcus*, *Veillonella*, and *Bifidobacterium* spp., also during complementary feeding. Along with HMOs, milk-associated bacteria likely direct a specific assembly of the infant gut microbiota, notably dominated by HMO-degrading *Bifidobacterium* species.

## Supplementary information

Supplementary figures

Supplementary tables

Supplementary files
