# Supplementary material for: Maternal milk microbiota and oligosaccharides contribute to the infant gut microbiota assembly
Source: ISME Commun. 2021 Jun 7;1:21. doi: 10.1038/s43705-021-00021-3 (PMC9723702; doi:10.1038/s43705-021-00021-3)
Supplement: Supplementary file 1 — Supplementary figures [file 43705_2021_21_MOESM1_ESM.pdf]

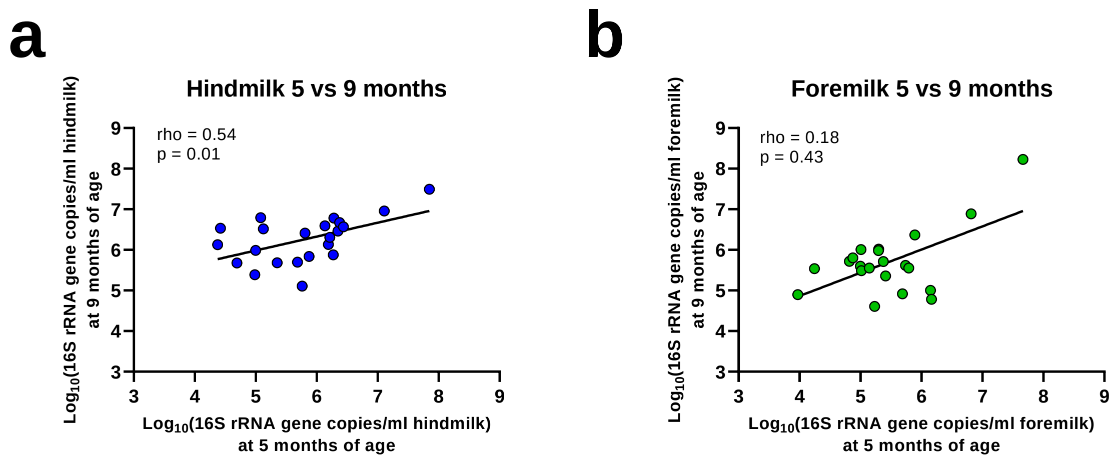


**Figure S1. Stability of bacterial load in breast milk fractions.** a-b) Scatter dot plots of the relationship between bacterial load at 5 versus 9 months of infant age in a) hindmilk and b) foremilk. Linear regression line fitted to the data point and relationship evaluated by Spearman's rank correlation analyses.

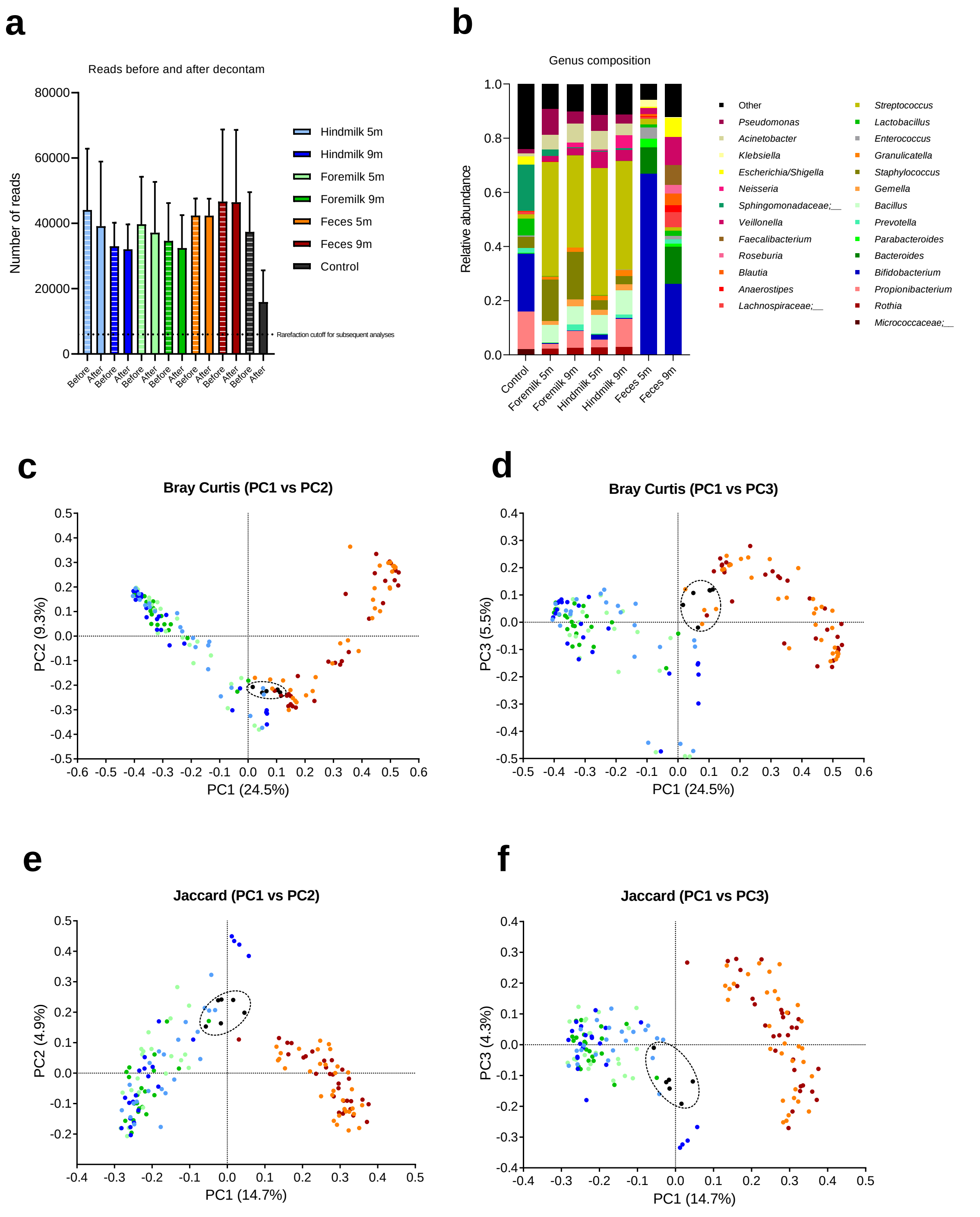


**Figure S2. Decontamination of microbiota samples and comparison to composition in DNA blank extractions.** a) Barplots (average ± sd) of the number of 16 rRNA gene reads (after quality filtering) obtained from hindmilk, foremilk, feces and DNA blank extraction controls before and after decontamination by use of the *decontam* R package. Dotted line indicated the rarefaction cutoff (6000 reads per sample) for subsequent analyses as shown

in panel b-f. b) Barplots illustrating average relative abundances of bacterial genera found in foremilk, hindmilk, infant's feces and DNA blank extraction controls after decontamination and downsizing to 6000 reads per sample. c-f) PCoA plots based on Bray Curtis or Jaccard dissimilarities after decontamination and downsizing to 6000 reads per sample. DNA blank extraction control samples are colored black and marked with a dotted line circle.

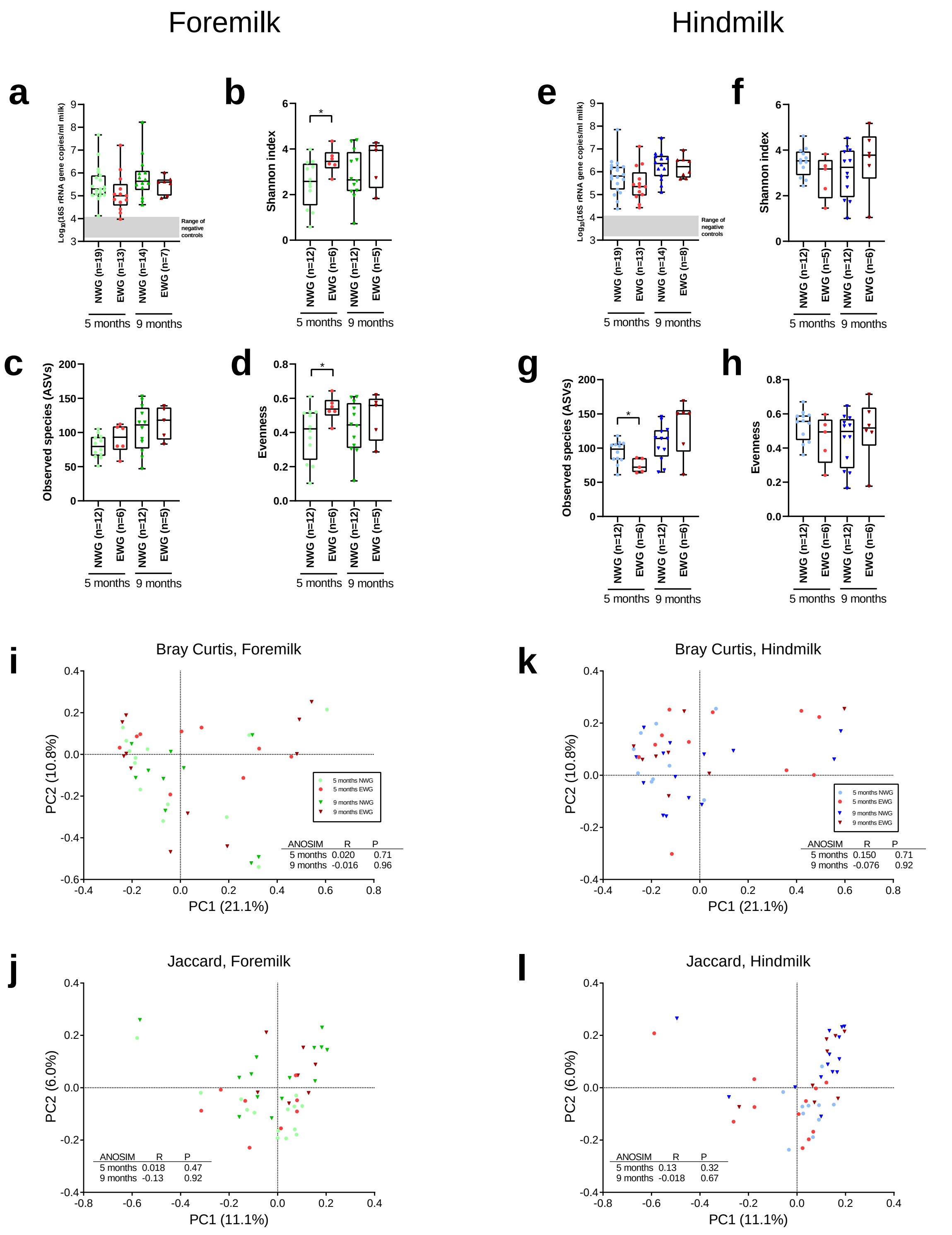


**Figure S3. Comparison of bacterial load and diversity in foremilk and hindmilk between mothers to infants experiencing normal versus excessive weight gain.** a,e) Boxplots (Median; IQR; Range) of bacterial load in foremilk and hindmilk comparing mothers to infants experiencing normal weight gain (NWG) and excessive weight gain (EWG). b-d, f-

h) Boxplots (Median; IQR; Range) of alpha diversity measures in foremilk and hindmilk comparing mothers to infants experiencing NWG and EWG. Statistical significance evaluated by Mann Whitney U tests, with *$p < 0.05$. i-j, k-l) PCoA plots based on Bray Curtis or Jaccard dissimilarities in foremilk and hindmilk comparing mothers to infants experiencing NWG and EWG. Statistical significance evaluated by ANOSIM tests.

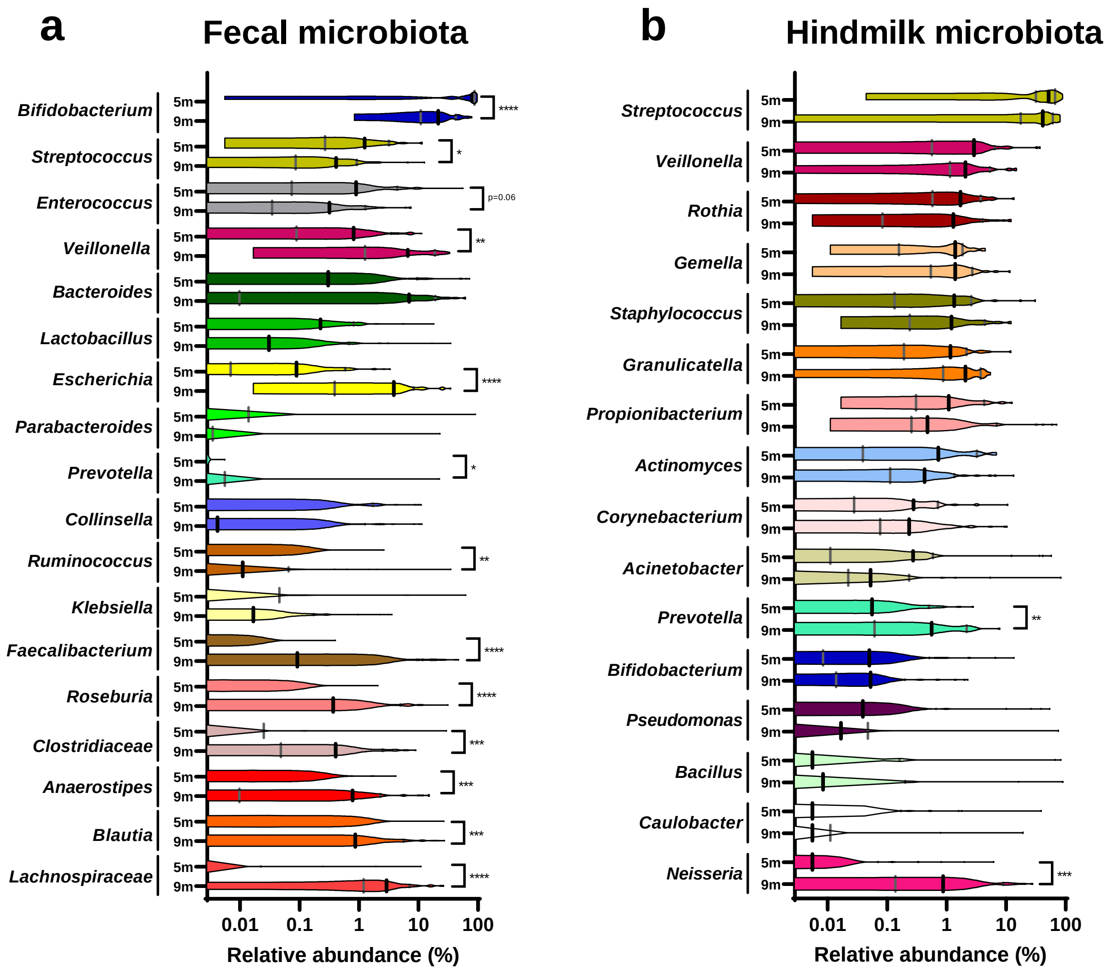


**Figure S4. Temporal development of microbial composition of infant's feces and maternal hindmilk.** a) Violin plots of the relative abundances of the major genera found in infant's feces over time. b) Violin plots of the relative abundances of the major genera found in maternal hindmilk over time. Black lines indicate median and gray lines 25 and 75 percentiles. Statistical significance evaluated by Wilcoxon signed rank test, with asterisks indicating * p < 0.05, ** p < 0.01, *** p < 0.001 and **** p < 0.0001.

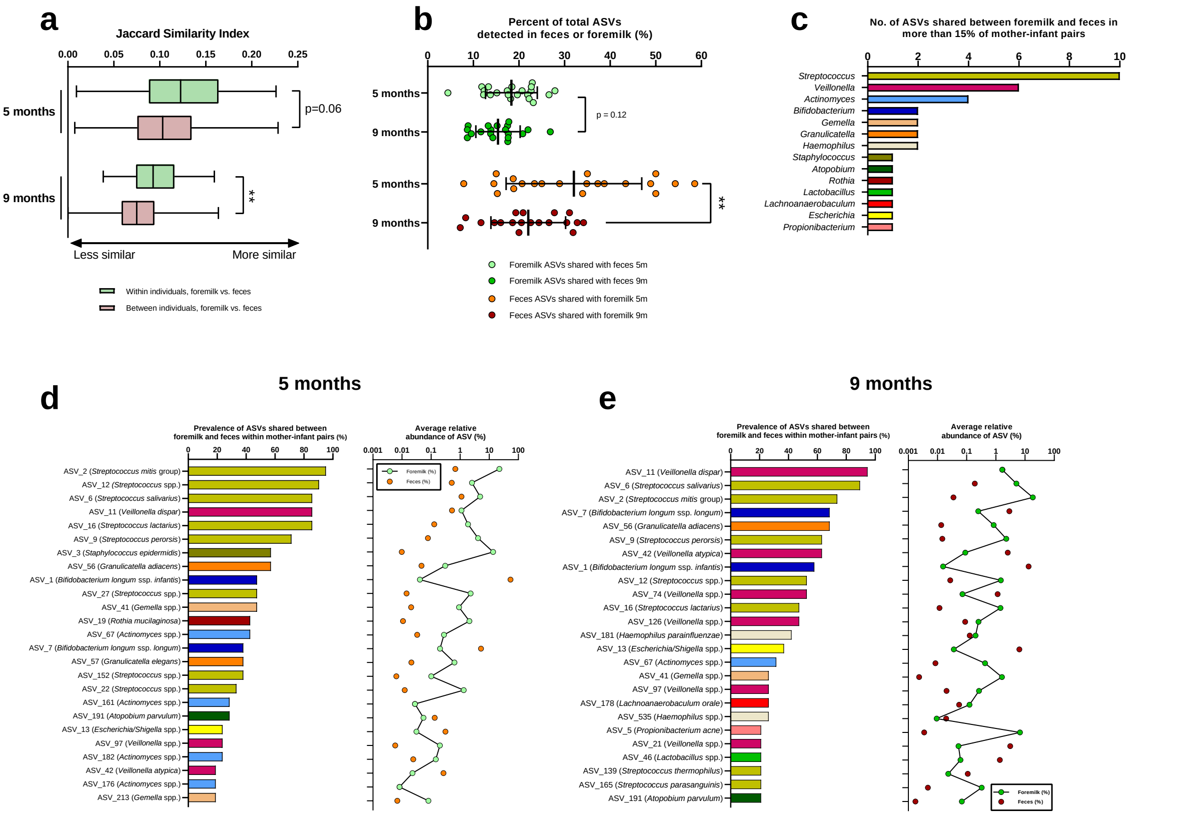


**Figure S5. Sharing of bacterial taxa between foremilk and infant's feces.** a) Boxplots (Median; IQR; Range) of microbiota similarity between foremilk and infant's feces, comparing within mother-infants pairs to between mothers and unrelated infants at both 5 and 9 months of infant age. Statistical significance was evaluated by unpaired *t*-tests, with asterisks indicating ** $p < 0.01$. b) Scatter dot plots of percentage of ASVs shared between foremilk and infant's feces at 5 and 9 months of infant age. Line and error bars indicate average ± sd. Statistical significance was evaluated by paired *t*-tests, with asterisks indicating ** $p < 0.01$. c) Barplots of bacterial genera with most ASVs shared between foremilk and infant's feces. Only ASVs shared in at least 15% of all mother-infant pairs at either 5 or 9 months of infant age were included. d-e) Bar and dot plots of prevalence (within mother-infant pairs) and average relative abundance of ASVs shared between foremilk and infant's feces, in at least 15% of mother-infant pairs at d) 5 months and e) 9 months of infant age.

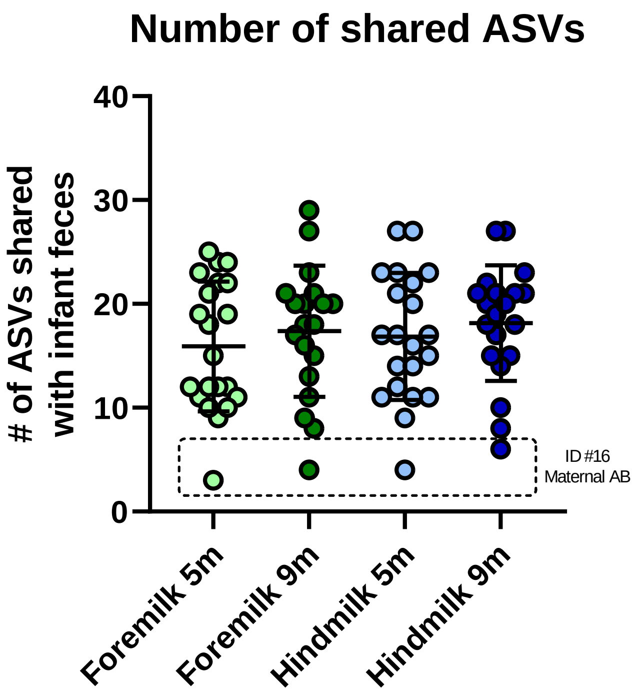


**Figure S6. Number of ASVs shared between maternal breast milk fractions and infant's feces.** Scatter dot plots of the number of ASVs in foremilk and hindmilk shared with infant feces. Line and error bars indicate average ± sd. Mother-infant pair #16, where the mother was treated with oral antibiotics during sampling is indicated with a dotted line.

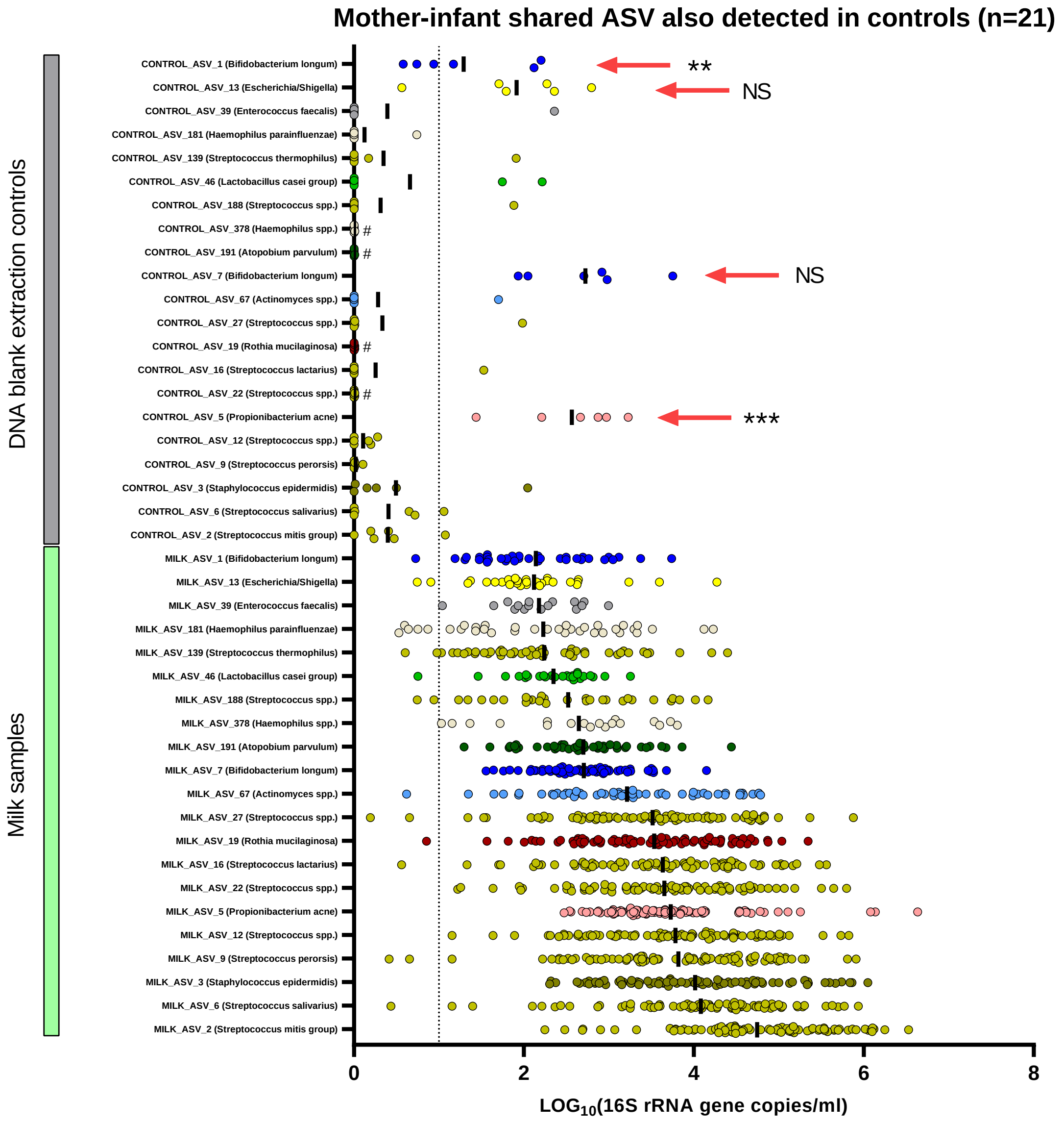


**Figure S7. Mother-infant shared ASVs also detected by in sequencing of DNA blank extraction controls.** Of the 40 ASVs shared between milk samples and infant feces (See Table S5), 21 were also detected in DNA blank extraction controls (# indicates that the ASV was detected in one or two samples just above the limit of detection of $LOG_{10} = 0$). Dots with $LOG_{10}$ values of 0 indicate that the ASV was not detected in the control sample. Scatter dot plots are showing absolute abundance ($LOG_{10}$ 16S rRNA gene copies/ml) of these 21 ASVs in six DNA blank extraction controls and, when detected, in milk samples (both fore- and hindmilk) with the mean indicated with a bold line. Four ASVs (marked with red arrows) were consistently found in controls and had a mean abundance > 1 $LOG_{10}$ 16S rRNA gene copies/ml (dashed line) and were tested for statistical significance, compared to milk samples, by unpaired T-tests with ** $p < 0.01$, *** $p < 0.001$. Only ASV_7 and ASV_13 were as abundant in controls as in milk samples.

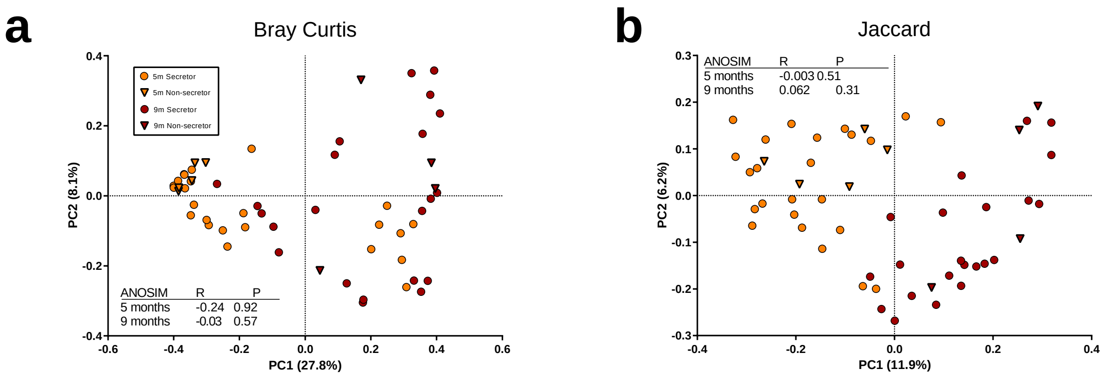


**Figure S8. Comparison of bacterial beta diversity in infant's feces between infants with secretor versus non-secretor mothers.** a-b) PCoA plots based on Bray Curtis or Jaccard dissimilarities in infant feces comparing infants with secretor versus non-secretor mothers. Statistical significance evaluated by ANOSIM tests.
