# Supplementary material for: Maternal milk microbiota and oligosaccharides contribute to the infant gut microbiota assembly
Source: ISME Commun. 2021 Jun 7;1:21. doi: 10.1038/s43705-021-00021-3 (PMC9723702; doi:10.1038/s43705-021-00021-3)
Supplement: Supplementary file 2 — Supplementary tables [file 43705_2021_21_MOESM2_ESM.pdf]

Table S1 - SKOT III cohort characteristics and data

Parameter[1]	SKOT III (n=34)
Cohort metadata	
Maternal age at birth (years, average ± sd)[a]	32.56 ± 3.77
Gestational age at birth (weeks, average ± sd)[c]	40.42 ± 1.06
Age (months, average ± sd)	
1st visit	5.81 ± 0.40
2nd visit[a]	9.03 ± 0.25
Gender	
Males	17/34 (50.0 %)
Females	17/34 (50.0 %)
Mode of birth[c]	
Vaginal	23/29 (79.3 %)
C-section	6/29 (21.7 %)
Excessive weight gain during exclusive breastfeeding[2]	
Yes	13/34 (38.2 %)
No	17/34 (61.8 %)
Breastfeeding	
At 1st visit	34/34 (100 %)
At 2nd visit[a]	31/33 (93.9 %)
Siblings[a]	
No Siblings	17/33 (51.5 %)
1 sibling	12/33 (36.4 %)
2 siblings	4/33 (12.1 %)
Maternal antibiotics	
At 1st visit	1/34 (2.9 %)
At 2nd visit[a]	0/33 (0.0 %)
Infant antibiotics	
At 1st visit	0/34 (0.0 %)
At 2nd visit[a]	1/33 (3.0 %)
Maternal secretor status[b]	
Secretor	27/32 (85.4 %)
Non-secretor	5/32 (14.6 %)
Samples with microbiome data	
Maternal foremilk	
1st visit[b]	22/32 (68.8 %)
2nd visit[f]	21/21 (100 %)
Maternal hindmilk	
1st visit[b]	21/32 (65.6 %)
2nd visit[e]	22/22 (100 %)
Infant feces	
1st visit	32/34 (94.1 %)
2nd visit[a]	30/33 (90.1 %)
Samples with HMO data	
Maternal breast milk (mix of fore- and hindmilk)	
1st visit[b]	32/32 (100 %)
2nd visit[d]	26/26 (100 %)
Infant feces	
1st visit[b]	32/32 (100 %)
2nd visit[a]	33/33 (100 %)

[1]Data or sample missing from a) 1, b) 2, c) 5, d) 8, e) 12 and f) 13 individuals

[2]See definition in Larsson *et al.*, 2018 Nutrients; DOI:10.3390/nu10091290

Table S2 - Overview of samples and data

SKOT III ID	qPCR data					Microbiota data						HMO data			
	Infant age 5 months			Infant age 9 months		Infant age 5 months			Infant age 9 months			Infant age 5 months		Infant age 9 months	
	Feces (n=34)	Foremilk (n=32)	Hindmilk (n=32)	Foremilk (n=21)	Hindmilk (n=22)	Feces (n=32)	Foremilk (n=22)	Hindmilk (n=21)	Feces (n=30)	Foremilk (n=21)	Hindmilk (n=22)	Feces (n=32)	Breast milk (n=32)	Feces (n=33)	Breast milk (n=26)
1										#	#				
2										#	#				
3							#	#							
4															
5							#	#							
6															
7															
8															
9							#	#							
10															
11															
12															
13															
14															
15															
16															
17															
18															
19															
20															
21										#	#				
22															
23															
24															
25															
26															
27															
28															
29															
30															
31															
32															
33															
34															

Colorkey

	Sample analyzed
	Sample not analyzed
	Missing sample
	Sequencing failed or too few reads
#	Fore and hindmilk sampled on seperate days/breastfeeding sessions

Table S3 - Relative abundance of the most abundant genera found in foremilk vs hindmilk at infant age 5 and 9 months

Taxa[1]	Infant age 5 months				Infant age 9 months			
	Average relative abundance in foremilk (%), n=22	Average relative abundance in hindmilk (%), n=21	P-value[2]	FDR corrected P-value	Average relative abundance in foremilk (%), n=21	Average relative abundance in hindmilk (%), n=22	P-value[2]	FDR corrected P-value
Streptococcus	42.208	47.144	0.15360	0.32288	34.053	40.177	0.22900	0.38167
Staphylococcus	15.396	3.641	**<0.00001**	**<0.00001**	17.734	2.996	**<0.00001**	**<0.00001**
Bacillus	6.531	7.182	0.62260	0.71838	6.640	8.886	0.40230	0.58895
Pseudomonas	9.445	6.233	0.37550	0.56325	4.501	3.466	0.11020	0.25157
Acinetobacter	5.367	7.182	0.44730	0.60995	7.001	4.192	**0.00290**	**0.02175**
Propionibacterium	1.792	2.720	0.13270	0.32288	6.080	10.461	0.59130	0.68227
Veillonella	2.186	6.250	**0.00830**	**0.04150**	2.612	4.067	0.11720	0.25157
Rothia	2.156	2.551	0.78410	0.84011	2.422	2.809	0.43190	0.58895
Gemella	1.507	1.462	0.86950	0.86950	2.562	2.200	0.81170	0.81170
Neisseria	0.095	0.450	0.05630	0.16890	1.751	4.831	**0.01240**	0.06200
Granulicatella	1.002	1.852	0.22530	0.37550	1.421	2.175	0.11740	0.25157
Corynebacterium	1.402	1.088	0.57980	0.71838	1.803	1.652	0.76800	0.81170
Actinomyces	0.512	1.838	**0.01640**	0.06150	0.952	1.713	0.22530	0.38167
Caulobacter	0.081	2.061	0.17220	0.32288	1.001	1.022	0.54160	0.67700
Prevotella	0.076	0.381	**0.00450**	**0.03375**	2.258	1.299	0.11110	0.25157

[1]Only taxa with an average relative abundance >1% across milk fractions and age were included

[2]Statistical significance was evaluated by Wilcoxon signed rank test

Table S4 - Relative abundance of the most abundant ASVs found in foremilk vs hindmilk at infant age 5 and 9 months

ASV[1]	Infant age 5 months				Infant age 9 months				RDP taxonomy	nBLAST top hit against NCBI 16S rRNA sequence database[3]	
	Average relative abundance in foremilk (%), n=22	Average relative abundance in hindmilk (%), n=21	P-value[2]	FDR corrected P-value	Average relative abundance in foremilk (%), n=21	Average relative abundance in hindmilk (%), n=22	P-value[2]	FDR corrected P-value		Taxonomy	Identity (%)
ASV_2	21.992	19.723	0.9187	1.0000	18.602	19.950	0.8666	0.9497	Bacteria__Firmicutes__Bacilli__Lactobacillales__Streptococcaceae__Streptococcus		
ASV_3	13.136	3.103	**4.77E-06**	**0.0004**	15.305	2.273	**1.31E-04**	**0.0105**	Bacteria__Firmicutes__Bacilli__Bacillales__Staphylococcaceae__Staphylococcus	***Staphylococcus epidermidis***	**100**
ASV_4	3.902	6.801	0.5073	0.8811	6.312	8.483	0.2549	0.5249	Bacteria__Firmicutes__Bacilli__Bacillales__Bacillaceae_1__Bacillus		
ASV_6	5.695	7.111	0.1031	0.5161	4.620	5.575	0.0967	0.3753	Bacteria__Firmicutes__Bacilli__Lactobacillales__Streptococcaceae__Streptococcus		
ASV_5	1.784	2.683	0.1032	0.5161	6.061	10.427	0.5854	0.7938	Bacteria__Actinobacteria__Actinobacteria__Actinomycetales__Propionibacteriaceae__Propionibacterium__acnes		
ASV_9	4.205	5.037	0.0826	0.5081	2.072	3.672	**0.0421**	0.2593	Bacteria__Firmicutes__Bacilli__Lactobacillales__Streptococcaceae__Streptococcus		
ASV_8	3.894	3.056	0.8885	1.0000	4.406	3.436	0.9645	1.0000	Bacteria__Proteobacteria__Gammaproteobacteria__Pseudomonadales__Pseudomonadaceae__Pseudomonas		
ASV_12	2.570	6.255	**0.0029**	**0.0762**	1.339	2.324	0.1126	0.3753	Bacteria__Firmicutes__Bacilli__Lactobacillales__Streptococcaceae__Streptococcus	*Streptococcus lactarius/perorsis*	99.38
ASV_11	1.062	5.192	**0.0021**	**0.0762**	1.501	2.464	0.1098	0.3753	Bacteria__Firmicutes__Negativicutes__Selenomonadales__Veillonellaceae__Veillonella	***Veillonella diapar***	**100**
ASV_16	2.062	3.993	**0.0063**	**0.1005**	1.307	1.754	0.0545	0.2863	Bacteria__Firmicutes__Bacilli__Lactobacillales__Streptococcaceae__Streptococcus	***Streptococcus lactarius***	**100**
ASV_19	2.113	2.497	0.7795	1.0000	1.421	1.455	0.8348	0.9380	Bacteria__Actinobacteria__Actinobacteria__Actinomycetales__Micrococcaceae__Rothia__mucilaginosa		
ASV_14	1.380	1.435	0.6748	0.9308	2.688	1.823	**0.0044**	**0.0750**	Bacteria__Proteobacteria__Gammaproteobacteria__Pseudomonadales__Moraxellaceae__Acinetobacter	***Acinetobacter johnsonii***	**100**
ASV_22	1.372	1.692	0.2862	0.7830	1.269	2.294	0.0894	0.3753	Bacteria__Firmicutes__Bacilli__Lactobacillales__Streptococcaceae__Streptococcus__peroris		
ASV_27	2.229	1.651	0.2250	0.6923	0.892	1.550	0.1851	0.4540	Bacteria__Firmicutes__Bacilli__Lactobacillales__Streptococcaceae__Streptococcus__mitis		
ASV_41	0.915	0.899	0.8917	1.0000	1.478	1.166	0.5854	0.7938	Bacteria__Firmicutes__Bacilli__Bacillales__Bacillales_Incertae_Sedis_XI__Gemella		
ASV_43	0.081	2.061	0.1816	0.6054	1.001	1.022	0.5097	0.7551	Bacteria__Proteobacteria__Alphaproteobacteria__Caulobacterales__Caulobacteraceae__Caulobacter		
ASV_49	0.004	0.242	0.5176	0.8811	0.621	3.069	0.0608	0.2863	Bacteria__Proteobacteria__Betaproteobacteria__Neisseriales__Neisseriaceae__Neisseria		
ASV_45	2.558	1.243	0.5839	0.8900	0.003	0.001	0.3711	0.6454	Bacteria__Proteobacteria__Gammaproteobacteria__Pseudomonadales__Pseudomonadaceae__Pseudomonas		
ASV_38	0.199	1.968	0.6494	0.9264	1.401	0.268	0.1873	0.4540	Bacteria__Proteobacteria__Gammaproteobacteria__Pseudomonadales__Moraxellaceae__Acinetobacter		
ASV_25	1.129	1.239	0.8888	1.0000	0.729	0.568	0.0464	0.2653	Bacteria__Proteobacteria__Gammaproteobacteria__Pseudomonadales__Moraxellaceae__Acinetobacter		
ASV_31	0.812	0.860	0.7598	0.9964	1.143	0.751	**0.0047**	**0.0750**	Bacteria__Proteobacteria__Gammaproteobacteria__Pseudomonadales__Moraxellaceae__Acinetobacter	*Acinetobacter johnsonii*	99.38
ASV_51	0.291	0.690	0.4483	0.8340	1.800	0.796	0.8442	0.9380	Bacteria__Firmicutes__Bacilli__Lactobacillales__Streptococcaceae__Streptococcus__pneumoniae		
ASV_30	2.275	0.265	0.1258	0.5297	0.314	0.570	0.5692	0.7938	Bacteria__Proteobacteria__Alphaproteobacteria__Sphingomonadales__Sphingomonadaceae		
ASV_56	0.301	0.994	**0.0138**	0.1391	0.773	1.182	**0.0196**	0.1962	Bacteria__Firmicutes__Bacilli__Lactobacillales__Carnobacteriaceae__Granulicatella		
ASV_54	2.562	0.308	0.3491	0.8197	0.025	0.010	0.1775	0.4540	Bacteria__Firmicutes__Bacilli__Bacillales__Bacillaceae_1__Bacillus		
ASV_29	1.684	1.188	0.1169	0.5297	0.035	0.001	0.1411	0.4516	Bacteria__Proteobacteria__Gammaproteobacteria__Pseudomonadales__Pseudomonadaceae__Pseudomonas		
ASV_57	0.641	0.668	1.0000	1.0000	0.547	0.862	0.5392	0.7843	Bacteria__Firmicutes__Bacilli__Lactobacillales__Carnobacteriaceae__Granulicatella__elegans		
ASV_62	0.697	0.148	**0.0052**	**0.1005**	1.356	0.234	**0.0003**	**0.0109**	Bacteria__Firmicutes__Bacilli__Bacillales__Staphylococcaceae__Staphylococcus__epidermidis	***Staphylococcus epidermidis***	**100**
ASV_94	1.905	0.020	0.5294	0.8823	0.257	0.003	0.0583	0.2863	Bacteria__Proteobacteria__Betaproteobacteria__Burkholderiales__Burkholderiales_incertae_sedis__Aquabacterium__commune		
ASV_67	0.266	0.775	0.1242	0.5297	0.387	0.572	0.1556	0.4540	Bacteria__Actinobacteria__Actinobacteria__Actinomycetales__Actinomycetaceae__Actinomyces__odontolyticus		
ASV_82	0.196	0.176	0.7223	0.9630	1.080	0.373	0.8015	0.9380	Bacteria__Actinobacteria__Actinobacteria__Actinomycetales__Corynebacteriaceae__Corynebacterium__kroppenstedtii		
ASV_7	0.205	1.107	0.3838	0.8197	0.225	0.287	0.7022	0.8512	Bacteria__Actinobacteria__Actinobacteria__Bifidobacteriales__Bifidobacteriaceae__Bifidobacterium__longum		
ASV_75	0.532	0.393	0.9039	1.0000	0.328	0.483	0.8813	0.9517	Bacteria__Firmicutes__Negativicutes__Selenomonadales__Veillonellaceae		
ASV_91	0.027	0.235	0.2408	0.7135	0.380	0.944	**0.0141**	0.1610	Bacteria__Bacteroidetes__Bacteroidia__Bacteroidales__Prevotellaceae__Prevotella__melaninogenica		
ASV_102	0.015	0.031	0.1422	0.5418	1.371	0.022	0.6219	0.8292	Bacteria__Bacteroidetes__Bacteroidia__Bacteroidales__Prevotellaceae__Prevotella		
ASV_78	0.195	0.652	0.1705	0.5929	0.280	0.276	0.1633	0.4540	Bacteria__Proteobacteria__Gammaproteobacteria__Pseudomonadales__Moraxellaceae__Enhydrobacter__aerosaccus		
ASV_92	0.300	0.643	0.3518	0.8197	0.247	0.195	0.9653	1.0000	Bacteria__Actinobacteria__Actinobacteria__Actinomycetales__Corynebacteriaceae__Corynebacterium		
ASV_97	0.192	0.476	0.0503	0.4028	0.238	0.407	0.4443	0.7268	Bacteria__Firmicutes__Negativicutes__Selenomonadales__Veillonellaceae__Veillonella		
ASV_113	0.067	0.095	0.3430	0.8197	0.398	0.741	0.1834	0.4540	Bacteria__Proteobacteria__Betaproteobacteria__Neisseriales__Neisseriaceae__Neisseria		
ASV_95	0.681	0.093	0.0608	0.4053	0.450	0.078	**0.0337**	0.2368	Bacteria__Firmicutes__Bacilli__Bacillales__Staphylococcaceae__Staphylococcus	***Staphylococcus hominis***	**100**
ASV_101	0.019	0.004	0.4726	0.8594	0.440	0.833	0.2945	0.5479	Bacteria__Actinobacteria__Actinobacteria__Actinomycetales__Micrococcaceae__Rothia__mucilaginosa		
ASV_87	0.656	0.592	0.5896	0.8900	0.004	0.001	0.2463	0.5249	Bacteria__Proteobacteria__Alphaproteobacteria__Rhizobiales__Rhizobiaceae__Rhizobium		
ASV_119	0.022	0.061	0.3711	0.8197	0.279	0.834	0.1003	0.3753	Bacteria__Actinobacteria__Actinobacteria__Actinomycetales__Corynebacteriaceae__Corynebacterium__argentoratense		
ASV_90	0.306	0.256	0.6600	0.9264	0.144	0.381	0.2559	0.5249	Bacteria__Bacteroidetes__Bacteroidia__Bacteroidales__Prevotellaceae__Alloprevotella		
ASV_118	0.005	0.020	1.0000	1.0000	0.544	0.505	0.7595	0.9068	Bacteria__Actinobacteria__Actinobacteria__Actinomycetales__Micrococcaceae__Rothia		
ASV_125	0.058	0.012	0.6493	0.9264	0.346	0.643	0.0828	0.3681	Bacteria__Firmicutes__Bacilli__Lactobacillales__Streptococcaceae__Streptococcus__sanguinis		
ASV_96	0.404	0.339	0.3974	0.8197	0.285	0.008	0.2885	0.5479	Bacteria__Proteobacteria__Alphaproteobacteria__Sphingomonadales__Sphingomonadaceae__Sphingomonas		
ASV_106	0.415	0.235	0.8960	1.0000	0.156	0.206	0.6580	0.8490	Bacteria__Firmicutes__Negativicutes__Selenomonadales__Veillonellaceae__Veillonella		
ASV_128	0.022	0.021	0.7214	0.9630	0.567	0.393	0.6832	0.8497	Bacteria__Firmicutes__Bacilli__Bacillales__Bacillales_Incertae_Sedis_XI__Gemella		
ASV_127	0.001	0.081	0.4098	0.8197	0.409	0.437	0.8160	0.9380	Bacteria__Fusobacteria__Fusobacteriia__Fusobacteriales__Leptotrichiaceae__Streptobacillus		
ASV_131	0.426	0.317	0.2220	0.6923	0.116	0.059	0.2330	0.5249	Bacteria__Firmicutes__Bacilli__Bacillales__Bacillales_Incertae_Sedis_XI__Gemella		
ASV_149	0.000	0.000	1.0000	1.0000	0.458	0.436	0.8339	0.9380	Bacteria__Proteobacteria__Betaproteobacteria__Neisseriales__Neisseriaceae__Neisseria		
ASV_110	0.001	0.043	1.0000	1.0000	0.108	0.691	0.2661	0.5322	Bacteria__Fusobacteria__Fusobacteriia__Fusobacteriales__Fusobacteriaceae__Cetobacterium		
ASV_137	0.372	0.228	0.9057	1.0000	0.064	0.087	0.4017	0.6837	Bacteria__Bacteroidetes__Bacteroidia__Bacteroidales__Prevotellaceae__Alloprevotella		
ASV_151	0.000	0.004	0.3711	0.8197	0.065	0.655	0.3107	0.5650	Bacteria__Fusobacteria__Fusobacteriia__Fusobacteriales__Leptotrichiaceae__Leptotrichia		
ASV_154	0.081	0.198	0.2553	0.7295	0.140	0.304	0.1819	0.4540	Bacteria__Bacteroidetes__Bacteroidia__Bacteroidales__Porphyromonadaceae		
ASV_162	0.034	0.001	**0.0422**	0.3747	0.696	0.004	0.1508	0.4540	Bacteria__Proteobacteria__Alphaproteobacteria__Sphingomonadales__Sphingomonadaceae__Sphingomonas		
ASV_126	0.035	0.028	0.4227	0.8247	0.230	0.414	**0.0249**	0.2210	Bacteria__Firmicutes__Negativicutes__Selenomonadales__Veillonellaceae__Veillonella		
ASV_152	0.151	0.060	0.3375	0.8197	0.264	0.230	0.4452	0.7268	Bacteria__Firmicutes__Bacilli__Lactobacillales__Streptococcaceae__Streptococcus		
ASV_148	0.249	0.444	0.9325	1.0000	0.012	0.001	0.2785	0.5434	Bacteria__Proteobacteria__Gammaproteobacteria__Pseudomonadales__Pseudomonadaceae__Pseudomonas		
ASV_167	0.182	0.113	1.0000	1.0000	0.364	0.036	0.3711	0.6454	Bacteria__Firmicutes__Bacilli__Lactobacillales__Streptococcaceae__Streptococcus__urinalis		
ASV_157	0.457	0.035	0.1422	0.5418	0.103	0.060	0.5746	0.7938	Bacteria__Actinobacteria__Actinobacteria__Actinomycetales__Corynebacteriaceae__Corynebacterium__kroppenstedtii		
ASV_164	0.033	0.077	0.2936	0.7830	0.207	0.344	**0.0294**	0.2352	Bacteria__Firmicutes__Bacilli__Bacillales__Bacillales_Incertae_Sedis_XI__Gemella__sanguinis		
ASV_109	0.195	0.227	0.4406	0.8340	0.146	0.095	0.5044	0.7551	Bacteria__Proteobacteria__Gammaproteobacteria__Pseudomonadales__Moraxellaceae__Acinetobacter		
ASV_165	0.008	0.005	0.6432	0.9264	0.297	0.325	0.4561	0.7298	Bacteria__Firmicutes__Bacilli__Lactobacillales__Streptococcaceae__Streptococcus		
ASV_103	0.164	0.216	0.9441	1.0000	0.169	0.084	0.1064	0.3753	Bacteria__Proteobacteria__Gammaproteobacteria__Pseudomonadales__Moraxellaceae__Acinetobacter__johnsonii		
ASV_166	0.397	0.102	**0.0089**	0.1188	0.110	0.011	**0.0355**	0.2368	Bacteria__Firmicutes__Bacilli__Bacillales__Staphylococcaceae__Staphylococcus	***Staphylococcus epidermidis***	**100**
ASV_173	0.034	0.026	0.5474	0.8900	0.309	0.243	1.0000	1.0000	Bacteria__Firmicutes__Bacilli__Lactobacillales__Streptococcaceae__Streptococcus		
ASV_161	0.027	0.288	0.0580	0.4053	0.117	0.179	0.6377	0.8364	Bacteria__Actinobacteria__Actinobacteria__Actinomycetales__Actinomycetaceae__Actinomyces__odontolyticus		
ASV_172	0.167	0.021	**0.0139**	0.1391	0.208	0.197	0.6827	0.8497	Bacteria__Firmicutes__Bacilli__Bacillales__Staphylococcaceae__Staphylococcus	***Staphylococcus epidermidis***	**100**
ASV_153	0.560	**0.000**	0.0975	0.5161	0.006	0.002	0.4778	0.7351	Bacteria__Proteobacteria__Gammaproteobacteria__Pseudomonadales__Pseudomonadaceae__Pseudomonas		
ASV_182	0.138	0.392	0.4017	0.8197	0.015	0.032	0.4756	0.7351	Bacteria__Actinobacteria__Actinobacteria__Actinomycetales__Actinomycetaceae__Actinomyces		
ASV_139	0.295	0.218	0.5693	0.8900	0.023	0.014	0.6904	0.8497	Bacteria__Firmicutes__Bacilli__Lactobacillales__Streptococcaceae__Streptococcus__salivarius		
ASV_232	0.504	0.001	1.0000	1.0000	0.002	0.016	1.0000	1.0000	Bacteria__Firmicutes__Bacilli__Lactobacillales__Streptococcaceae__Streptococcus		
ASV_176	0.008	0.029	0.1533	0.5575	0.097	0.387	**0.0016**	**0.0439**	Bacteria__Actinobacteria__Actinobacteria__Actinomycetales__Actinomycetaceae__Actinomyces		
ASV_183	0.109	0.079	0.4858	0.8636	0.132	0.174	0.2120	0.4989	Bacteria__Firmicutes__Bacilli__Lactobacillales__Streptococcaceae__Streptococcus__sanguinis		
ASV_185	0.000	0.001	1.0000	1.0000	0.178	0.251	1.0000	1.0000	Bacteria__Firmicutes__Bacilli__Bacillales__Bacillaceae_1__Bacillus		
ASV_188	0.037	0.037	1.0000	1.0000	0.102	0.251	0.2552	0.5249	Bacteria__Firmicutes__Bacilli__Lactobacillales__Streptococcaceae__Streptococcus		
ASV_169	0.075	0.042	0.7893	1.0000	0.136	0.164	0.8923	0.9517	Bacteria__Proteobacteria__Gammaproteobacteria__Pseudomonadales__Moraxellaceae__Acinetobacter		
ASV_206	0.004	0.000	0.5862	0.8900	0.077	0.317	**0.0135**	0.1610	Bacteria__Fusobacteria__Fusobacteriia__Fusobacteriales__Fusobacteriaceae__Fusobacterium		

[1]Only ASVs with an average relative abundance >0.1% across milk fractions and age were included

[2]Statistical significance was evaluated by Wilcoxon signed rank test

[3]Species level classification of selected differentially abundant ASVs only adopted if top nBLAST hit showed 100% homology and second hit showed <100% homology, as marked with bold text

Table S5 - ASVs shared between breast milk and infant feces in mother infant pairs

ASV (Assigned taxonomy)[1]	Shared with corresponding infant feces				RDP taxonomy	nBLAST top hit against NCBI 16S rRNA sequence database[2]	
	Foremilk 5m (n=22)	Hindmilk 5m (n=21)	Foremilk 9m (n=21)	Hindmilk 9m (n=22		Taxonomy	Identity (%)
ASV_1 (*Bifidobacterium longum*)					Bacteria__Actinobacteria__Actinobacteria__Bifidobacteriales__Bifidobacteriaceae__Bifidobacterium__longum	***Bifidobacterium longum***	**100**
ASV_2 (*Streptococcus mitis* group)					Bacteria__Firmicutes__Bacilli__Lactobacillales__Streptococcaceae__Streptococcus	***Streptococcus mitis* group**	**100**
ASV_3 (*Staphylococcus epidermidis*)					Bacteria__Firmicutes__Bacilli__Bacillales__Staphylococcaceae__Staphylococcus	***Staphylococcus epidermidis***	**100**
ASV_5 (*Propionibacterium acne*)					Bacteria__Actinobacteria__Actinobacteria__Actinomycetales__Propionibacteriaceae__Propionibacterium__acnes	***Propionibacterium acne***	**100**
ASV_6 (Streptococcus salivarius)					Bacteria__Firmicutes__Bacilli__Lactobacillales__Streptococcaceae__Streptococcus	***Streptococcus salivarius***	**100**
ASV_7 (*Bifidobacterium longum*)					Bacteria__Actinobacteria__Actinobacteria__Bifidobacteriales__Bifidobacteriaceae__Bifidobacterium__longum	***Bifidobacterium longum***	**100**
ASV_9 (*Streptococcus perorsis*)					Bacteria__Firmicutes__Bacilli__Lactobacillales__Streptococcaceae__Streptococcus	***Streptococcus perorsis***	**100**
ASV_11 (*Veillonella dispar*)					Bacteria__Firmicutes__Negativicutes__Selenomonadales__Veillonellaceae__Veillonella	***Veillonella dispar***	**100**
ASV_12 (*Streptococcus* spp.)					Bacteria__Firmicutes__Bacilli__Lactobacillales__Streptococcaceae__Streptococcus	*Streptococcus lactarius/perorsis*	99.38
ASV_13 (*Escherichia/Shigella* spp.)					Bacteria__Proteobacteria__Gammaproteobacteria__Enterobacteriales__Enterobacteriaceae__Escherichia/Shigella	Multiple *Escherichia/Shigella* spp.	100
ASV_16 (*Streptococcus lactarius*)					Bacteria__Firmicutes__Bacilli__Lactobacillales__Streptococcaceae__Streptococcus	***Streptococcus lactarius***	**100**
ASV_19 (*Rothia mucilaginosa*)					Bacteria__Actinobacteria__Actinobacteria__Actinomycetales__Micrococcaceae__Rothia__mucilaginosa	***Rothia mucilaginosa***	**100**
ASV_21 (*Veillonella* spp.)					Bacteria__Firmicutes__Negativicutes__Selenomonadales__Veillonellaceae__Veillonella	*Veillonella dispar/tubetsuensis*	99.38
ASV_22 (*Streptococcus* spp.)					Bacteria__Firmicutes__Bacilli__Lactobacillales__Streptococcaceae__Streptococcus__peroris	*Streptococcus lactarius/perorsis*	99.38
ASV_27 (*Streptococcus* spp.)					Bacteria__Firmicutes__Bacilli__Lactobacillales__Streptococcaceae__Streptococcus__mitis	*Streptococcus mitis* group	99.38
ASV_39 (*Enterococcus faecalis*)					Bacteria__Firmicutes__Bacilli__Lactobacillales__Enterococcaceae__Enterococcus	***Enterococcus faecalis***	**100**
ASV_41 (*Gemella* spp.)					Bacteria__Firmicutes__Bacilli__Bacillales__Bacillales_Incertae_Sedis_XI__Gemella	*Gemella taiwanensis/haemolysans*	100
ASV_42 (*Veillonella atypica*)					Bacteria__Firmicutes__Negativicutes__Selenomonadales__Veillonellaceae__Veillonella	***Veillonella atypica***	**100**
ASV_46 (*Lactobacillus casei* group)					Bacteria__Firmicutes__Bacilli__Lactobacillales__Lactobacillaceae__Lactobacillus	***Lactobacillus casei* group.**	**100**
ASV_56 (*Granulicatella adiacens*)					Bacteria__Firmicutes__Bacilli__Lactobacillales__Carnobacteriaceae__Granulicatella	***Granulicatella adiacens***	**100**
ASV_57 (*Granulicatella elegans*)					Bacteria__Firmicutes__Bacilli__Lactobacillales__Carnobacteriaceae__Granulicatella__elegans	***Granulicatella elegans***	**100**
ASV_67 (*Actinomyces* spp.)					Bacteria__Actinobacteria__Actinobacteria__Actinomycetales__Actinomycetaceae__Actinomyces__odontolyticus	*Actinomyces meyeri/odontolyticus*	98.69
ASV_74 (*Veillonella* spp.)					Bacteria__Firmicutes__Negativicutes__Selenomonadales__Veillonellaceae__Veillonella__dispar	*Veillonella atypica*	99.38
ASV_97 (*Veillonella* spp.)					Bacteria__Firmicutes__Negativicutes__Selenomonadales__Veillonellaceae__Veillonella	*Veillonella dispar/tubetsuensis*	99.38
ASV_126 (*Veillonella* spp.)					Bacteria__Firmicutes__Negativicutes__Selenomonadales__Veillonellaceae__Veillonella	*Veillonella dispar/tubetsuensis*	98.76
ASV_139 (*Streptococcus thermophilus*)					Bacteria__Firmicutes__Bacilli__Lactobacillales__Streptococcaceae__Streptococcus__salivarius	***Streptococcus thermophilus***	**100**
ASV_152 (*Streptococcus* spp.)					Bacteria__Firmicutes__Bacilli__Lactobacillales__Streptococcaceae__Streptococcus	*Streptococcus mitis* group	99.38
ASV_161 (*Actinomyces* spp.)					Bacteria__Actinobacteria__Actinobacteria__Actinomycetales__Actinomycetaceae__Actinomyces__odontolyticus	*Actinomyces turicensis*	97.39
ASV_165 (*Streptococcus parasanguinis*)					Bacteria__Firmicutes__Bacilli__Lactobacillales__Streptococcaceae__Streptococcus	***Streptococcus parasanguinis***	**100**
ASV_176 (*Actinomyces* spp.)					Bacteria__Actinobacteria__Actinobacteria__Actinomycetales__Actinomycetaceae__Actinomyces	*Actinomyces meyeri*	99.35
ASV_178 (*Lachnoanaerobaculum orale*)					Bacteria__Firmicutes__Clostridia__Clostridiales__Lachnospiraceae__Lachnoanaerobaculum	***Lachnoanaerobaculum orale***	**100**
ASV_181 (*Haemophilus parainfluenzae*)					Bacteria__Proteobacteria__Gammaproteobacteria__Pasteurellales__Pasteurellaceae__Haemophilus__parainfluenzae	***Haemophilus parainfluenzae***	**100**
ASV_182 (*Actinomyces* spp.)					Bacteria__Actinobacteria__Actinobacteria__Actinomycetales__Actinomycetaceae__Actinomyces	*Actinomyces turicensis*	96.73
ASV_188 (*Streptococcus* spp.)					Bacteria__Firmicutes__Bacilli__Lactobacillales__Streptococcaceae__Streptococcus	*Streptococcus sinensis*	99.38
ASV_191 (*Atopobium parvulum*)					Bacteria__Actinobacteria__Actinobacteria__Coriobacteriales__Coriobacteriaceae__Atopobium__parvulum	***Atopobium parvulum***	**100**
ASV_213 (*Gemella* spp.)					Bacteria__Firmicutes__Bacilli__Bacillales__Bacillales_Incertae_Sedis_XI__Gemella	*Gemella taiwanensis/haemolysans*	99.38
ASV_251 (*Veillonella rogosae*)					Bacteria__Firmicutes__Negativicutes__Selenomonadales__Veillonellaceae__Veillonella__rogosae	***Veillonella rogosae***	**100**
ASV_257 (*Actinomyces* spp.)					Bacteria__Actinobacteria__Actinobacteria__Actinomycetales__Actinomycetaceae__Actinomyces	*Actinomyces meyeri/odontolyticus*	97.37
ASV_378 (*Haemophilus* spp.)					Bacteria__Proteobacteria__Gammaproteobacteria__Pasteurellales__Pasteurellaceae__Haemophilus__sputorum	*Haemophilus sputorum*	99.38
ASV_535 (*Haemophilus* spp.)					Bacteria__Proteobacteria__Gammaproteobacteria__Pasteurellales__Pasteurellaceae__Haemophilus	*Haemophilus parainfluenzae*	98.75

[1]Only ASVs shared in more than 3 mother-infant pairs in either foremilk or hindmilk at infant age 5 or 9 months were included

[2]Species level classification only adopted if top nBLAST hit showed 100% homology and second hit showed <100% homology, as marked with bold text

Colorkey

	>3 mother-infant pairs
	3 mother infant pairs
	2 mother infant pairs
	1 monther infant pair
	Never shared

Table S6 - Genus level relative abundance in infant feces from infants of secretor vs non-secretor mothers at infant age 5 months				
**Genus[1]**	**Average relative abundance in feces of infant from non-secretor mothers (%), n=5**	**Average relative abundance in feces of infants from secretor mothers (%), n=24[2]**	**P-value[3]**	**FDR corrected P-value**
Bifidobacterium	81.131	65.201	0.518	0.754
Veillonella	0.494	2.759	0.204	0.683
Streptococcus	0.764	2.455	0.225	0.683
Enterococcus	2.258	4.644	0.419	0.701
Lactobacillus	0.440	1.296	0.578	0.760
Escherichia/Shigella	1.234	0.334	**0.030**	0.474
Bacteroides	6.767	11.513	0.363	0.701
Clostridium sensu stricto	0.137	0.422	0.299	0.683
Parabacteroides	1.132	3.794	0.282	0.683
Clostridiaceae uncl.	0.001	1.558	0.208	0.683
Blautia	0.121	1.113	0.665	0.760
Collinsella	2.120	1.041	0.438	0.701
Klebsiella	0.038	0.835	0.895	0.954
Lachnospiraceae uncl.	0.344	0.703	0.622	0.760
Anaerostipes	0.168	0.194	0.237	0.683
Clostridium_XVIII	0.056	0.114	0.967	0.967
[1]Only taxa with average relative abundance > 0.2% across all samples were included				
[2]One sample excluded due to maternal antibiotics during sampling				
[3]Statistical significance was evaluated by Mann Whitney tests				

Table S7 - ASV level relative abundance in infant feces from infants of secretor vs non-secretor mothers at infant age 5 months

ASV (assigned taxonomy)[1]	Average relative abundance in feces of infant from non-secretor mothers (%), n=5	Average relative abundance in feces of infants from secretor mothers (%), n=24[2]	P-value[3]	FDR corrected P-value	RDP taxonomy	nBLAST top hit against reference RNA sequence database at NCBI (% identity)[4]
ASV_40 (*Bifidobacterium* spp.)	0.001	2.637	0.701	0.871	Bacteria__Actinobacteria__Actinobacteria__Bifidobacteriales__Bifidobacteriaceae__Bifidobacterium	*B. breve (98.63%)*
ASV_37 (*Bifidobacterium catenulatum* group)	1.379	0.289	0.442	0.871	Bacteria__Actinobacteria__Actinobacteria__Bifidobacteriales__Bifidobacteriaceae__Bifidobacterium	***B. catenulatum group (100%)***
ASV_18 (*Bifidobacterium bifidum*)	0.936	2.191	0.573	0.871	Bacteria__Actinobacteria__Actinobacteria__Bifidobacteriales__Bifidobacteriaceae__Bifidobacterium__bifidum	***B. bifidum (100%)***
ASV_17 (*Bifidobacterium breve*)	0.187	3.448	0.327	0.871	Bacteria__Actinobacteria__Actinobacteria__Bifidobacteriales__Bifidobacteriaceae__Bifidobacterium__breve	***B. breve (100%)***
ASV_108 (*Bifidobacterium spp*)	0.001	0.489	1.000	1.000	Bacteria__Actinobacteria__Actinobacteria__Bifidobacteriales__Bifidobacteriaceae__Bifidobacterium__breve	*B. scardovii (99.30 %)*
ASV_1 (*Bifidobacterium longum*)	77.673	49.428	0.069	0.734	Bacteria__Actinobacteria__Actinobacteria__Bifidobacteriales__Bifidobacteriaceae__Bifidobacterium__longum	***B. longum (100%)***
ASV_7 (*Bifidobacterium longum*)	0.671	6.367	0.602	0.871	Bacteria__Actinobacteria__Actinobacteria__Bifidobacteriales__Bifidobacteriaceae__Bifidobacterium__longum	***B. longum (100%)***
ASV_26 (*Collinsella aerofaeciens*)	2.120	1.041	0.438	0.871	Bacteria__Actinobacteria__Actinobacteria__Coriobacteriales__Coriobacteriaceae__Collinsella__aerofaciens	***C. aerofaciens (100%)***
ASV_98 (*Bacteroides* spp.)	0.084	0.494	0.967	0.999	Bacteria__Bacteroidetes__Bacteroidia__Bacteroidales__Bacteroidaceae__Bacteroides	*B. xylanisolvens (99.35%)*
ASV_35 (*Bacteroides* spp.)	0.001	1.377	0.459	0.871	Bacteria__Bacteroidetes__Bacteroidia__Bacteroidales__Bacteroidaceae__Bacteroides	*B. dorei (98.71%)*
ASV_15 (*Bacteroides vulgatus*)	0.829	2.432	0.442	0.871	Bacteria__Bacteroidetes__Bacteroidia__Bacteroidales__Bacteroidaceae__Bacteroides	***B. vulgatus (100%)***
ASV_59 (*Bacteroides* spp.)	0.014	3.037	0.188	0.871	Bacteria__Bacteroidetes__Bacteroidia__Bacteroidales__Bacteroidaceae__Bacteroides	*B. caccae/faecium (99.35%)*
ASV_86 (*Bacteroides fragilis*)	2.000	0.129	0.096	0.766	Bacteria__Bacteroidetes__Bacteroidia__Bacteroidales__Bacteroidaceae__Bacteroides__fragilis	***B. fragilis (100%)***
ASV_61 (*Bacteroides thetaiotaomicron*)	0.317	0.396	0.665	0.871	Bacteria__Bacteroidetes__Bacteroidia__Bacteroidales__Bacteroidaceae__Bacteroides__thetaiotaomicron	***B. thetaiotaomicron (100%)***
ASV_70 (*Bacteroides xylanisolvens*)	0.001	0.396	0.870	0.960	Bacteria__Bacteroidetes__Bacteroidia__Bacteroidales__Bacteroidaceae__Bacteroides__xylanisolvens	***B. xylanisolvens (100%)***
ASV_71 (*Parabacteroides distasonis*)	0.007	1.102	0.665	0.871	Bacteria__Bacteroidetes__Bacteroidia__Bacteroidales__Porphyromonadaceae__Parabacteroides__distasonis	***P. diastonis (100%)***
ASV_39 (*Enterococcus faecalis*)	1.208	1.924	0.707	0.871	Bacteria__Firmicutes__Bacilli__Lactobacillales__Enterococcaceae__Enterococcus	***E. faecalis (100%)***
ASV_36 (*Enterococcus faecium* group)	1.012	2.558	0.498	0.871	Bacteria__Firmicutes__Bacilli__Lactobacillales__Enterococcaceae__Enterococcus	***E. faecium group (100%)***
ASV_46 (*Lactobacillus casei* group)	0.384	1.110	0.423	0.871	Bacteria__Firmicutes__Bacilli__Lactobacillales__Lactobacillaceae__Lactobacillus	***L. casei group (100%)***
ASV_12 (*Streptococcus* spp.)	0.132	0.488	0.298	0.871	Bacteria__Firmicutes__Bacilli__Lactobacillales__Streptococcaceae__Streptococcus	*S. perorsis/lactarius (99.38%)*
ASV_2 (*Streptococcus mitis* group)	0.066	0.667	**0.035**	0.560	Bacteria__Firmicutes__Bacilli__Lactobacillales__Streptococcaceae__Streptococcus	***S. mitis group (100%)***
ASV_6 (*Streptococcus salivarius*)	0.418	0.926	0.707	0.871	Bacteria__Firmicutes__Bacilli__Lactobacillales__Streptococcaceae__Streptococcus	***S. salivarius (100%)***
ASV_50 (*Clostridiaceae* spp.)	0.001	1.408	0.275	0.871	Bacteria__Firmicutes__Clostridia__Clostridiales__Clostridiaceae_1	*D. massiliensis (98.52%)*
ASV_186 (*Clostridium perfringens*)	0.107	0.255	0.622	0.871	Bacteria__Firmicutes__Clostridia__Clostridiales__Clostridiaceae_1__Clostridium_sensu_stricto	***C. perfringens (100%)***
ASV_34 (*Ruminococcus gnavus*)	0.299	0.620	0.442	0.871	Bacteria__Firmicutes__Clostridia__Clostridiales__Lachnospiraceae	***R. gnavus (100%)***
ASV_73 (*ClostridiumXVIII ramosum*)	0.056	0.114	0.967	0.999	Bacteria__Firmicutes__Erysipelotrichia__Erysipelotrichales__Erysipelotrichaceae__Clostridium_XVIII__ramosum	***E. ramosum (100%)***
ASV_21 (*Veillonella* spp.)	0.322	1.229	0.663	0.871	Bacteria__Firmicutes__Negativicutes__Selenomonadales__Veillonellaceae__Veillonella	*V. dispar (99.83%)*
ASV_11 (*Veillonella dispar*)	0.103	0.637	0.225	0.871	Bacteria__Firmicutes__Negativicutes__Selenomonadales__Veillonellaceae__Veillonella	***V. dispar (100%)***
ASV_42 (*Veillonella atypica*)	0.019	0.311	0.349	0.871	Bacteria__Firmicutes__Negativicutes__Selenomonadales__Veillonellaceae__Veillonella	***V. atypica (100%)***
ASV_13 (*Escherichia/Shigella* spp.)	0.864	0.298	**0.033**	0.560	Bacteria__Proteobacteria__Gammaproteobacteria__Enterobacteriales__Enterobacteriaceae__Escherichia/Shigella	*Escherichia/Shigella spp. (100%)*
ASV_77 (*Klebsiella* spp.)	0.030	0.377	0.812	0.960	Bacteria__Proteobacteria__Gammaproteobacteria__Enterobacteriales__Enterobacteriaceae__Klebsiella	*Klebsiella spp. (100%)*
ASV_63 (*Klebsiella* spp.)	0.006	0.188	0.848	0.960	Bacteria__Proteobacteria__Gammaproteobacteria__Enterobacteriales__Enterobacteriaceae__Klebsiella	*Klebsiella spp. (99.38%)*

[1]Only ASVs with average relative abundance >0.2% and prevalence >15% across all samples were included

[2]One sample excluded due to maternal antibiotics during sampling

[3]Statistical significance was evaluated by Mann Whitney tests

[4]Species level classification only adopted if top nBLAST hit showed 100% homology and second hit showed <100% homology, as marked with bold text

Table S8 - Adonis tests assessing impact HMOs in breast milk on microbial community composition in infant feces at infant age 5 months (n=29#)												
**HMO category**	**Weighted Unifrac**			**Unweighted Unifrac**			**Bray Curtis**			**Jaccard**		
	**R^2**	**P-value**	**FDR-corrected P-value**	**R^2**	**P-value**	**FDR-corrected P-value**	**R^2**	**P-value**	**FDR-corrected P-value**	**R^2**	**P-value**	**FDR-corrected P-value**
Diversity index	0.026	0.474	0.758	0.024	0.861	0.977	0.031	0.525	0.871	0.032	0.703	0.839
2'FL (nmol/ml)	0.051	0.218	0.758	0.039	0.316	0.789	0.046	0.204	0.753	0.039	0.263	0.839
3FL (nmol/ml)	0.075	0.103	0.758	0.043	0.214	0.789	0.064	0.075	0.753	0.041	0.154	0.839
LNnT (nmol/ml)	0.023	0.524	0.758	0.026	0.778	0.964	0.020	0.861	0.898	0.034	0.571	0.839
3'SL (nmol/ml)	0.038	0.313	0.758	0.034	0.493	0.789	0.025	0.726	0.871	0.032	0.769	0.839
DFLac (nmol/ml)	0.036	0.352	0.758	0.028	0.719	0.959	0.041	0.307	0.819	0.032	0.698	0.839
6'SL (nmol/ml)	0.028	0.442	0.758	0.049	0.120	0.789	0.049	0.199	0.753	0.041	0.175	0.839
LNT (nmol/ml)	0.030	0.411	0.758	0.032	0.581	0.872	0.028	0.615	0.871	0.036	0.406	0.839
LNFP I (nmol/ml)	0.010	0.840	0.840	0.037	0.380	0.789	0.014	0.945	0.945	0.033	0.705	0.839
LNFP II (nmol/ml)	0.017	0.645	0.840	0.039	0.314	0.789	0.023	0.800	0.898	0.038	0.327	0.839
LNFP III (nmol/ml)	0.023	0.537	0.758	0.016	0.993	0.993	0.021	0.845	0.898	0.033	0.623	0.839
LSTb (nmol/ml)	0.025	0.482	0.758	0.046	0.161	0.789	0.030	0.519	0.871	0.036	0.447	0.839
LSTc (nmol/ml)	0.011	0.805	0.840	0.037	0.450	0.789	0.029	0.567	0.871	0.047	**0.038**	0.456
DFLNT (nmol/ml)	0.013	0.746	0.840	0.026	0.803	0.964	0.025	0.718	0.871	0.032	0.747	0.839
LNH (nmol/ml)	0.010	0.802	0.840	0.021	0.921	0.977	0.024	0.720	0.871	0.031	0.819	0.855
DSLNT (nmol/ml)	0.054	0.198	0.758	0.038	0.353	0.789	0.043	0.241	0.753	0.029	0.901	0.901
FLNH (nmol/ml)	0.025	0.472	0.758	0.021	0.936	0.977	0.030	0.538	0.871	0.033	0.710	0.839
DFLNH (nmol/ml)	0.009	0.819	0.840	0.046	0.159	0.789	0.022	0.700	0.871	0.035	0.522	0.839
FDSLNH (nmol/ml)	0.014	0.694	0.840	0.039	0.337	0.789	0.026	0.656	0.871	0.049	**0.014**	0.336
DSLNH (nmol/ml)	0.033	0.336	0.758	0.031	0.658	0.929	0.049	0.251	0.753	0.039	0.240	0.839
Total HMOs (nmol/ml)	0.047	0.236	0.758	0.037	0.400	0.789	0.045	0.202	0.753	0.036	0.401	0.839
Sialylated HMOs (nmol/ml)	0.030	0.403	0.758	0.045	0.181	0.789	0.029	0.608	0.871	0.040	0.186	0.839
Fucosylated HMOs (nmol/ml)	0.043	0.294	0.758	0.035	0.475	0.789	0.043	0.230	0.753	0.036	0.450	0.839

#One sample excluded due to maternal antibiotics during sampling

Table S9 - Correlations between HMOs in breast milk and relative abundance of genus level taxa in infant feces at infant age 5 months (n=29[#])

Correlation coefficient

Genus[1]	Diversity index	2'FL (nmol/ml)	3FL (nmol/ml)	LNnT (nmol/ml)	3'SL (nmol/ml)	DFLac (nmol/ml)	6'SL (nmol/ml)	LNT (nmol/ml)	LNFP I (nmol/ml)	LNFP II (nmol/ml)	LNFP III (nmol/ml)	LSTb (nmol/ml)	LSTc (nmol/ml)	DFLNT (nmol/ml)	LNH (nmol/ml)	DSLNT (nmol/ml)	FLNH (nmol/ml)	DFLNH (nmol/ml)	FDSLNH (nmol/ml)	DSLNH (nmol/ml)	Total HMOs (nmol/ml)	Sialylated HMOs (nmol/ml)	Fucosylated HMOs (nmol/ml)
Enterococcus	-0.2261	0.3020	0.2562	-0.2234	0.1909	0.1485	-0.0323	-0.3321	0.1126	-0.2976	-0.0259	-0.3813	0.2473	-0.1630	-0.0399	-0.3284	-0.0643	0.2340	-0.0692	-0.0015	0.2399	-0.1387	0.2739
Clostridiaceae uncl.	-0.0866	0.2250	0.2283	-0.0331	0.1991	0.2415	-0.1086	-0.2338	0.0976	-0.1704	-0.1197	-0.0733	0.2404	0.1464	-0.0248	-0.1246	-0.1340	-0.3446	-0.2007	-0.0728	0.2156	-0.0778	0.2233
Streptococcus	-0.1315	0.1638	0.1806	-0.1663	0.1345	0.3160	-0.2099	0.0140	0.0618	-0.1742	0.0143	-0.0185	-0.0616	0.2222	-0.0119	0.1742	0.1104	-0.2461	-0.3200	-0.1478	0.2249	-0.0298	0.2094
Clostridium sensu stricto	-0.4781	0.2232	0.0846	-0.1685	-0.0228	-0.0426	0.0646	-0.1936	-0.0907	-0.0066	-0.1297	0.0124	0.1153	-0.3378	-0.1645	-0.1221	-0.2065	0.1703	0.1178	0.2673	0.2278	-0.0545	0.1984
Clostridium_XVIII	0.2156	-0.1656	-0.0376	0.0334	0.2657	0.0828	0.1266	0.0209	-0.1489	0.2184	-0.0035	0.1301	-0.0675	0.4369	0.3945	0.0897	0.3742	-0.4090	0.3179	0.1106	-0.1830	0.3346	-0.0765
Veillonella	-0.0618	0.1872	-0.0850	-0.1747	-0.0150	0.1020	-0.1187	0.0384	0.2878	-0.3153	-0.4634	-0.1013	-0.1954	0.3228	0.0274	-0.1540	0.0828	0.1057	-0.3235	-0.3671	0.1732	-0.3631	0.2092
Blautia	0.0164	-0.0830	-0.0986	0.0107	0.1142	-0.0591	-0.0797	0.0871	-0.0772	0.0435	0.0337	0.1709	-0.0353	0.0649	-0.0748	0.1093	0.0895	-0.1684	-0.0238	-0.2013	-0.0427	0.0468	-0.0427
Bacteroides	0.3302	-0.2837	-0.1051	0.0260	0.0400	0.0135	0.1186	0.0816	-0.2427	0.3987	0.2977	0.3792	-0.2527	-0.0951	0.0615	0.2376	-0.0886	-0.0110	0.3152	0.0861	-0.2246	0.4178	-0.2216
Bifidobacterium	-0.0591	-0.1039	-0.1626	0.2680	-0.3276	-0.2808	0.1857	0.1828	0.0759	-0.0502	-0.0749	0.0833	0.0552	-0.0182	-0.1345	0.3133	-0.0010	0.0325	-0.1207	0.0517	-0.1310	-0.1300	-0.1970
Lachnospiraceae uncl.	0.3698	-0.3088	-0.1769	-0.0925	0.1634	-0.0886	0.0664	0.0198	-0.2181	0.2136	-0.0727	0.0553	-0.1754	0.2013	0.2608	-0.1000	0.2764	0.0072	0.3608	0.0991	-0.3200	0.2563	-0.2325
Klebsiella	-0.1073	0.0212	-0.1883	-0.1429	-0.3052	-0.1993	-0.0839	0.3230	0.2329	-0.2016	-0.0037	0.1169	-0.3168	-0.0583	-0.2780	0.3394	0.0957	0.3478	-0.3515	-0.3995	0.0426	-0.2112	-0.0418
Anaerostipes	0.2282	-0.2623	-0.2219	0.0452	-0.0104	-0.1864	0.1030	0.0946	-0.2045	0.2247	0.1468	0.2588	-0.0438	0.0529	0.1823	0.1663	0.2769	-0.0654	0.3443	0.0480	-0.2713	0.2261	-0.2490
Lactobacillus	0.1830	-0.1938	-0.2451	-0.0763	0.1433	-0.0418	0.3198	0.0803	-0.1888	0.1225	-0.0453	-0.1098	-0.1158	0.2646	0.2648	-0.3001	0.3973	0.0148	0.3238	0.3113	-0.2188	0.1420	-0.0645
Parabacteroides	0.1260	-0.1604	-0.2857	0.2277	-0.0889	-0.2145	0.2368	0.1643	-0.0561	0.1351	-0.0203	0.1657	-0.0030	0.0597	-0.0249	0.1020	0.0226	-0.1306	0.1643	0.0400	-0.1670	0.1266	-0.1516
Collinsella	0.2745	-0.2819	-0.3156	0.1893	0.0659	-0.1590	0.3596	0.3296	-0.1463	0.1835	0.1262	0.3056	-0.1281	0.3925	0.1980	0.1995	0.2883	-0.2778	0.1607	0.1375	-0.3506	0.3062	-0.2896
Escherichia/Shigella	0.2920	-0.4595	-0.4429	0.3954	-0.3489	-0.3474	0.5758	0.4699	-0.2623	0.2596	0.0829	0.4125	-0.0836	-0.0981	-0.1330	0.3375	-0.0280	0.1111	0.0678	0.3566	-0.4454	0.0990	-0.5090

P-values[2]

	Diversity index	2'FL (nmol/ml)	3FL (nmol/ml)	LNnT (nmol/ml)	3'SL (nmol/ml)	DFLac (nmol/ml)	6'SL (nmol/ml)	LNT (nmol/ml)	LNFP I (nmol/ml)	LNFP II (nmol/ml)	LNFP III (nmol/ml)	LSTb (nmol/ml)	LSTc (nmol/ml)	DFLNT (nmol/ml)	LNH (nmol/ml)	DSLNT (nmol/ml)	FLNH (nmol/ml)	DFLNH (nmol/ml)	FDSLNH (nmol/ml)	DSLNH (nmol/ml)	Total HMOs (nmol/ml)	Sialylated HMOs (nmol/ml)	Fucosylated HMOs (nmol/ml)
Enterococcus	0.2382	0.1113	0.1798	0.2440	0.3212	0.4419	0.8680	0.0784	0.5610	0.1169	0.8940	0.0412	0.1958	0.3983	0.8371	0.0820	0.7404	0.2218	0.7212	0.9939	0.2100	0.4731	0.1505
Clostridiaceae uncl.	0.6552	0.2406	0.2336	0.8647	0.3005	0.2069	0.5748	0.2222	0.6145	0.3768	0.5364	0.7054	0.2090	0.4485	0.8983	0.5195	0.4883	0.0671	0.2965	0.7075	0.2613	0.6885	0.2442
Streptococcus	0.4964	0.3958	0.3486	0.3887	0.4867	0.0949	0.2745	0.9424	0.7500	0.3662	0.9414	0.9242	0.7510	0.2466	0.9509	0.3662	0.5687	0.1982	0.0906	0.4442	0.2408	0.8780	0.2757
Clostridium sensu stricto	0.0087	0.2445	0.6625	0.3823	0.9065	0.8265	0.7392	0.3144	0.6398	0.9729	0.5024	0.9490	0.5515	0.0731	0.3940	0.5280	0.2825	0.3772	0.5427	0.1610	0.2347	0.7790	0.3022
Clostridium_XVIII	0.2612	0.3907	0.8466	0.8635	0.1635	0.6694	0.5128	0.9144	0.4409	0.2550	0.9857	0.5012	0.7280	0.0178	0.0342	0.6434	0.0455	0.0276	0.0928	0.5679	0.3421	0.0760	0.6932
Veillonella	0.7500	0.3308	0.6611	0.3648	0.9383	0.5986	0.5395	0.8431	0.1301	0.0956	0.0113	0.6012	0.3098	0.0877	0.8880	0.4252	0.6694	0.5853	0.0869	0.0501	0.3689	0.0528	0.2762
Blautia	0.9326	0.6687	0.6109	0.9562	0.5553	0.7605	0.6812	0.6533	0.6905	0.8226	0.8623	0.3755	0.8557	0.7380	0.6999	0.5726	0.6441	0.3825	0.9024	0.2951	0.8259	0.8094	0.8259
Bacteroides	0.0802	0.1359	0.5875	0.8934	0.8367	0.9446	0.5401	0.6741	0.2047	0.0321	0.1168	0.0425	0.1861	0.6237	0.7511	0.2145	0.6478	0.9548	0.0958	0.6571	0.2414	0.0241	0.2479
Bifidobacterium	0.7607	0.5916	0.3995	0.1599	0.0828	0.1401	0.3348	0.3427	0.6957	0.7958	0.6995	0.6677	0.7762	0.9252	0.4867	0.0979	0.9960	0.8670	0.5329	0.7899	0.4981	0.5013	0.3056
Lachnospiraceae uncl.	0.0483	0.1031	0.3585	0.6331	0.3969	0.6475	0.7322	0.9187	0.2557	0.2659	0.7078	0.7758	0.3626	0.2950	0.1718	0.6056	0.1467	0.9704	0.0545	0.6089	0.0906	0.1796	0.2248
Klebsiella	0.5796	0.9132	0.3279	0.4597	0.1074	0.2999	0.6654	0.0875	0.2240	0.2944	0.9849	0.5460	0.0941	0.7638	0.1443	0.0717	0.6214	0.0645	0.0615	0.0318	0.8262	0.2715	0.8296
Anaerostipes	0.2339	0.1693	0.2473	0.8158	0.9572	0.3329	0.5951	0.6254	0.2872	0.2413	0.4474	0.1753	0.8214	0.7853	0.3440	0.3887	0.1460	0.7361	0.0674	0.8047	0.1546	0.2383	0.1927
Lactobacillus	0.3419	0.3138	0.2001	0.6942	0.4584	0.8297	0.0908	0.6789	0.3267	0.5266	0.8157	0.5708	0.5498	0.1654	0.1650	0.1138	0.0328	0.9395	0.0866	0.1002	0.2541	0.4624	0.7395
Parabacteroides	0.5150	0.4058	0.1330	0.2350	0.6465	0.2638	0.2161	0.3943	0.7726	0.4846	0.9166	0.3905	0.9879	0.7583	0.8978	0.5985	0.9072	0.4997	0.3943	0.8367	0.3867	0.5128	0.4326
Collinsella	0.1496	0.1384	0.0954	0.3253	0.7343	0.4100	0.0553	0.0808	0.4488	0.3406	0.5141	0.1069	0.5077	0.0352	0.3031	0.2994	0.1294	0.1445	0.4051	0.4768	0.0623	0.1062	0.1275
Escherichia/Shigella	0.1243	0.0122	0.0161	0.0338	0.0636	0.0648	0.0011	0.0101	0.1693	0.1739	0.6690	0.0262	0.6662	0.6126	0.4915	0.0734	0.8855	0.5661	0.7268	0.0576	0.0155	0.6095	0.0048

FDR corrected P-values[3]

	Diversity index	2'FL (nmol/ml)	3FL (nmol/ml)	LNnT (nmol/ml)	3'SL (nmol/ml)	DFLac (nmol/ml)	6'SL (nmol/ml)	LNT (nmol/ml)	LNFP I (nmol/ml)	LNFP II (nmol/ml)	LNFP III (nmol/ml)	LSTb (nmol/ml)	LSTc (nmol/ml)	DFLNT (nmol/ml)	LNH (nmol/ml)	DSLNT (nmol/ml)	FLNH (nmol/ml)	DFLNH (nmol/ml)	FDSLNH (nmol/ml)	DSLNH (nmol/ml)	Total HMOs (nmol/ml)	Sialylated HMOs (nmol/ml)	Fucosylated HMOs (nmol/ml)
Enterococcus	0.5225	0.4429	0.5653	0.7773	0.8565	0.7856	0.8680	0.3498	0.7726	0.5481	0.9857	0.2265	0.8360	0.7966	0.9509	0.3640	0.9112	0.5914	0.7752	0.9939	0.4181	0.7459	0.4445
Clostridiaceae uncl.	0.8064	0.4889	0.5653	0.9530	0.8565	0.7609	0.7884	0.7110	0.7726	0.5481	0.9857	0.8681	0.8360	0.7973	0.9509	0.6434	0.8926	0.3580	0.5270	0.8925	0.4181	0.8474	0.4445
Streptococcus	0.7491	0.4995	0.5736	0.7773	0.8652	0.7472	0.7884	0.9424	0.7726	0.5481	0.9857	0.9490	0.9127	0.6576	0.9509	0.6434	0.8926	0.5914	0.1916	0.8925	0.4181	0.8780	0.4445
Clostridium sensu stricto	0.1394	0.4889	0.7067	0.7773	0.9572	0.8850	0.7884	0.7832	0.7726	0.9729	0.9857	0.9490	0.9127	0.3507	0.8739	0.6434	0.7534	0.7651	0.6680	0.5151	0.4181	0.8634	0.4445
Clostridium_XVIII	0.5225	0.4995	0.8466	0.9530	0.6542	0.8850	0.7884	0.9424	0.7726	0.5481	0.9857	0.8681	0.9127	0.2816	0.5474	0.6434	0.3639	0.3580	0.1916	0.8925	0.4759	0.4055	0.8296
Veillonella	0.8114	0.4995	0.7067	0.7773	0.9572	0.8850	0.7884	0.9424	0.7467	0.5481	0.1816	0.8681	0.9127	0.3507	0.9509	0.6434	0.8926	0.8513	0.1916	0.3073	0.4759	0.4055	0.4445
Blautia	0.9326	0.7133	0.7067	0.9562	0.8885	0.8850	0.7884	0.9052	0.7726	0.8774	0.9857	0.8681	0.9127	0.8377	0.9509	0.6434	0.8926	0.7651	0.9024	0.7871	0.8262	0.8634	0.8296
Bacteroides	0.4278	0.4429	0.7067	0.9530	0.9572	0.9446	0.7884	0.9052	0.7467	0.5143	0.9344	0.2265	0.8360	0.8377	0.9509	0.5719	0.8926	0.9704	0.1916	0.8925	0.4181	0.3861	0.4445
Bifidobacterium	0.8114	0.6761	0.5811	0.7773	0.5729	0.7472	0.7884	0.7832	0.7726	0.8774	0.9857	0.8681	0.9127	0.9252	0.8739	0.3640	0.9960	0.9704	0.6680	0.8925	0.5692	0.7459	0.4445
Lachnospiraceae uncl.	0.3865	0.4429	0.5736	0.9530	0.8652	0.8850	0.7884	0.9424	0.7467	0.5481	0.9857	0.8866	0.9127	0.6743	0.6872	0.6434	0.4694	0.9704	0.1916	0.8925	0.4181	0.5749	0.4445
Klebsiella	0.7728	0.9132	0.5736	0.8173	0.5729	0.7609	0.7884	0.3498	0.7467	0.5481	0.9857	0.8681	0.8360	0.8377	0.6872	0.3640	0.8926	0.3580	0.1916	0.3073	0.8262	0.6205	0.8296
Anaerostipes	0.5225	0.4516	0.5653	0.9530	0.9572	0.7609	0.7884	0.9052	0.7467	0.5481	0.9857	0.5609	0.9127	0.8377	0.8739	0.6434	0.4694	0.9704	0.1916	0.8925	0.4181	0.6205	0.4445
Lactobacillus	0.6078	0.4995	0.5653	0.9530	0.8652	0.8850	0.4841	0.9052	0.7467	0.6481	0.9857	0.8681	0.9127	0.5293	0.6872	0.3640	0.3639	0.9704	0.1916	0.4008	0.4181	0.7459	0.8296
Parabacteroides	0.7491	0.4995	0.5653	0.7773	0.9404	0.7609	0.7884	0.7885	0.7726	0.6461	0.9857	0.8681	0.9879	0.8377	0.9509	0.6434	0.9677	0.8513	0.5892	0.8925	0.4759	0.7459	0.5768
Collinsella	0.4787	0.4429	0.5653	0.7773	0.9572	0.7856	0.4427	0.3498	0.7726	0.5481	0.9857	0.4276	0.9127	0.2816	0.8739	0.6434	0.4694	0.5782	0.5892	0.8925	0.4181	0.4249	0.4445
Escherichia/Shigella	0.4787	0.1945	0.2579	0.5400	0.5729	0.7472	0.0173	0.1618	0.7467	0.5481	0.9857	0.2265	0.9127	0.8377	0.8739	0.3640	0.9677	0.8513	0.7752	0.3073	0.2475	0.8127	0.0769

[#]One sample excluded due to maternal antibiotics during sampling

[1]Only taxa with average relative abundance > 0.2% across all samples were included

[2]P-values below 0.05 are highlighted

[3]FDR corrected P-values below 0.1 are highlighted

Table S10 - Correlations between HMOs in breast milk and relative abundance of ASV level taxa in infant feces at infant age 5 months (n=29[#])

Correlation coefficient

ASV (Assigned taxonomy)[1]	Diversity index	2'FL (nmol/ml)	3FL (nmol/ml)	LNnT (nmol/ml)	3'SL (nmol/ml)	DFLac (nmol/ml)	6'SL (nmol/ml)	LNT (nmol/ml)	LNFP I (nmol/ml)	LNFP II (nmol/ml)	LNFP III (nmol/ml)	LSTb (nmol/ml)	LSTc (nmol/ml)	DFLNT (nmol/ml)	LNH (nmol/ml)	DSLNT (nmol/ml)	FLNH (nmol/ml)	DFLNH (nmol/ml)	FDSLNH (nmol/ml)	DSLNH (nmol/ml)	Total HMOs (nmol/ml)	Sialylated HMOs (nmol/ml)	Fucosylated HMOs (nmol/ml)
ASV_1 (*Bifidobacterium longum*)	0.0271	-0.2671	-0.3376	0.3016	-0.3411	-0.4460	0.3667	0.2721	-0.1089	0.1301	-0.0813	0.1671	0.0695	-0.0394	0.0032	0.2814	0.0798	0.1089	0.1410	0.3706	-0.2982	0.0453	-0.3351
ASV_108 (*Bifidobacterium* spp.)	-0.2002	0.1093	0.1242	-0.0483	0.2066	0.1766	-0.0225	-0.0999	-0.1138	-0.0202	-0.2417	-0.0434	-0.0666	-0.0614	-0.1183	-0.1811	-0.2705	-0.0318	-0.0277	0.0427	0.1407	-0.0741	0.1343
ASV_11 (*Veillonella dispar*)	0.0686	0.1236	0.1362	-0.2768	0.2240	0.2770	-0.2334	-0.1505	0.1110	-0.1715	-0.2299	-0.1347	-0.1320	0.1874	0.1151	-0.2852	0.0464	0.0891	-0.1152	-0.2509	0.1275	-0.1214	0.1919
ASV_12 (*Streptococcus* spp.)	-0.0308	0.0988	0.2541	-0.2068	0.1526	0.2314	-0.1316	-0.0510	-0.0106	-0.1720	0.0481	-0.1279	-0.0035	0.1272	-0.0118	0.1188	0.2437	-0.1831	-0.2033	-0.0601	0.1434	0.0596	0.1067
ASV_13 (*Escherichia/Shigella* spp.)	0.3271	-0.4717	-0.4538	0.4110	-0.3719	-0.3540	0.5997	0.4904	-0.2556	0.2646	0.1095	0.4518	-0.1167	-0.0868	-0.1373	0.3530	-0.0072	0.1148	0.0961	0.3443	-0.4665	0.1187	-0.5300
ASV_15 (*Bacteroides vulgatus*)	0.2383	-0.2842	-0.1748	-0.0944	-0.0216	0.1388	-0.0388	0.1851	-0.2467	0.3562	0.4694	0.3734	-0.4193	-0.0985	0.0003	0.2326	0.1291	-0.0522	0.3299	0.0478	-0.2455	0.3693	-0.1939
ASV_17 (*Bifidobacterium breve*)	0.1288	-0.0103	0.3223	-0.1091	0.1817	0.1323	-0.0370	-0.1720	0.0113	-0.1674	0.0748	-0.2883	0.2767	0.0899	-0.0165	-0.0197	0.1801	-0.0759	-0.0961	0.1058	0.0003	0.0545	0.0022
ASV_18 (*Bifidobacterium bifidum*)	0.1120	-0.0025	0.0938	0.2129	0.0527	0.0309	0.0294	-0.1064	0.1419	-0.1155	-0.1145	-0.1551	0.2154	0.1062	0.1029	-0.0456	-0.1054	-0.0532	-0.0710	-0.0375	-0.0613	-0.0633	-0.0867
ASV_186 (*Clostridium perfringens*)	-0.3136	0.0916	0.0433	-0.1763	-0.0378	0.1442	-0.0562	0.0120	-0.1207	0.0607	-0.0147	0.0742	-0.1754	-0.2217	-0.1245	0.0144	-0.1658	0.1871	0.1072	0.1793	0.1207	-0.0132	0.1024
ASV_2 (*Streptococcus mitis* group)	-0.2509	0.3571	0.3125	-0.2627	0.0922	0.3295	-0.3310	-0.1397	0.2745	-0.3738	-0.0614	-0.2585	0.0747	0.1351	0.0398	-0.0416	0.2597	0.0062	-0.3172	-0.2132	0.3898	-0.2181	0.3746
ASV_21 (*Veillonella* spp.)	-0.0129	0.0878	-0.0467	-0.1618	-0.1037	-0.1092	0.0948	0.0710	0.1921	-0.2784	-0.4283	-0.1901	-0.1285	0.0630	-0.1276	-0.2109	0.0417	0.2878	-0.2432	-0.1826	0.0690	-0.3380	0.0531
ASV_26 (*Collinsella aerofaeciens*)	0.2745	-0.2819	-0.3156	0.1893	0.0659	-0.1590	0.3596	0.3296	-0.1463	0.1835	0.1262	0.3056	-0.1281	0.3925	0.1980	0.1995	0.2883	-0.2778	0.1607	0.1375	-0.3506	0.3062	-0.2896
ASV_34 (*Ruminococcus gnavus*)	0.4184	-0.3662	-0.1204	-0.0191	0.1739	-0.0538	0.1029	-0.0528	-0.2964	0.2911	-0.0400	0.0550	-0.0894	0.0894	0.2790	-0.1254	0.2011	0.0009	0.4525	0.2089	-0.3602	0.3136	-0.2764
ASV_35 (*Bacteroides* spp.)	0.0679	0.0694	0.0472	0.1091	0.2765	0.2426	0.1232	-0.0682	-0.0944	0.1208	-0.0268	-0.0941	0.2062	0.3725	0.4104	-0.2128	0.2272	-0.4124	0.2696	0.2940	0.0676	0.1623	0.1936
ASV_36 (*Enterococcus faecium* group)	-0.1673	0.2334	0.1549	-0.3288	0.2841	0.2907	0.0041	-0.2825	0.0320	-0.1320	-0.2282	-0.2885	0.1406	-0.0482	0.0648	-0.0893	-0.1102	0.0733	-0.0050	0.0796	0.2152	0.0309	0.2987
ASV_37 (*Bifidobacterium catenulatum* group)	0.0410	-0.1038	-0.3127	0.2101	-0.0816	-0.1804	0.1923	0.1504	0.0047	0.0350	-0.1482	0.1714	0.0810	0.0638	0.0016	0.2567	0.1723	-0.0560	0.0313	-0.0219	-0.0447	0.0582	-0.1044
ASV_39 (*Enterococcus faecalis*)	-0.0903	0.1413	0.0340	-0.0767	-0.0930	-0.1571	0.0624	-0.0893	0.2180	-0.3081	-0.0999	-0.1741	0.0466	-0.0823	-0.1421	-0.1374	-0.0187	0.3411	-0.1401	-0.1472	0.0449	-0.2442	0.0168
ASV_40 (*Bifidobacterium* spp.)	0.0656	-0.0335	0.3002	-0.2933	0.4101	0.3330	-0.0390	-0.2487	-0.2717	0.1116	0.2602	-0.2080	0.0804	0.0497	0.0817	-0.0892	0.1621	-0.2277	0.1890	0.2743	0.0157	0.3593	0.0738
ASV_42 (*Veillonella atypica*)	-0.1053	0.1577	-0.0342	0.1224	-0.0761	0.0287	-0.0485	-0.0551	0.2079	-0.2928	-0.2917	-0.1847	0.3397	0.3259	0.1015	-0.0722	0.1202	-0.1577	-0.3623	-0.0303	0.1610	-0.3242	0.1858
ASV_46 (*Lactobacillus casei* group)	0.2548	-0.2735	-0.3444	-0.0172	0.0633	-0.0998	0.4052	0.1864	-0.2295	0.1975	0.0106	0.0213	-0.1783	0.2477	0.2541	-0.1697	0.4300	0.0213	0.3839	0.3303	-0.3014	0.1981	-0.1611
ASV_50 (*Clostridium* spp.)	-0.0472	0.1861	0.2503	-0.1042	0.2299	0.2390	-0.1839	-0.2839	0.0607	-0.1350	-0.0486	-0.0624	0.1828	0.0973	-0.0483	-0.1350	-0.1760	-0.2469	-0.1432	-0.0983	0.1760	-0.0376	0.2065
ASV_59 (*Bacteroides* spp.)	0.2712	-0.3008	-0.3969	0.2970	-0.2069	-0.2480	0.1852	0.3917	-0.0550	0.2144	0.1137	0.4556	-0.2428	0.0501	-0.1287	0.4003	-0.2656	0.0771	0.0168	-0.0991	-0.2794	0.1519	-0.3311
ASV_6 (*Streptococcus salivarius*)	0.0374	-0.0515	-0.0411	0.0118	0.0803	0.1904	-0.0079	0.1840	-0.0636	0.0133	0.0645	0.1922	-0.1404	0.2969	0.0678	0.2156	0.0668	-0.2993	-0.1838	-0.0182	0.0064	0.0621	0.0101
ASV_61 (*Bacteroides thetaiotaomicron*)	0.2834	-0.2144	-0.0370	-0.0403	0.2119	0.1314	-0.0123	-0.0657	-0.2456	0.3015	0.1043	0.2045	-0.3138	0.1241	0.1462	0.0879	-0.1725	-0.1947	0.2982	0.0863	-0.2727	0.4066	-0.1528
ASV_63 (*Klebsiella* spp.)	-0.1345	0.1007	-0.0882	-0.2139	-0.1686	-0.1404	-0.1588	0.1903	0.1663	-0.1775	-0.0430	-0.0636	-0.1630	-0.0523	-0.1553	0.1657	0.2441	0.2204	-0.1486	-0.2004	0.1210	-0.1312	0.0699
ASV_7 (*Bifidobacterium longum*)	0.1224	-0.0779	-0.1894	0.0922	0.0091	0.0705	0.0294	0.1988	0.0272	0.0655	0.1417	0.1709	-0.2853	0.3452	0.3066	-0.1098	0.3879	-0.0935	0.2302	-0.0964	-0.1160	0.0250	-0.0403
ASV_70 (*Bacteroides xylanisolvens*)	0.5271	-0.3235	-0.0358	0.0731	0.1806	0.1416	-0.0578	0.0929	-0.2272	0.3723	0.2439	0.2456	-0.3065	0.3022	0.2996	0.0324	0.0918	-0.2734	0.3896	0.0560	-0.3434	0.4025	-0.2564
ASV_71 (*Parabacteroides distasonis*)	-0.0296	-0.0386	-0.1700	0.1314	-0.1150	-0.1355	0.1651	0.0674	0.0534	0.0058	-0.1035	-0.0608	0.0969	-0.0263	-0.0370	0.0189	0.1208	-0.0304	0.0411	0.0049	0.0058	-0.0140	-0.0238
ASV_73 (*ClostridiumXVIII ramosum*)	0.2156	-0.1656	-0.0376	0.0334	0.2657	0.0828	0.1266	0.0209	-0.1489	0.2184	-0.0035	0.1301	-0.0675	0.4369	0.3945	0.0897	0.3742	-0.4090	0.3179	0.1106	-0.1830	0.3346	-0.0765
ASV_77 (*Klebsiella* spp.)	-0.1147	0.0215	-0.1615	-0.1200	-0.3425	-0.2582	-0.0566	0.3346	0.2333	-0.2132	0.0320	0.0607	-0.2527	-0.1389	-0.3158	0.2922	0.0866	0.4102	-0.3329	-0.3506	0.0442	-0.2306	-0.0697
ASV_86 (*Bacteroides fragilis*)	0.1814	-0.1751	-0.1474	-0.0426	0.0307	-0.0924	0.3558	0.0430	-0.3041	0.2357	0.0602	0.2110	-0.0969	-0.1483	-0.1594	0.2937	-0.1837	-0.1639	0.0973	0.2170	-0.2159	0.3146	-0.1960
ASV_98 (*Bacteroides* spp.)	0.3916	-0.2316	0.0014	0.0807	0.0223	0.0264	-0.0953	0.0424	-0.1816	0.2880	0.3972	0.2636	-0.2428	0.0762	0.1211	-0.0522	0.0403	-0.1336	0.3256	-0.0056	-0.2351	0.2831	-0.2136

P-value[2]

ASV	Diversity index	2'FL (nmol/ml)	3FL (nmol/ml)	LNnT (nmol/ml)	3'SL (nmol/ml)	DFLac (nmol/ml)	6'SL (nmol/ml)	LNT (nmol/ml)	LNFP I (nmol/ml)	LNFP II (nmol/ml)	LNFP III (nmol/ml)	LSTb (nmol/ml)	LSTc (nmol/ml)	DFLNT (nmol/ml)	LNH (nmol/ml)	DSLNT (nmol/ml)	FLNH (nmol/ml)	DFLNH (nmol/ml)	FDSLNH (nmol/ml)	DSLNH (nmol/ml)	Total HMOs (nmol/ml)	Sialylated HMOs (nmol/ml)	Fucosylated HMOs (nmol/ml)
ASV_1 (*Bifidobacterium longum*)	0.8890	0.1613	0.0733	0.1118	0.0702	0.0153	0.0504	0.1534	0.5738	0.5011	0.6750	0.3863	0.7202	0.8391	0.9868	0.1392	0.6806	0.5738	0.4658	0.0478	0.1162	0.8153	0.0755
ASV_108 (*Bifidobacterium* spp.)	0.2978	0.5726	0.5208	0.8036	0.2823	0.3594	0.9080	0.6061	0.5568	0.9171	0.2065	0.8231	0.7314	0.7518	0.5412	0.3471	0.1558	0.8699	0.8866	0.8261	0.4666	0.7025	0.4872
ASV_11 (*Veillonella dispar*)	0.7237	0.5230	0.4812	0.1461	0.2427	0.1457	0.2231	0.4359	0.5664	0.3738	0.2302	0.4860	0.4949	0.3304	0.5521	0.1337	0.8112	0.6459	0.5518	0.1893	0.5097	0.5305	0.3186
ASV_12 (*Streptococcus* spp.)	0.8740	0.6100	0.1835	0.2818	0.4295	0.2271	0.4962	0.7927	0.9565	0.3722	0.8045	0.5085	0.9858	0.5109	0.9514	0.5394	0.2026	0.3417	0.2901	0.7567	0.4579	0.7586	0.5816
ASV_13 (*Escherichia/Shigella* spp.)	0.0833	0.0098	0.0134	0.0268	0.0470	0.0596	0.0006	0.0069	0.1807	0.1654	0.5717	0.0139	0.5464	0.6545	0.4776	0.0604	0.9704	0.5533	0.6200	0.0674	0.0107	0.5396	0.0031
ASV_15 (*Bacteroides vulgatus*)	0.2132	0.1351	0.3645	0.6261	0.9115	0.4726	0.8417	0.3364	0.1970	0.0579	0.0102	0.0460	0.0236	0.6112	0.9987	0.2246	0.5043	0.7879	0.0805	0.8053	0.1993	0.0487	0.3136
ASV_17 (*Bifidobacterium breve*)	0.5056	0.9579	0.0882	0.5733	0.3456	0.4940	0.8489	0.3724	0.9535	0.3855	0.6999	0.1293	0.1462	0.6428	0.9324	0.9192	0.3500	0.6957	0.6200	0.5848	0.9989	0.7788	0.9911
ASV_18 (*Bifidobacterium bifidum*)	0.5630	0.9896	0.6286	0.2676	0.7860	0.8735	0.8797	0.5827	0.4628	0.5506	0.5541	0.4218	0.2618	0.5835	0.5953	0.8143	0.5863	0.7840	0.7146	0.8468	0.7520	0.7441	0.6549
ASV_186 (*Clostridium perfringens*)	0.0976	0.6365	0.8237	0.3602	0.8455	0.4556	0.7723	0.9507	0.5327	0.7545	0.9396	0.7021	0.3627	0.2478	0.5199	0.9408	0.3900	0.3310	0.5798	0.3520	0.5327	0.9458	0.5970
ASV_2 (*Streptococcus mitis* group)	0.1893	0.0572	0.0989	0.1686	0.6344	0.0809	0.0795	0.4697	0.1495	0.0458	0.7519	0.1757	0.7003	0.4848	0.8376	0.8302	0.1736	0.9747	0.0937	0.2669	0.0366	0.2557	0.0453
ASV_21 (*Veillonella* spp.)	0.9470	0.6505	0.8101	0.4018	0.5923	0.5729	0.6248	0.7145	0.3182	0.1436	0.0205	0.3233	0.5064	0.7453	0.5096	0.2721	0.8300	0.1300	0.2037	0.3430	0.7222	0.0729	0.7844
ASV_26 (*Collinsella aerofaeciens*)	0.1496	0.1384	0.0954	0.3253	0.7343	0.4100	0.0553	0.0808	0.4488	0.3406	0.5141	0.1069	0.5077	0.0352	0.3031	0.2994	0.1294	0.1445	0.4051	0.4768	0.0623	0.1062	0.1275
ASV_34 (*Ruminococcus gnavus*)	0.0239	0.0507	0.5339	0.9218	0.3671	0.7817	0.5954	0.7854	0.1184	0.1255	0.8367	0.7768	0.6446	0.6445	0.1428	0.5169	0.2956	0.9961	0.0137	0.2768	0.0549	0.0976	0.1466
ASV_35 (*Bacteroides* spp.)	0.7262	0.7204	0.8079	0.5731	0.1464	0.2048	0.5242	0.7251	0.6263	0.5324	0.8904	0.6274	0.2832	0.0466	0.0270	0.2677	0.2358	0.0262	0.1572	0.1216	0.7274	0.4002	0.3143
ASV_36 (*Enterococcus faecium* group)	0.3857	0.2230	0.4224	0.0816	0.1352	0.1260	0.9830	0.1376	0.8692	0.4949	0.2338	0.1290	0.4671	0.8038	0.7385	0.6451	0.5692	0.7055	0.9796	0.6813	0.2622	0.8737	0.1154
ASV_37 (*Bifidobacterium catenulatum* group)	0.8329	0.5920	0.0986	0.2739	0.6738	0.3490	0.3176	0.4361	0.9807	0.8569	0.4429	0.3741	0.6762	0.7423	0.9936	0.1788	0.3715	0.7730	0.8721	0.9103	0.8178	0.7644	0.5898
ASV_39 (*Enterococcus faecalis*)	0.6414	0.4646	0.8609	0.6925	0.6314	0.4157	0.7478	0.6451	0.2559	0.1040	0.6062	0.3663	0.8102	0.6714	0.4622	0.4773	0.9231	0.0702	0.4686	0.4459	0.8171	0.2018	0.9312
ASV_40 (*Bifidobacterium* spp.)	0.7352	0.8632	0.1136	0.1225	0.0271	0.0775	0.8406	0.1933	0.1540	0.5645	0.1728	0.2789	0.6785	0.7979	0.6735	0.6452	0.4009	0.2348	0.3262	0.1499	0.9354	0.0556	0.7035
ASV_42 (*Veillonella atypica*)	0.5866	0.4139	0.8602	0.5270	0.6948	0.8826	0.8026	0.7763	0.2792	0.1232	0.1247	0.3374	0.0714	0.0844	0.6004	0.7096	0.5345	0.4139	0.0534	0.8759	0.4040	0.0862	0.3345
ASV_46 (*Lactobacillus casei* group)	0.1823	0.1511	0.0673	0.9293	0.7442	0.6065	0.0292	0.3330	0.2312	0.3043	0.9563	0.9128	0.3548	0.1951	0.1836	0.3789	0.0199	0.9128	0.0398	0.0802	0.1121	0.3031	0.4039
ASV_50 (*Clostridium* spp.)	0.8080	0.3336	0.1904	0.5905	0.2302	0.2119	0.3396	0.1356	0.7543	0.4850	0.8024	0.7477	0.3427	0.6155	0.8035	0.4850	0.3612	0.1967	0.4586	0.6119	0.3612	0.8466	0.2825
ASV_59 (*Bacteroides* spp.)	0.1547	0.1129	0.0330	0.1176	0.2816	0.1945	0.3362	0.0356	0.7769	0.2642	0.5569	0.0130	0.2044	0.7962	0.5058	0.0314	0.1637	0.6911	0.9309	0.6089	0.1421	0.4316	0.0794
ASV_6 (*Streptococcus salivarius*)	0.8471	0.7908	0.8322	0.9515	0.6788	0.3224	0.9676	0.3392	0.7432	0.9454	0.7394	0.3180	0.4675	0.1178	0.7269	0.2614	0.7308	0.1147	0.3399	0.9252	0.9737	0.7490	0.9585
ASV_61 (*Bacteroides thetaiotaomicron*)	0.1363	0.2641	0.8490	0.8358	0.2697	0.4967	0.9494	0.7348	0.1990	0.1120	0.5902	0.2872	0.0974	0.5214	0.4491	0.6503	0.3709	0.3115	0.1161	0.6564	0.1523	0.0286	0.4288
ASV_63 (*Klebsiella* spp.)	0.4867	0.6032	0.6490	0.2653	0.3819	0.4676	0.4107	0.3229	0.3886	0.3571	0.8248	0.7429	0.3981	0.7875	0.4210	0.3905	0.2020	0.2505	0.4417	0.2972	0.5317	0.4975	0.7187
ASV_7 (*Bifidobacterium longum*)	0.5271	0.6880	0.3251	0.6342	0.9624	0.7164	0.8796	0.3012	0.8886	0.7356	0.4635	0.3755	0.1335	0.0666	0.1057	0.5708	0.0376	0.6296	0.2296	0.6188	0.5492	0.8977	0.8356
ASV_70 (*Bacteroides xylanisolvens*)	0.0033	0.0869	0.8536	0.7064	0.3486	0.4638	0.7660	0.6318	0.2360	0.0467	0.2023	0.1990	0.1058	0.1111	0.1144	0.8677	0.6356	0.1512	0.0367	0.7729	0.0682	0.0304	0.1794
ASV_71 (*Parabacteroides distasonis*)	0.8790	0.8424	0.3778	0.4967	0.5525	0.4833	0.3920	0.7285	0.7832	0.9764	0.5931	0.7541	0.6169	0.8923	0.8490	0.9225	0.5326	0.8756	0.8325	0.9798	0.9764	0.9427	0.9024
ASV_73 (*ClostridiumXVIII ramosum*)	0.2612	0.3907	0.8466	0.8635	0.1635	0.6694	0.5128	0.9144	0.4409	0.2550	0.9857	0.5012	0.7280	0.0178	0.0342	0.6434	0.0455	0.0276	0.0928	0.5679	0.3421	0.0760	0.6932
ASV_77 (*Klebsiella* spp.)	0.5534	0.9119	0.4026	0.5353	0.0690	0.1762	0.7704	0.0760	0.2233	0.2668	0.8693	0.7544	0.1859	0.4725	0.0952	0.1240	0.6552	0.0271	0.0776	0.0622	0.8201	0.2287	0.7193
ASV_86 (*Bacteroides fragilis*)	0.3462	0.3637	0.4455	0.8261	0.8745	0.6336	0.0582	0.8246	0.1087	0.2184	0.7563	0.2719	0.6171	0.4425	0.4089	0.1221	0.3402	0.3957	0.6157	0.2582	0.2608	0.0965	0.3081
ASV_98 (*Bacteroides* spp.)	0.0356	0.2266	0.9943	0.6773	0.9087	0.8917	0.6229	0.8270	0.3459	0.1298	0.0329	0.1670	0.2045	0.6945	0.5316	0.7881	0.8354	0.4897	0.0848	0.9771	0.2195	0.1367	0.2660

FDR corrected P-value[3]

ASV	Diversity index	2'FL (nmol/ml)	3FL (nmol/ml)	LNnT (nmol/ml)	3'SL (nmol/ml)	DFLac (nmol/ml)	6'SL (nmol/ml)	LNT (nmol/ml)	LNFP I (nmol/ml)	LNFP II (nmol/ml)	LNFP III (nmol/ml)	LSTb (nmol/ml)	LSTc (nmol/ml)	DFLNT (nmol/ml)	LNH (nmol/ml)	DSLNT (nmol/ml)	FLNH (nmol/ml)	DFLNH (nmol/ml)	FDSLNH (nmol/ml)	DSLNH (nmol/ml)	Total HMOs (nmol/ml)	Sialylated HMOs (nmol/ml)	Fucosylated HMOs (nmol/ml)
ASV_1 (*Bifidobacterium longum*)	0.9177	0.5734	0.3955	0.7707	0.5617	0.4896	0.3726	0.7011	0.8346	0.6948	0.9825	0.6507	0.7801	0.8661	0.9987	0.7422	0.8376	0.9338	0.7140	0.6413	0.5310	0.9458	0.6350
ASV_108 (*Bifidobacterium* spp.)	0.7329	0.8673	0.8542	0.9515	0.7529	0.6911	0.9830	0.8821	0.8346	0.9759	0.8314	0.8496	0.7801	0.8574	0.9149	0.8330	0.7127	0.9662	0.9457	0.9798	0.7988	0.9458	0.8661
ASV_11 (*Veillonella dispar*)	0.9177	0.8673	0.8542	0.7707	0.7529	0.6606	0.9830	0.8722	0.8346	0.6168	0.8314	0.7074	0.7801	0.8574	0.9149	0.7422	0.8911	0.9338	0.7631	0.8646	0.7988	0.8633	0.7135
ASV_12 (*Streptococcus* spp.)	0.9177	0.8673	0.5539	0.8198	0.8085	0.6606	0.9830	0.8821	0.9807	0.6168	0.9825	0.7074	0.9858	0.8574	0.9987	0.8670	0.7127	0.7810	0.6631	0.9798	0.7988	0.9458	0.8853
ASV_13 (*Escherichia/Shigella* spp.)	0.6058	0.3131	0.3955	0.7707	0.5617	0.6474	0.0188	0.2214	0.7443	0.5293	0.9825	0.2221	0.7801	0.8574	0.9149	0.7422	0.9704	0.9338	0.7631	0.6413	0.3439	0.8633	0.0995
ASV_15 (*Bacteroides vulgatus*)	0.6203	0.5734	0.8385	0.9043	0.9409	0.6911	0.9830	0.8351	0.7443	0.5106	0.3267	0.4912	0.7270	0.8574	0.9987	0.7915	0.8145	0.9338	0.3330	0.9798	0.6378	0.3399	0.7135
ASV_17 (*Bifidobacterium breve*)	0.8939	0.9888	0.3955	0.9043	0.7639	0.6911	0.9830	0.8512	0.9807	0.6168	0.9825	0.6507	0.7270	0.8574	0.9987	0.9408	0.7127	0.9338	0.7631	0.9798	0.9989	0.9458	0.9911
ASV_18 (*Bifidobacterium bifidum*)	0.8939	0.9896	0.8886	0.8198	0.9315	0.8917	0.9830	0.8821	0.8227	0.6948	0.9825	0.6749	0.7801	0.8574	0.9149	0.9408	0.8157	0.9338	0.8469	0.9798	0.9372	0.9458	0.8853
ASV_186 (*Clostridium perfringens*)	0.6058	0.8673	0.8886	0.8867	0.9409	0.6911	0.9830	0.9507	0.8346	0.8623	0.9857	0.8285	0.7801	0.8574	0.9149	0.9408	0.7127	0.7810	0.7631	0.8664	0.7988	0.9458	0.8853
ASV_2 (*Streptococcus mitis* group)	0.6058	0.5734	0.3955	0.7707	0.9159	0.6474	0.4240	0.8821	0.7443	0.5106	0.9825	0.6507	0.7801	0.8574	0.9987	0.9408	0.7127	0.9961	0.3330	0.8646	0.4365	0.5845	0.6350
ASV_21 (*Veillonella* spp.)	0.9470	0.8673	0.8886	0.9043	0.9159	0.7639	0.9830	0.8821	0.7833	0.5106	0.3272	0.6507	0.7801	0.8574	0.9149	0.7915	0.8911	0.6048	0.5431	0.8664	0.9372	0.3399	0.9297
ASV_26 (*Collinsella aerofaeciens*)	0.6058	0.5734	0.3955	0.8674	0.9159	0.6911	0.3726	0.6464	0.8227	0.6168	0.9825	0.6507	0.7801	0.4971	0.9149	0.7984	0.7127	0.6048	0.7140	0.9798	0.4365	0.3399	0.6703
ASV_34 (*Ruminococcus gnavus*)	0.3801	0.5734	0.8542	0.9515	0.7639	0.8626	0.9830	0.8821	0.7443	0.5106	0.9825	0.8285	0.7801	0.8574	0.7616	0.8670	0.7127	0.9961	0.3330	0.8646	0.4365	0.3399	0.6703
ASV_35 (*Bacteroides* spp.)	0.9177	0.8867	0.8886	0.9043	0.7476	0.6606	0.9830	0.8821	0.8713	0.6948	0.9825	0.8285	0.7801	0.4971	0.5474	0.7915	0.7127	0.2942	0.4574	0.7785	0.9372	0.8004	0.7135
ASV_36 (*Enterococcus faecium* group)	0.8229	0.6593	0.8385	0.7707	0.7476	0.6606	0.9830	0.7011	0.9805	0.6948	0.8314	0.6507	0.7801	0.8574	0.9847	0.8670	0.8157	0.9338	0.9796	0.9798	0.6453	0.9458	0.6703
ASV_37 (*Bifidobacterium catenulatum* group)	0.9177	0.8673	0.3955	0.8198	0.9159	0.6911	0.9830	0.8722	0.9807	0.9455	0.9825	0.6507	0.7801	0.8574	0.9987	0.7915	0.7127	0.9338	0.9457	0.9798	0.9372	0.9458	0.8853
ASV_39 (*Enterococcus faecalis*)	0.9177	0.8673	0.8886	0.9043	0.9159	0.6911	0.9830	0.8821	0.7443	0.5106	0.9825	0.6507	0.8364	0.8574	0.9149	0.8670	0.9529	0.5613	0.7140	0.9798	0.9372	0.5381	0.9894
ASV_40 (*Bifidobacterium* spp.)	0.9177	0.9525	0.4038	0.7707	0.5617	0.6474	0.9830	0.7731	0.7443	0.6948	0.8314	0.6507	0.7801	0.8574	0.9796	0.8670	0.7127	0.7288	0.6798	0.7994	0.9989	0.3399	0.8853
ASV_42 (*Veillonella atypica*)	0.8939	0.8278	0.8886	0.9043	0.9159	0.8917	0.9830	0.8821	0.7445	0.5106	0.8314	0.6507	0.7270	0.5385	0.9149	0.9083	0.8145	0.8278	0.3330	0.9798	0.7988	0.3399	0.7135
ASV_46 (*Lactobacillus casei* group)	0.6058	0.5734	0.3955	0.9515	0.9159	0.7764	0.3726	0.8351	0.7443	0.6168	0.9857	0.9128	0.7801	0.7804	0.8391	0.8330	0.4852	0.9736	0.3330	0.6413	0.5310	0.6465	0.8071
ASV_50 (*Clostridium* spp.)	0.9177	0.8213	0.5539	0.9043	0.7529	0.6606	0.9830	0.7011	0.9283	0.6948	0.9825	0.8285	0.7801	0.8574	0.9987	0.8670	0.7127	0.6993	0.7140	0.9798	0.7705	0.9458	0.7135
ASV_59 (*Bacteroides* spp.)	0.6058	0.5734	0.3955	0.7707	0.7529	0.6606	0.9830	0.5699	0.9283	0.6098	0.9825	0.2221	0.7270	0.8574	0.9149	0.7422	0.7127	0.9338	0.9610	0.9798	0.5416	0.8124	0.6350
ASV_6 (*Streptococcus salivarius*)	0.9177	0.9373	0.8886	0.9515	0.9159	0.6911	0.9830	0.8351	0.9283	0.9759	0.9825	0.6507	0.7801	0.5385	0.9847	0.7915	0.8661	0.6048	0.6798	0.9798	0.9989	0.9458	0.9894
ASV_61 (*Bacteroides thetaiotaomicron*)	0.6058	0.7041	0.8886	0.9515	0.7529	0.6911	0.9830	0.8821	0.7443	0.5106	0.9825	0.6507	0.7270	0.8574	0.9149	0.8670	0.7127	0.7810	0.3717	0.9798	0.5416	0.3399	0.8071
ASV_63 (*Klebsiella* spp.)	0.8939	0.8673	0.8886	0.8198	0.7639	0.6911	0.9830	0.8351	0.8227	0.6168	0.9825	0.8285	0.7801	0.8574	0.9149	0.8330	0.7127	0.7288	0.7140	0.8646	0.7988	0.8633	0.8853
ASV_7 (*Bifidobacterium longum*)	0.8939	0.8806	0.8385	0.9043	0.9624	0.8188	0.9830	0.8351	0.9805	0.8623	0.9825	0.6507	0.7270	0.5332	0.7320	0.8670	0.4852	0.9338	0.5653	0.9798	0.7988	0.9458	0.9549
ASV_70 (*Bacteroides xylanisolvens*)	0.1058	0.5734	0.8886	0.9043	0.7639	0.6911	0.9830	0.8821	0.7443	0.5106	0.8314	0.6507	0.7270	0.5385	0.7320	0.9408	0.8376	0.6048	0.3330	0.9798	0.4365	0.3399	0.7135
ASV_71 (*Parabacteroides distasonis*)	0.9177	0.9525	0.8385	0.9043	0.9159	0.6911	0.9830	0.8821	0.9283	0.9764	0.9825	0.8285	0.7801	0.8923	0.9987	0.9408	0.8145	0.9662	0.9457	0.9798	0.9989	0.9458	0.9894
ASV_73 (*ClostridiumXVIII ramosum*)	0.6966	0.8278	0.8886	0.9515	0.7476	0.7934	0.9830	0.9439	0.8227	0.6098	0.9857	0.7074	0.7801	0.4971	0.5474	0.8670	0.4852	0.2942	0.3330	0.9798	0.7705	0.3399	0.8853
ASV_77 (*Klebsiella* spp.)	0.8939	0.9727	0.8385	0.9043	0.5617	0.6606	0.9830	0.6464	0.7443	0.6098	0.9825	0.8285	0.7270	0.8574	0.7320	0.7422	0.8376	0.2942	0.3330	0.6413	0.9372	0.5629	0.8853
ASV_86 (*Bacteroides fragilis*)	0.7914	0.8278	0.8385	0.9515	0.9409	0.7798	0.3726	0.8821	0.7443	0.6098	0.9825	0.6507	0.7801	0.8574	0.9149	0.7422	0.7127	0.8278	0.7631	0.8646	0.6453	0.3399	0.7135
ASV_98 (*Bacteroides* spp.)	0.3801	0.6593	0.9943	0.9043	0.9409	0.8917	0.9830	0.8821	0.7906	0.5106	0.3507	0.6507	0.7270	0.8574	0.9149	0.9408	0.8911	0.9219	0.3330	0.9798	0.6386	0.3976	0.7135

[#]One sample excluded due to maternal antibiotics during sampling

[1]Only ASVs with average relative abundance >0.2% and prevalence >15% across all samples were included

[2]P-values below 0.05 are highlighted

[3]FDR corrected P-values below 0.1 are highlighted

Table S11 - Adonis tests assessing impact of relative abundance of HMOs in feces on microbial community composition in infant feces at age 5 months

HMO category	Weighted Unifrac		Unweighted Unifrac		Bray Curtis		Jaccard	
	R^2	P-value	R^2	P-value	R^2	P-value	R^2	P-value
2'FL/3FL (n=30)	0.1542	**0.011**	0.0702	**0.006**	0.1166	**0.003**	0.0537	**0.004**
LNT/LNnT (n=30)	0.0447	0.272	0.0944	**0.001**	0.0784	**0.018**	0.0703	**0.001**
3'SL/6'SL (n=30)	0.0095	0.834	0.0701	**0.008**	0.0284	0.561	0.0541	**0.001**
2'FL/3FL non-secretors excluded (n=25)	0.1798	**0.017**	0.0875	**0.002**	0.1258	**0.007**	0.0619	**0.004**

Table S12 - Correlations between relative abundances of HMOs in feces and relative abundance of genus level taxa in infant feces at age 5 months

Genus[1]	Correlation coefficient			P-value[2]			FDR corrected P-value[3]			Correlations coefficient	P-value[2]	FDR corrected P-value[3]
	2'FL/3FL (n=30)	3'SL/6'SL (n=30)	LNT/LNnT (n=30)	2'FL/3FL (n=30)	3'SL/6'SL (n=30)	LNT/LNnT (n=30)	2'FL/3FL (n=30)	3'SL/6'SL (n=30)	LNT/LNnT (n=30)	2'FL/3FL when non-secretors are excluded (n=25)		
Bifidobacterium	-0.452	0.053	-0.196	0.012	0.781	0.299	0.098	0.892	0.435	-0.494	0.0121	0.0968
Bacteroides	0.049	-0.111	-0.173	0.795	0.558	0.360	0.884	0.811	0.480	0.051	0.8102	0.9259
Enterococcus	-0.211	-0.153	0.035	0.262	0.420	0.855	0.524	0.719	0.912	-0.052	0.8038	0.9259
Parabacteroides	-0.225	-0.378	-0.414	0.233	0.039	0.023	0.524	0.157	0.122	-0.311	0.1305	0.3680
Klebsiella	0.215	0.555	0.276	0.254	0.001	0.140	0.524	0.012	0.287	0.059	0.7790	0.9259
Veillonella	0.041	0.161	0.147	0.829	0.396	0.437	0.884	0.719	0.538	0.030	0.8867	0.9458
Streptococcus	0.287	0.585	0.576	0.124	0.001	0.001	0.397	0.011	0.014	0.161	0.4427	0.7870
Collinsella	-0.456	-0.408	-0.492	0.011	0.025	0.006	0.098	0.135	0.046	-0.590	0.0019	0.0304
Clostridiaceae_uncl	0.149	0.007	0.208	0.431	0.971	0.269	0.690	0.971	0.431	0.210	0.3145	0.7189
Lactobacillus	-0.383	-0.316	-0.384	0.037	0.089	0.036	0.196	0.284	0.145	-0.430	0.0319	0.1701
Blautia	-0.014	-0.037	-0.223	0.941	0.847	0.236	0.941	0.904	0.420	-0.181	0.3873	0.7746
Clostridium_XVIII	0.052	-0.144	-0.037	0.784	0.449	0.846	0.884	0.719	0.912	-0.097	0.6432	0.9259
Lachnospiraceae_uncl	-0.187	-0.248	-0.367	0.322	0.187	0.046	0.572	0.499	0.148	-0.305	0.1380	0.3680
Escherichia	-0.294	-0.083	-0.274	0.115	0.663	0.143	0.397	0.884	0.287	-0.308	0.1342	0.3680
Clostridium	-0.053	-0.059	-0.002	0.779	0.756	0.990	0.884	0.892	0.990	-0.013	0.9514	0.9514
Anaerostipes	-0.090	-0.156	-0.280	0.635	0.411	0.134	0.884	0.719	0.287	-0.102	0.6263	0.9259

[1]Only taxa with average relative abundance > 0.2% across all samples were included

[2]P-values below 0.05 are highlighted

[3]FDR corrected P-values below 0.1 are highlighted

Table S13 - Correlations between HMOs in feces and relative abundance of ASV level taxa in infant feces at age 5 months

ASV (Assigned taxonomy)[1]	Correlation coefficient			P-value[2]			FDR corrected P-value[3]			Correlations coefficient	P-value[2]	FDR corrected P-value[3]
	2'FL/3FL (n=30)	3'SL/6'SL (n=30)	LNT/LNnT (n=30)	2'FL/3FL (n=30)	3'SL/6'SL (n=30)	LNT/LNnT (n=30)	2'FL/3FL (n=30)	3'SL/6'SL (n=30)	LNT/LNnT (n=30)	2'FL/3FL when non-secretors are excluded (n=25)		
ASV_40 (*Bifidobacterium* spp.)	0.026	-0.078	0.045	0.8898	0.6808	0.8145	0.9430	0.8340	0.8737	-0.096	0.6464	0.7753
ASV_37 (*Bifidobacterium catenulatum* group)	0.021	0.023	-0.306	0.9135	0.9035	0.0997	0.9430	0.9871	0.2959	0.005	0.9813	0.9813
ASV_18 (*Bifidobacterium bifidum*)	-0.450	-0.366	-0.403	0.0125	0.0469	0.0272	0.0829	0.1500	0.1223	-0.442	0.0268	0.1715
ASV_17 (*Bifidobacterium breve*)	0.096	0.112	0.022	0.6125	0.5572	0.9082	0.7539	0.7753	0.9082	-0.053	0.8013	0.8842
ASV_108 (*Bifidobacterium spp*)	-0.230	-0.304	-0.162	0.2211	0.1030	0.3937	0.5443	0.2745	0.6300	-0.254	0.2199	0.5425
ASV_1 (*Bifidobacterium longum*)	-0.471	-0.087	-0.365	0.0086	0.6463	0.0476	0.0829	0.8273	0.1694	-0.505	0.0101	0.1008
ASV_7 (*Bifidobacterium longum*)	-0.294	-0.246	-0.085	0.1152	0.1904	0.6546	0.3552	0.4061	0.8293	-0.310	0.1314	0.5256
ASV_26 (*Collinsella aerofaeciens*)	-0.456	-0.408	-0.492	0.0113	0.0252	0.0057	0.0829	0.1154	0.0608	-0.590	0.0019	0.0608
ASV_98 (*Bacteroides* spp.)	0.126	-0.090	0.060	0.5079	0.6359	0.7547	0.7066	0.8273	0.8625	0.298	0.1483	0.5273
ASV_35 (*Bacteroides* spp.)	-0.145	-0.376	-0.290	0.4431	0.0407	0.1195	0.6977	0.1500	0.3186	-0.132	0.5289	0.7753
ASV_15 (*Bacteroides vulgatus*)	-0.190	0.021	-0.068	0.3136	0.9125	0.7226	0.6272	0.9871	0.8564	-0.254	0.2204	0.5425
ASV_59 (*Bacteroides* spp.)	-0.178	-0.254	-0.399	0.3459	0.1749	0.0289	0.6511	0.3999	0.1223	-0.199	0.3408	0.6059
ASV_86 (*Bacteroides fragilis*)	-0.152	0.003	-0.088	0.4212	0.9871	0.6436	0.6977	0.9871	0.8293	0.208	0.3174	0.5975
ASV_61 (*Bacteroides thetaiotaomicron*)	-0.501	-0.466	-0.305	0.0048	0.0095	0.1017	0.0829	0.0543	0.2959	-0.492	0.0126	0.1008
ASV_70 (*Bacteroides xylanisolvens*)	-0.083	-0.264	-0.146	0.6612	0.1587	0.4409	0.7743	0.3905	0.6718	-0.007	0.9750	0.9813
ASV_71 (*Parabacteroides distasonis*)	0.013	-0.185	-0.204	0.9470	0.3286	0.2799	0.9470	0.6185	0.5454	-0.142	0.4979	0.7753
ASV_39 (*Enterococcus faecalis*)	-0.369	-0.175	-0.200	0.0450	0.3554	0.2897	0.2057	0.6319	0.5454	-0.232	0.2644	0.5641
ASV_36 (*Enterococcus faecium* group)	-0.137	-0.113	0.080	0.4714	0.5511	0.6738	0.6977	0.7753	0.8293	-0.087	0.6784	0.7753
ASV_46 (*Lactobacillus casei* group)	-0.448	-0.372	-0.440	0.0130	0.0427	0.0150	0.0829	0.1500	0.1015	-0.506	0.0098	0.1008
ASV_12 (*Streptococcus* spp.)	0.215	0.462	0.395	0.2548	0.0102	0.0306	0.5824	0.0543	0.1223	0.093	0.6579	0.7753
ASV_2 (*Streptococcus mitis* group)	0.373	0.689	0.670	0.0422	0.0000	0.0001	0.2057	0.0008	0.0016	0.269	0.1935	0.5425
ASV_6 (*Streptococcus salivarius*)	0.288	0.519	0.588	0.1221	0.0033	0.0006	0.3552	0.0264	0.0102	0.233	0.2625	0.5641
ASV_50 (*Clostridiaceae* spp.)	0.143	-0.015	0.241	0.4509	0.9386	0.1987	0.6977	0.9871	0.4542	0.211	0.3111	0.5975
ASV_186 (*Clostridium perfringens*)	-0.103	0.005	0.105	0.5875	0.9773	0.5802	0.7520	0.9871	0.8072	-0.175	0.4041	0.6806
ASV_34 (*Ruminococcus gnavus*)	-0.203	-0.334	-0.437	0.2831	0.0710	0.0159	0.6040	0.2066	0.1015	-0.323	0.1153	0.5256
ASV_73 (*ClostridiumXVIII ramosum*)	0.052	-0.144	-0.037	0.7841	0.4491	0.8464	0.8652	0.7564	0.8737	-0.097	0.6432	0.7753
ASV_21 (*Veillonella* spp.)	0.111	0.133	0.043	0.5601	0.4827	0.8219	0.7468	0.7724	0.8737	0.105	0.6174	0.7753
ASV_11 (*Veillonella dispar*)	0.134	0.072	0.181	0.4797	0.7037	0.3392	0.6977	0.8340	0.5712	0.098	0.6427	0.7753
ASV_42 (*Veillonella atypica*)	0.079	0.229	0.127	0.6775	0.2231	0.5048	0.7743	0.4461	0.7342	-0.042	0.8421	0.8982
ASV_13 (*Escherichia/Shigella* spp.)	-0.322	-0.123	-0.282	0.0828	0.5178	0.1316	0.3209	0.7753	0.3239	-0.317	0.1224	0.5256
ASV_77 (*Klebsiella* spp.)	0.236	0.528	0.213	0.2093	0.0027	0.2586	0.5443	0.0264	0.5454	0.096	0.6492	0.7753
ASV_63 (*Klebsiella* spp.)	0.315	0.531	0.184	0.0902	0.0025	0.3306	0.3209	0.0264	0.5712	0.257	0.2156	0.5425

[1]Only ASVs with average relative abundance >0.2% and prevalence >15% across all samples were included

[2]P-values below 0.05 are highlighted

[3]FDR corrected P-values below 0.1 are highlighted
