# Supplementary material for: Maternal milk microbiota and oligosaccharides contribute to the infant gut microbiota assembly
Source: ISME Commun. 2021 Jun 7;1:21. doi: 10.1038/s43705-021-00021-3 (PMC9723702; doi:10.1038/s43705-021-00021-3)
Supplement: Supplementary file 3 — Supplementary files [file 43705_2021_21_MOESM3_ESM.pdf]

<table>
<tr><th colspan="6">Supplementary File 1: Experimental conditions for the full scan MS mode on UPLC-qTOF-MS (SYNAPT, Waters, Manchester,</th></tr>
<tr><td rowspan="13">Mobile Phases</td><td colspan="5">Five µL of each sample were injected into the gradient mobile phase A (0.1 % formic acid in Milli–Q water), mobile phase B (0.1 % formic acid in ACN:MeOH (70:30, v/v)). Overview of the 7 min gradient is presented below.</td></tr>
<tr><td>Time (min)</td><td>Flow Rate
(mL min^{-1})</td><td>Mobile phase A (%)</td><td>Mobile phase B (%)</td><td>Curve</td></tr>
<tr><td>0</td><td>0.5</td><td>100</td><td>0</td><td>Initial</td></tr>
<tr><td>1</td><td>0.5</td><td>92</td><td>8</td><td>11</td></tr>
<tr><td>2</td><td>0.6</td><td>85</td><td>15</td><td>10</td></tr>
<tr><td>3</td><td>0.7</td><td>60</td><td>40</td><td>10</td></tr>
<tr><td>4</td><td>0.8</td><td>30</td><td>70</td><td>6</td></tr>
<tr><td>4.5</td><td>1</td><td>0</td><td>100</td><td>6</td></tr>
<tr><td>5</td><td>1.2</td><td>0</td><td>100</td><td>1</td></tr>
<tr><td>6.4</td><td>1.1</td><td>0</td><td>100</td><td>1</td></tr>
<tr><td>6.6</td><td>1</td><td>95</td><td>5</td><td>1</td></tr>
<tr><td>6.8</td><td>0.5</td><td>95</td><td>5</td><td>6</td></tr>
<tr><td>7</td><td>0.5</td><td>95</td><td>5</td><td>6</td></tr>
<tr><td>Mass Spectrometry</td><td colspan="5">The electrospray ionization was operated in both positive mode with 3.2 capillary probe voltages, respectively. The cone voltage and the collision energy were set at 25 kV and 6 eV. The ion source and desolvation gas (nitrogen) temperature were 120 and 400 ^{o}C while the sampling cone and desolvation gas flow rates were 50 and 1000 l/hr. The scan time was set at 0.08 s with 0.02 s interscan delay for both modes. Data were acquired in centroid mode with a mass range from 50 to 1000 Da. Leucine-enkephalin (500 ng/ml) was infused as a lock-spray agent to calibrate the mass accuracy every 10s with 0.4s scan time. Data acquisition and visualization were performed in MassLynx V4.1 software (Waters Corporation, Manchester, UK).</td></tr>
</table>

Supplementary File 2: Optimized parameters used for pre-processing the data in mzMine2.28		
Batch step		Parameters
Positive mode	Raw data import	None
	Mass detection	Noise level: 2.0E1
	Chromatogram builder	Min time span (min): 0.02<br>Min height:5.0E1 ; m/z tolerance: 0.04 Da or 20 ppm
	Smoothing	Filter width: 7
	Chromatogram deconvolution	Chromatographic threshold: 95%; Search minimum in RT range (min): 0.01; Minimum relative height: 5%; Minimum absolute height: 6.0E1; Min. ratio of peak/top edge: 1.6; Peak duration range (min): 0.01-0.4
	Isotopic pattern	m/z tolerance: 0.06 Da or 30 ppm; Retention time tolerance: 0.02; Monotonic shape; maximum charge: 1
	Join aligner	m/z tolerance: 0.04 Da or 20 ppm; Absolute retention time tolerance (min): 0.04; Weight for both m/z tolerance and retention time tolerance: 10
	Peak list rows filter	Min peaks in a row: 5 and 0 (for samples and blanks respectively)<br>Minimum peaks in an isotope pattern: 1; m/z range: 50-1000; RT range: 0-7; peak duration range: 0.01-0.4 min
	Peak finder (gap filling)	Intensity tolerance: 50%; m/z tolerance: 0.02 Da or 15 ppm; Absolute retention time tolerance (min): 0.03

Supplementary File 3: Mass spectra of HMO standards

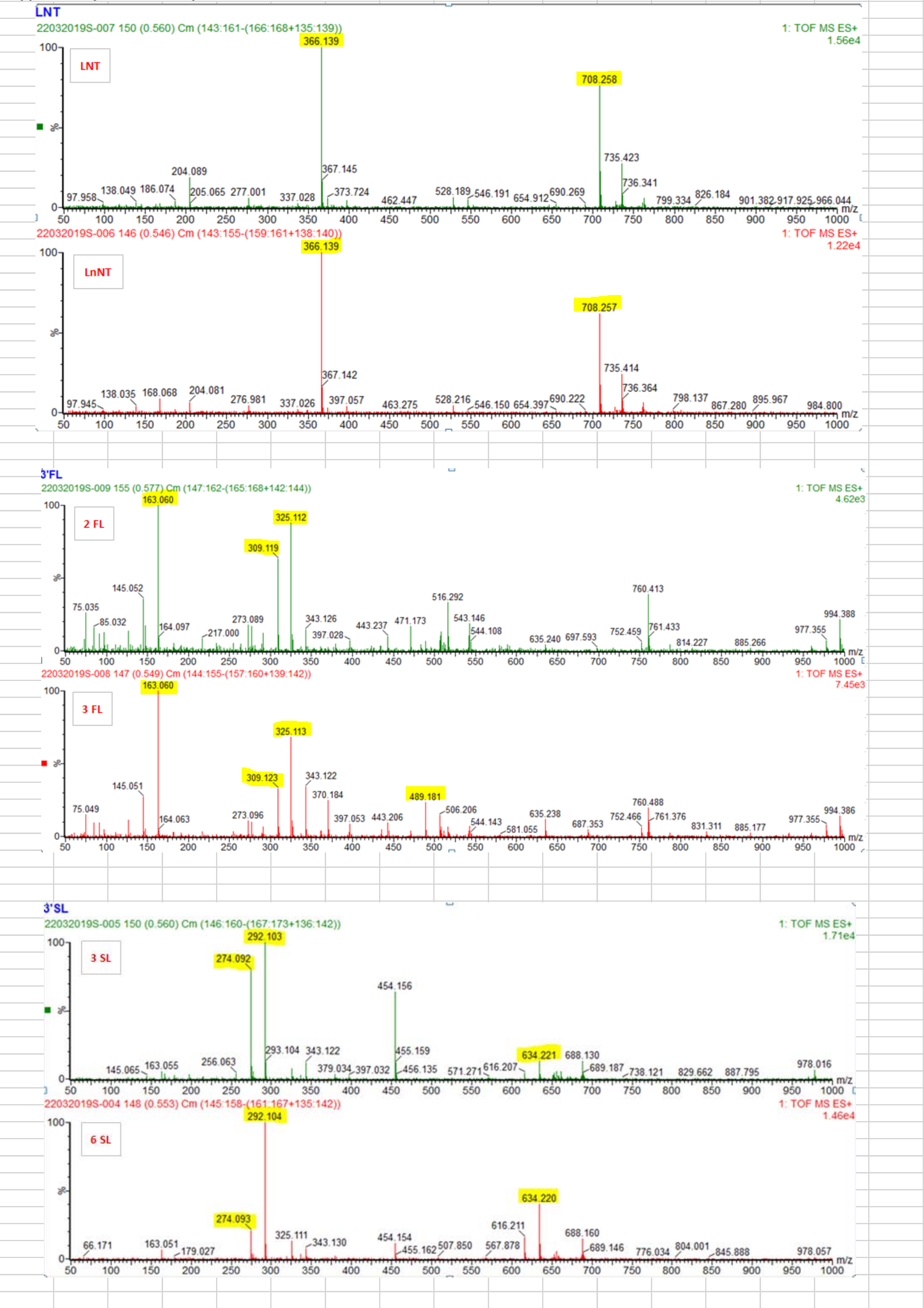

Supplementary File 4: Chromatograms for the major fragment of HMOs in the authentic standards and in infant stool samples

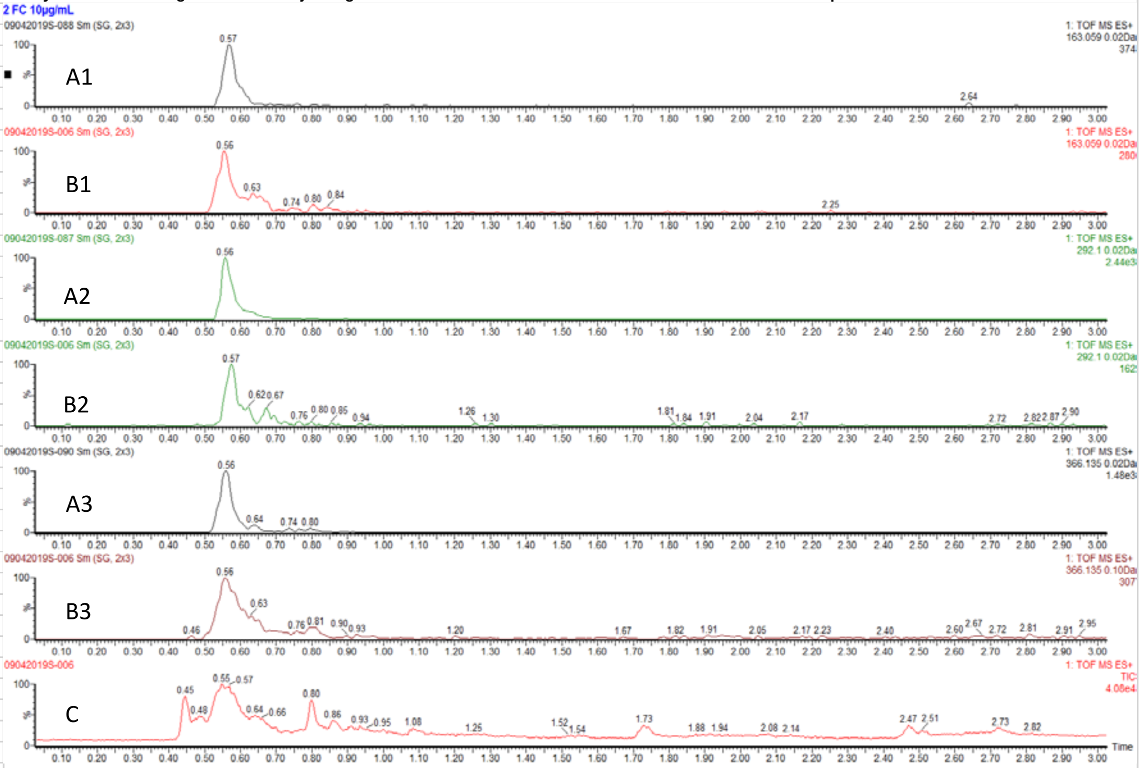

Legend: Chromatograms covering the first 3 min of XICs for major fragment of the HMOs in authentic standards and in stool samples. A1, fragment m/z 163.059 of 3FL standard; B1, fragment m/z 163.059 of 3FL in stool extract; A2, fragment m/z 292.100 of 3'SL standard; B2, fragment m/z 292.100 of 3'SL in stool extract; A3, fragment m/z 366.135 of LNT standard; B3, fragment m/z 366.135 of LNT in stool extract; C, TIC of fecal extract.

Supplementary File 5: Relative abundances (peak intensities) of the HMO residuals in infant stool samples at age 5 months								
Retention Time (min)	0.56	0.56	0.56	0.55	0.57	0.57	0.56	0.56
Mass to charge ratio (m/z)	489.182	325.1119	163.0592*	309.1199	292.1029*	274.0917	708.2556	366.1352*
Subject ID	2FL/3FL [M+H]+	2FL/3FL fragment	2FL/3FL fragment	2FL/3FL fragment	3SL/6SL fragment	3SL/6SL fragment	LNT/LnNT [M+H]+	LNT/LnNT fragment
1	39.0	49.0	85.1	35.5	457.5	930.8	576.4	967.6
2	86.6	63.7	339.2	37.2	932.0	2582.5	59.5	240.0
3	15.0	98.6	220.7	20.0	504.5	1757.2	91.2	378.1
4	27.0	40.5	131.4	20.0	511.1	1358.8	53.0	459.0
5	118.5	467.1	802.1	135.8	588.0	1455.0	219.3	805.5
6	18.0	70.0	85.9	25.0	156.2	282.3	38.7	103.9
7	16.0	24.6	32.8	14.0	32.0	64.1	6.0	26.9
8	15.0	23.0	42.9	11.7	58.4	76.0	10.7	48.0
9	135.3	305.1	641.5	179.3	933.7	2641.3	1501.6	3348.7
10	58.0	85.0	165.1	61.0	337.6	711.8	82.5	625.4
11	29.0	47.1	81.3	33.0	514.4	1190.2	111.3	950.9
12	50.0	34.2	72.7	42.2	428.8	1192.6	204.9	1931.4
13	64.3	250.7	288.3	230.6	106.0	259.3	134.6	973.1
14	20.0	59.9	117.3	32.0	329.9	704.7	64.7	193.2
15	26.0	59.0	51.0	12.5	86.0	131.6	62.3	196.0
16	NA	NA	NA	NA	NA	NA	NA	NA
17	11.0	79.6	66.0	25.7	123.7	184.6	90.1	90.8
18	26.0	35.0	49.8	16.8	109.2	299.1	53.8	246.6
19	9.0	31.0	21.0	40.0	47.7	64.4	27.0	62.3
20	14.7	43.0	61.2	36.2	67.4	158.9	169.0	363.4
21	11.0	19.3	34.0	12.0	48.0	109.5	7.0	57.7
22	29.0	66.0	161.7	22.6	857.4	2683.4	383.6	843.1
23	41.0	155.8	299.0	65.1	1324.0	4817.7	540.4	1790.1
24	20.0	105.9	153.2	79.0	510.6	1677.4	98.2	229.6
25	10.0	27.0	29.5	17.0	23.0	38.0	0.0	23.0
26	504.6	1222.0	1229.1	942.8	184.6	469.4	79.6	620.4
27	701.8	1390.5	2205.6	683.7	377.8	765.3	1592.8	2715.4
28	24.0	77.0	87.8	35.0	55.0	85.1	29.0	133.7
29	800.7	1636.9	1478.9	827.3	97.3	166.7	85.9	488.3
30	NA	NA	NA	NA	NA	NA	NA	NA
31	7.0	25.0	28.0	11.0	80.1	160.0	19.0	14.0
32	11.0	41.1	31.3	11.0	30.6	48.0	33.0	53.0
33	19.0	50.0	27.3	24.0	92.5	126.9	69.9	360.6
34	10.3	63.3	101.6	17.0	70.1	219.4	14.0	45.7

* highest intensity fragment
